# Amino Acid *k*-mer Feature Extraction for Quantitative Antimicrobial Resistance (AMR) Prediction by Machine Learning and Model Interpretation for Biological Insights

**DOI:** 10.3390/biology9110365

**Published:** 2020-10-28

**Authors:** Taha ValizadehAslani, Zhengqiao Zhao, Bahrad A. Sokhansanj, Gail L. Rosen

**Affiliations:** Ecological and Evolutionary Signal-Processing and Informatics Laboratory, Department of Electrical and Computer Engineering, College of Engineering, Drexel University, Philadelphia, PA 19104, USA; tv349@drexel.edu (T.V.); zz374@drexel.edu (Z.Z.); Bahrad@molhealtheng.com (B.A.S.)

**Keywords:** antimicrobial resistance, machine learning, genome sequencing, *k*-mer counting, nucleotide, amino acid, gene clustering, SNP

## Abstract

**Simple Summary:**

Infectious bacteria (microbes) are able to evolve to become resistant to antibiotics (develop antimicrobial resistance, or AMR). Resistant microbes are harder to treat, requiring higher doses, or alternative medications, which can be more toxic. Because of inappropriate use of medicine, microbes are being subjected to evolutionary pressure resulting in increased AMR development. As a result, AMR is emerging one of the biggest public health challenges of our time—posing the risk of a pandemic without effective treatment or vaccine. The goals of this paper are to develop and analyze machine learning methods to use the genome sequence information of a bacterium to: (1) predict the minimum required dose of an antibiotic to treat bacterial infection, and, (2) identify specific mutations or altered genetic content give rise to AMR. In particular, we propose a novel method to apply machine learning algorithms to learn patterns of amino acid sequences in the genes of the bacteria. We show that our proposed method produces comparable or even more accurate results when compared to existing methods for the goal of dose prediction, and it can provide additional insight for scientists who study AMR mechanisms.

**Abstract:**

Machine learning algorithms can learn mechanisms of antimicrobial resistance from the data of DNA sequence without any a priori information. Interpreting a trained machine learning algorithm can be exploited for validating the model and obtaining new information about resistance mechanisms. Different feature extraction methods, such as SNP calling and counting nucleotide *k*-mers have been proposed for presenting DNA sequences to the model. However, there are trade-offs between interpretability, computational complexity and accuracy for different feature extraction methods. In this study, we have proposed a new feature extraction method, counting amino acid *k*-mers or oligopeptides, which provides easier model interpretation compared to counting nucleotide *k*-mers and reaches the same or even better accuracy in comparison with different methods. Additionally, we have trained machine learning algorithms using different feature extraction methods and compared the results in terms of accuracy, model interpretability and computational complexity. We have built a new feature selection pipeline for extraction of important features so that new AMR determinants can be discovered by analyzing these features. This pipeline allows the construction of models that only use a small number of features and can predict resistance accurately.

## 1. Introduction

Antimicrobial resistance (AMR) is a growing global threat [1,2,3,4,5,6]. AMR causes at least 700,000 deaths annually, a number that is projected to increase to 10 million by 2050 if no action is taken [6,7]. Even as the world contends with the reality of viral pandemics like COVID-19, influenza and Ebola, the world may soon face the scenario where bacterial infections, again, become a leading cause of death for humanity [4]. The annual cost of antibiotic-resistant infections to the US health care system is $55 billion [8]. The cumulative cost of combating AMR by 2050 is estimated between 60 and 100 trillion US dollars [7]. Antibiotics can cause different types of drug-induced toxicity, such as nephrotoxicity [9] and hepatotoxicity [10]. Thus, prescribing the correct amount of the correct antibiotic to treat a bacterial infection with minimizing the negative side effects is of seminal importance.

Advances in genomics can expedite the process of AMR detection. Traditional methods of antimicrobial susceptibility testing require that bacteria are first isolated from human specimens by culture techniques, and then, in multiple assays, isolated bacteria are exposed to different concentrations of antibiotics to find out which concentration inhibits growth [11,12,13]. These approaches are slow and expensive [14,15]. Alternatively, genome sequencing methods can be used for AMR prediction [14,16]. Prediction of AMR from the genome of the bacteria is faster than culture-based methods: While results of traditional tests are not available for days after sample collection [17], commercial polymerase chain reaction (PCR) methods have been able to reduce the time of AMR prediction to 2 h [18]. Moreover, the continuous reduction in the cost of sequencing technologies is making this solution even more attractive.

Methods of AMR prediction from the genome of a microbe can be broadly categorized into two groups: methods based on preexisting knowledge of genetic AMR determinants [19,20,21], and methods with no a priori knowledge of AMR determinants [15,22,23,24]. A priori knowledge-based methods predict AMR by cross referencing the genome sequence of the bacterium against databases of known AMR genes and mutations [25]. Databases such as PAThosystems Resource Integration Center (PATRIC) [26], Comprehensive Antibiotic Resistance Database (CARD) [27], RssFinder [28], PointFinder [29] and Antibiotic Resistance Gene-ANNOTation (ARG-ANNOT) [30] provide a large number of known AMR determinants. The main limitation of a priori knowledge-based methods is that they are only effective when the AMR mechanisms are known [24,25]. Additionally, those methods generally assume that a single genetic factor is causing AMR, or, if multiple factors are present, that they do not interact [25]. In other words, these methods do not account for epistasis between AMR mechanisms; however, epistasis has been reported to alter the final AMR phenotype when different mechanisms co-exist [31,32,33]. On the other hand, methods that do not require a priori knowledge of the identity of specific genetic AMR determinants, such as [15,22,23,24,34], infer AMR phenotype from genome sequence data using statistical techniques and machine learning. Using the advances in machine learning, these methods are capable of learning complex interactions between different genetic AMR factors. One notable characteristic of those machine learning models which do not require a priori knowledge is that they learn the AMR knowledge from the data. As a result, scientists can use these models not only for AMR prediction, but also for discovering new AMR mechanisms that have not been discovered before, given that the model can be interpreted.

AMR can be measured as a continuous variable or as a discrete variable. Certain methods [23,35,36,37,38,39,40,41] classify the genomes into two classes of resistant or susceptible with respect to a certain antibiotic. For example, in the case of [22], mapping was to three classes: resistant, intermediate and susceptible for *Acinetobacter baumannii*, *Staphylococcus aureus*, *Streptococcus pneumoniae* and *Mycobacterium tuberculosis*. In cases of [36,39], the intermediate class was merged with the resistant class (for AMR predicting of *Escherichia coli*, in [36] and for AMR prediction of *Staphylococcus aureus*, *Pseudomonas aeruginosa* and *Escherichia coli*, in [39]). On the other hand, methods such as [15,19,20,24,42,43] use machine learning to predict the minimum inhibitory concentration (MIC) of an antibiotic on a certain strain. These papers predicted MICs of different antibiotics for *Streptococcus pneumoniae* [42], *Neisseria gonorrhoeae* [19], Klebsiella pneumoniae [15] and Salmonella [24], and the MIC of ciprofloxacin for *Escherichia coli* [20]. MIC is defined as the minimum concentration of an antibiotic that will inhibit the visible growth of a microorganism after overnight incubation [44]. One shortcoming of methods that predict resistance or susceptibility is that the definition of a resistant or susceptible strain depends on clinical breakpoints (thresholds) [45,46]; however, in some cases, there is no agreement on the values of these breakpoints between scientific institutions [45]. Moreover, the breakpoints are subject to change over time [47], resulting in different definitions of resistance or susceptibility to an antibiotic. On the other hand, MIC prediction does not rely in on a breakpoint. MIC also provides some resolution as to the reflection of level of resistance, rather than a binary output of resistant or susceptible. As a practical example, this can help distinguishing between a strain that has a very low MIC and a strain that is susceptible but has an MIC that is close to the breakpoint [19]. From the machine learning point of view, in these methods quantitative MIC prediction is a regression problem, meaning that a continues value is outputted by the machine learning algorithm, which is the MIC, unlike classification problems where a discrete label such as 0 for susceptible or 1 for resistant was outputted.

Different papers have used different feature extraction methods to present genome sequence data as input to prediction models such as support vector machines [38,48,49], neural networks [41], or gradient boosting [15,24,36], for AMR prediction. In [50,51], for example, the authors used, as features, single nucleotide polymorphisms (SNPs) in genes that previously were known to have drug-resistance mutation for *Mycobacterium tuberculosis*. In another paper [14], the authors also used SNPs to predict resistance in *Mycobacterium tuberculosis*, in two scenarios: only using SNPs in known AMR genes and using all SNPs in the dataset. In [34], SNPs from whole-genome sequence (WGS) to predict resistance for *Neisseria gonorrhoeae*. In [35], the following feature extraction method was used for binary AMR prediction in *Escherichia coli*: The authors first clustered all genes in all genomes using 95% amino acid identity. Genes that existed in all genomes were labeled as “core” genes, and genes that existed in some genomes were labeled as “accessory” genes. Then, a genetic algorithm (GA) method was used to choose which subset of accessory genes should be used for AMR prediction. Finally absence or presence of chosen accessory genes in each genome was used as feature to predict AMR. Khaledi et al. [38] tried gene expression, gene absence/presence, SNP calling and different combinations of those features for binary AMR prediction in *Pseudomonas aeruginosa*. For gene absence/presence, they clustered all of the genes with the condition of 95% sequence alignment coverage (genes went to the same cluster only if there was 95% coverage when they aligned using BLAST). Then absence or presence of each gene cluster in each genome was used as a feature. Hyun et al. [39], used absence/presence, but since gene clustering ignores genetic variation, they also included unique amino acid sequence variants or “alleles” of each gene. In their definition a core gene was a gene that was missing in at most 10 genomes. Similarly, in [37] both SNPs and gene absence/presence were used to predict AMR for *Elizabethkingia*. Reference [36] used different features, including absence/presence of accessory genomes, SNPs, indels and year of isolation for *Escherichia coli*.

Another approach has been to count nucleotide *k*-mers (oligonucleotides) for predicting MIC: For example, ref. [15,24] employed counts of 10-mers (subsequences of length 10) for *Klebsiella pneumoniae* and 15-mers for *Salmonella enterica*, respectively. In [22,23], nucleotide 31-mers were employed to predict resistance for multiple species. Liu, et al. [52] also used nucleotide 31-mers to predict resistance in *Actinobacillus pleuropneumoniae*. In [49], nucleotide *k*-mers of length 5 to 10 were used for *Neisseria gonorrhoeae*. Although nucleotide *k*-mers can perform well in terms of predicting AMR accurately, good performance is only guaranteed when the *k*-mer is long enough. Furthermore, important features (*k*-mers) chosen by the model in this method can only be interpreted by searching against databases such as NCBI [53] if *k* is long enough. However, simply increasing *k*-mer length runs into a substantial practical problem: the number of features in this method increases exponentially as *k* increases, at least before the number of features becomes limited by the sizes of the genomes. As such, longer *k*-mers can lead to memory issues when a machine learning model is trained using these features.

To mitigate the problems associated with increasing *k*-mer length to achieve interpretability, in this paper we propose an alternative: counting amino acid *k*-mers (i.e., oligopeptide sequences) in protein sequences. Amino acid *k*-mer counts exploit the biological redundancy of the nucleotides sequences to provide a more compact representation of the data. Amino acid *k*-mers can provide easier model interpretation and require less computational complexity compared to nucleotide *k*-mers, while providing comparable accuracy to other feature extraction methods evaluated herein, such as counting nucleotide *k*-mers of varying lengths, identifying the absence/presence of gene clusters and obtaining SNPs.

We principally used extreme gradient boosting (XGBoost) regression [54] to train the models evaluated in this paper. In XGBoost, several trees are trained to learn the relationship between input and output. After being trained, each tree calculates the output by comparing the inputs to a series of thresholds in a hierarchical manner. Each tree attempts to correct mistakes of the previous tree. The final output is sum of predictions from all trees. As a result, in XGBoost, a strong learner is built by combining decisions of several weak learners [54].

In this paper, we first use amino acid *k*-mers alongside other feature extraction methods to predict MIC and compare them in terms of ability to predict MIC, interpretability, feature stability and computational complexity. Then, we build and demonstrate the results of a feature selection pipeline for the extraction of important features, and use it to identify important AMR determinants from the model without any prior knowledge about AMR mechanisms. For example, the pipeline discovers that although the truncated version of tetracycline resistance gene tet(D) is known as a resistance conferring gene in PATRIC database, *Klebsiella pneumoniae* strains that have this version have lower MICs compared to strains that do not have any version of this gene. Finally, we show that we can build a model with only a few important features picked by our feature selection pipeline, which in many cases reaches a better accuracy in comparison with a model that uses all features. In this paper, we apply the proposed feature extraction method as well as other existing feature extraction methods to four gram-negative bacteria species.

## 2. Methods

### 2.1. Data Acquisition and Pre-Processing

We applied all feature extraction methods described below to four bacterial species, namely, *Campylobacter jejuni*, *Neisseria gonorrhoeae*, *Klebsiella pneumoniae* and *Salmonella enterica*. For *S. enterica* the MIC values were downloaded from the published metadata of a previous study [24] and for *K. pneumoniae* the MIC values were downloaded from the metadata of another study [15]. For *C. jejuni* and *N. gonorrhoeae*, the MIC values were downloaded from the PATRIC database [26]. For all datasets, the nucleotide sequences and the amino acid sequences were obtained from PATRIC database. Nucleotide genomes were in FASTA format (.fna) and amino acid sequences were in protein FASTA format (.faa). The amino acid sequences were annotated using RAST tool kit (RASTtk) [55] by PATRIC. For dual antibiotics, such as trimethoprim–sulfamethoxazole, we used the MIC values of the first antibiotic, because the second one either depends on the first one or is constant [15]. For each antibiotic and each species, we discarded MIC target values which were underrepresented below a specified threshold in the amount of strains. We set the threshold for discarding a target value to 3, meaning that strains of any target value that had only 1, 2 or 3 strains were discarded from dataset of that particular species of antibiotic combination. The number of genomes and distribution of MIC values for each species and each antibiotic are provided in Appendix A. Chemical structures of all antibiotics are provided in Appendix J.

### 2.2. Feature Extraction Methods

We compared the results of the following feature extraction methods: counting nucleotide *k*-mers in raw DNA sequences, counting amino acid *k*-mers in protein sequences annotated by PATRIC from the raw DNA sequences, gene content (finding which genes exist in which genomes, and in a case of existence, how many times the gene is present in the genome), SNP calling and the combination of gene content and SNP calling.

#### 2.2.1. Nucleotide *k*-mers

Nucleotide *k*-mers were counted using KMC3 [56] for the genome of each strain. The minimum count for output *k*-mers was set to 1, meaning that any *k*-mer with a frequency more than zero was counted. By default, the maximum count for a *k*-mer in KMC3 is 255. In order to avoid cutting off the counts when a *k*-mer repeated more than this value, we set the maximum value to 4,294,967,295, which is larger than any possible *k*-mer count in our data because the longest genome in our data had 9,985,884 characters and a *k*-mer count theoretically cannot be larger than that. *k*-mers of length 8, 9, 10 and 11 were tested. We did not try longer *k*-mers because of memory limitation.

Two scenarios are possible for counting nucleotide *k*-mers. One scenario is to convert all of the non-canonical *k*-mers (*k*-mers that have smaller lexicographical orders than their reverse compliments [57]) to their reverse complement to get the canonical form, and just count canonical *k*-mers. The other scenario is to count all *k*-mers [57]. For each *k*, both scenarios were tested in Appendix F. Results show that converting non-canonical *k*-mers to canonical performs better. Therefore, canonical counting was used for all nucleotide *k*-mer analyses in the main text. The rest of the parameters were set to their default values.

#### 2.2.2. Amino Acid *k*-mers

Amino acid *k*-mers were counted in the protein FASTA sequences downloaded from PATRIC database. For counting the amino acid *k*-mers, we used MerCat [58]. The minimum frequency for output *k*-mers was set to 1, so that any existing *k*-mer was counted; 3-mers, 4-mers and 5-mers of amino acid (which correspond to 9-mers, 12-mers and 15 mers of nucleotide, respectively) were counted for the genome of each strain. We did not try longer *k*-mers because of our memory limitation.

#### 2.2.3. Gene Content

The goal of this method is to predict MIC based on the gene content of the strains. The gene content of a genome is defined as the set of genes that exist in that genome and the number of times each gene exists. In [35] a subset of genes was selected by a GA method to be used for AMR prediction. On the other hand, [36,38,39] used all genes as features. We chose the second approach, which includes all genes. Moreover, in [35,36,38,39], the clustering of the genes was performed on all genomes regardless of partitioning of the genomes into training and testing sets. This creates a bias for the machine learning model because the model is exposed to some information about the test data during the clustering step before training. To avoid this bias, we performed clustering one the training data and searched for the genes in the test set.

For each species and each antibiotic, in order to find an orthologous gene in different genomes, in each training set, we combined all of the genes in amino acid sequences of all of the training genomes into one FASTA file. Then the entries of this FASTA file were clustered using MMseqs2 version 9.d36de [59,60]. We used the easy-linclust command with default settings: Setcover clustering mode, maximum *e*-value: 0.001, minimum alignment length 80%, amino acid substitution matrix: Blosum62. After performing the clustering, the list of all existing gene clusters was extracted. Each cluster was labeled with the PATRIC ID of a representative gene sequence. For each genome, the number of times each gene cluster was observed was counted to create the gene cluster feature matrix. In this matrix, each row is a genome and each column is a gene, and the corresponding element is the number of times that gene exists in that genome. We used count rather than binary absence/presence so that the model could understand if a gene was observed more than once in a genome. After training the model to search existence and counts of the genes in the test genomes, we again used MMseqs2 with the same parameters (maximum *e*-value: 0.001, minimum alignment length 80%, amino acid substitution matrix: Blosum62) to search the test genomes in the training genomes. By doing this, we made sure that the training set and the test set were completely separated and the model was been exposed to any information about the test genomes during the training or before that.

#### 2.2.4. SNP Calling

The goal of this method is to predict AMR based on the SNPs in the genome. There are multiple software packages for SNP calling used in the literature [61,62,63,64]. Based on the comparative analysis of [65], Snippy [66] had the best overall rank when the reference genome was divergent from the sources of the reads. Thus, we chose Snippy in our analysis. For the genome sequence of each strain, SNPs were extracted by comparing each genome to the reference sequence. The reference sequences were downloaded from National Center for Biotechnology Information (NCBI) [53] in FASTA format. The NCBI accession IDs of the chosen reference genome for different species are as follows. *C. jejuni*: NC_002163, *K. pneumoniae* NC_016845, *N. gonorrhoeae*: NC_002946.2 and *S. enterica*: NC_003198.1. We used Snippy version 4.6.0 [66] for extracting SNPs. Since genes might be at different locations in query genomes with respect to reference genome, Snippy shreds each query to 250 bp pseudo-reads with 20x coverage and then aligns the short pseudo-reads to the reference and finds SNPs using Freebayes [62] (version: 1.3.2-dirty). We annotated the SNPs with respect to the reference genome. In each position on the reference, the nucleotide could change to three different bases. For example, If at a position 200, the reference has A, the variant at that position can be C, T or G. In order to be able to keep all of the information, we one-hot coded the SNP features. For example, For the aforementioned position, 3 features can exist: position 200 > C, 200 > G, and 200 > T. Snippy also finds indels that we did not use.

This representation of data leads to a sparse matrix (more than 95% zero), because many SNPs do not exist in most of the strains. We exploited this criterion of the data to conserve memory, by storing the data in form of sparse row matrix of SciPy (version 1.3.0) [67].

#### 2.2.5. Gene Content and SNP Calling

Both the gene content and SNP features were combined and tested as another feature extraction method. In this feature extraction method, SNP features extracted according to Section 2.2.4 and gene content features extracted according to Section 2.2.3 were concatenated into one vector. Since most of the features for this data were the SNP features, the data were also sparse and we used the same sparse representation as Section 2.2.4.

### 2.3. Feature Matrix and Target Values

For each species and each antibiotic regardless of the feature extraction method, certain features only exist in certain genomes and not all of them. For example, for *k*-mer features, a genome of one strain has a subset of all possible *k*-mers and the genome of another strain has a different subset. To build a unified feature matrix, the union of all existing features was calculated to create the feature space. The feature matrix was created as a $N_{feat}\times N_{strain}$ matrix, where $N_{feat}$ is the total number of existing features and $N_{strain}$ is the number of genomes in the dataset. The feature matrix was used for machine learning in conjunction with the target MIC values.

Similarly to [15,24], when the MIC value was larger than the maximum testing threshold (reported as MIC>x) the employed MIC value was replaced with 2×x, and when MIC value was smaller than the minimum testing threshold (reported as MIC<x), it was replaced with x/2. In all cases the target MIC values were converted to $log_{2}$ scale for the machine learning task. Without this conversion, the model will not be able to distinguish the differences between different small MIC values in the presence of larger values. For example, small target values, such as 0.125 and 0.25, look the same in the presence of large target values, such as 64 and 128, because the difference between two small target values looks like a small amount of noise compared to the large target values. After the conversion, the mentioned target values become −3, −2, 6 and 7 respectively.

**Figure A1:**
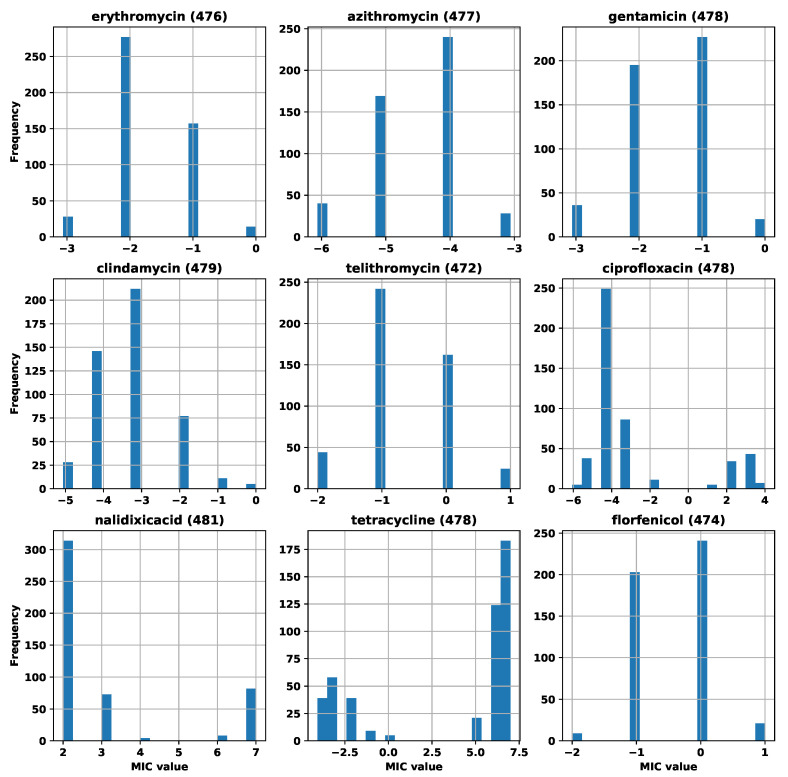
Distribution of *C. jejuni* MIC values for different antibiotics. These are the values after processing and conversion to $log_{2}$ scale. The antibiotic’s name is printed on the title of each sub-figure. The number of genomes is presented in parentheses.

**Figure A2:**
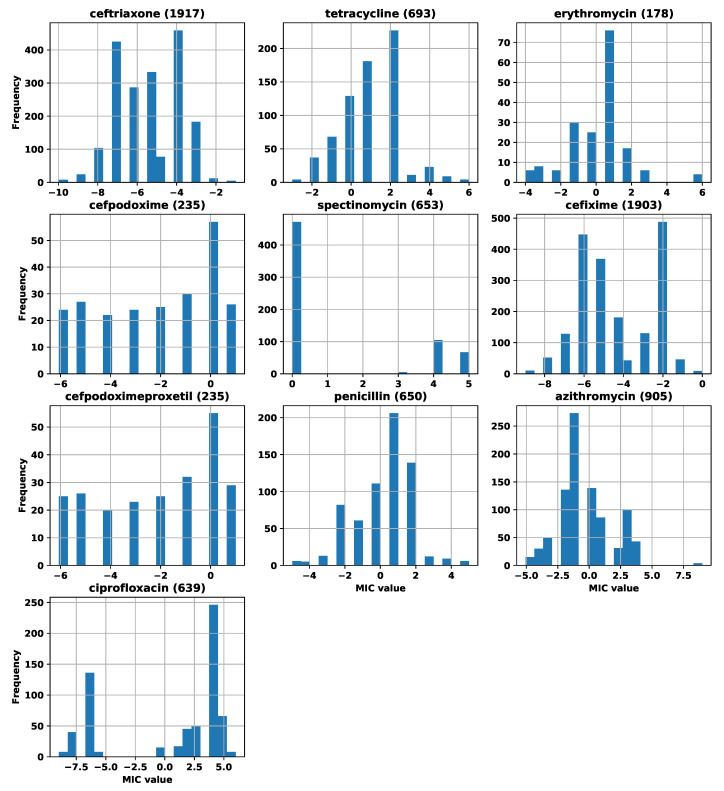
Distribution of *N. gonorrhoeae* MIC values for different antibiotics. These are the values after processing and conversion to $log_{2}$ scale. The antibiotic’s name is printed on the title of each sub-figure. The number of genomes is presented in parentheses.

**Figure A3:**
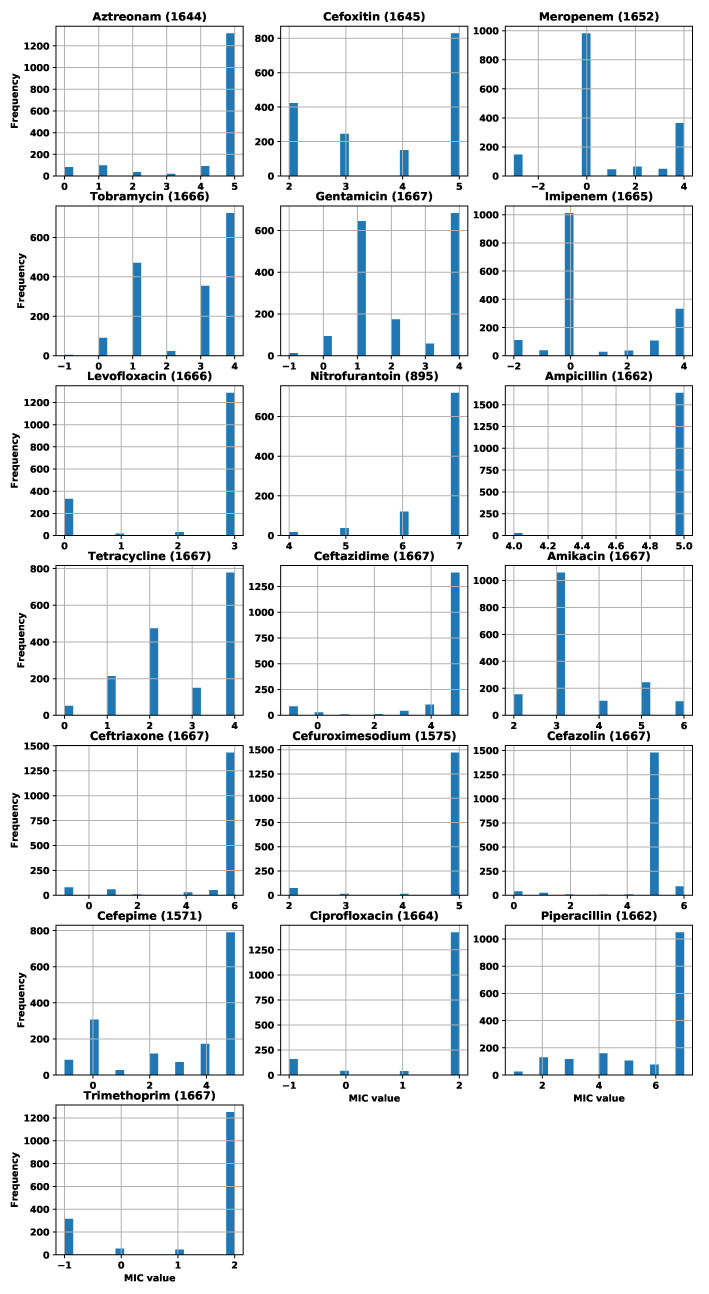
Distribution of *K. pneumoniae* MIC values for different antibiotics. These are the values after processing and conversion to $log_{2}$ scale. The antibiotic’s name is printed on the title of each sub-figure. The number of genomes is presented in parentheses.

**Figure A4:**
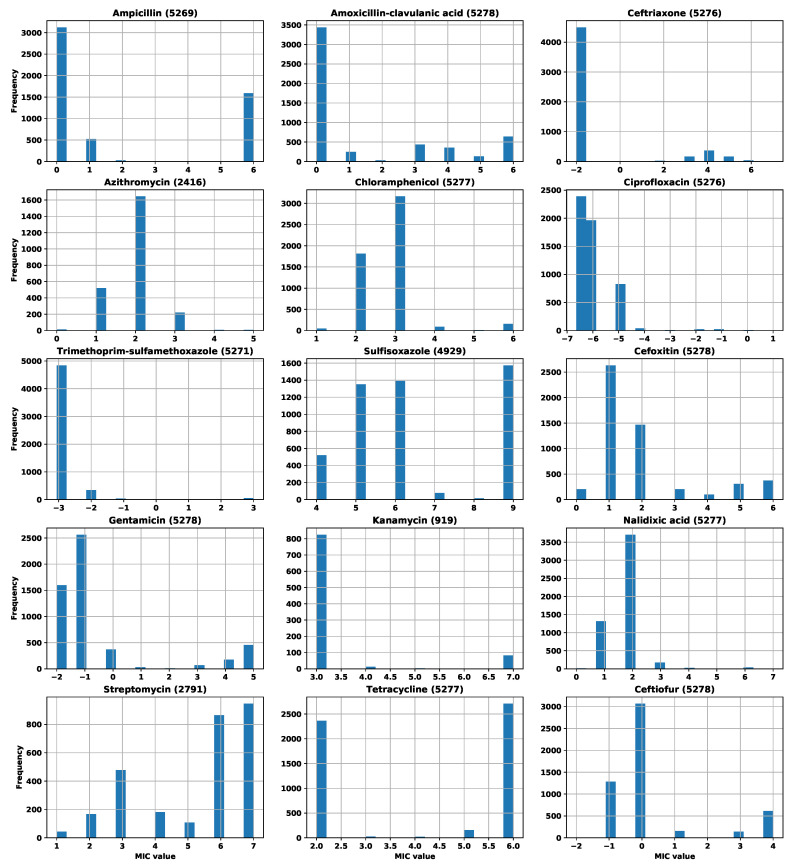
Distribution of *S. enterica* MIC values for different antibiotics. These are the values after processing and conversion to $log_{2}$ scale. The antibiotic’s name is printed on the title of each sub-figure. The number of genomes is presented in parentheses.

Distribution of MIC values For each species–antibiotic combination is presented in Figure A1, Figure A2, Figure A3 and Figure A4.

**Figure 1:**
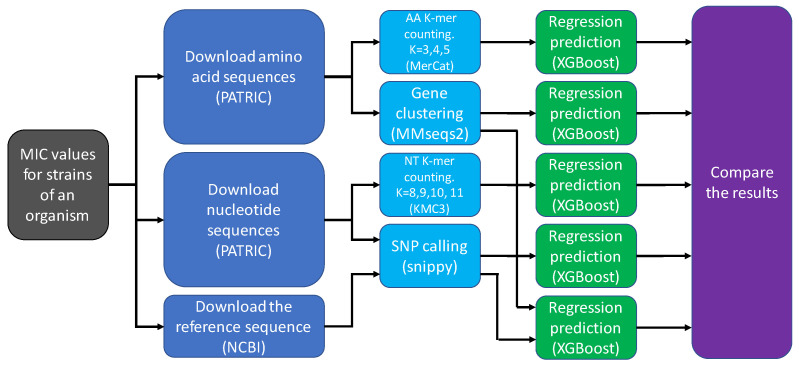
Overall pipeline for all feature extraction methods.

The overall pipeline for all feature extraction methods is depicted in Figure 1.

### 2.4. Measuring the Prediction Performance

According to the FDA standards for antimicrobial susceptibility test [68], essential agreement (EA) between two MIC measurement approaches is achieved when MIC of the proposed method is within ±1 two-fold dilution of the reference method. In other words, if the reference MIC is denoted by *x* and the MIC of the proposed method is denoted by $\tilde{x}$, the agreement is reached when

(1) $$\tilde{x}\in \left[x/2,2x\right]$$

This can be used to evaluate performance of MIC prediction methods: ±1 two-fold accuracy is defined as the number of predictions that satisfy EA, divided by the total number of predictions, as done in [15,19,24,42,43]. Note that in the $log_{2}$ scale the accepted range of EA becomes $\left[log_{2}\left(x\right)-1,log_{2}\left(x\right)+1\right]$. We used this metric as the main method to evaluate the the prediction performance. As an alternative that tolerates greater error, ±2 two-fold dilution accepts anything in the range of [x/4,4x] as a correct prediction for the actual target value of *x*.

A more theoretical alternative metric is root mean squared error (RMSE). For a set of target values, such as $x_{i}$, and a set of corresponding predictions, such as ${\tilde{x}}_{i}$, RMSE is defined as:

(2) $$RMSE=\sqrt{\sum_{i}{\left(x_{i}-{\tilde{x}}_{i}\right)}^{2}}$$

RMSE is more precise compared to ±1 two-fold accuracy, because it better quantifies the error.

Two other biologically important metrics are major error (ME), which is type I error, and very major error (VME), also known as type II error [68]. These metrics can be calculated based on breakpoints for MIC. Breakpoints are concentrations (mg/L) of an antibiotic that define whether a strain is susceptible or resistant to the antibiotic. If the MIC is less than or equal to the susceptibility breakpoint, the strain is considered susceptible to the antibiotic. If the MIC is greater than the resistance breakpoint, the strain is considered resistant to the antibiotic. If MIC is between the susceptibility and resistance breakpoints, the strain is considered intermediate. For *C. jejuni* and *S. enterica*, the breakpoints were obtained from the National Antimicrobial Resistance Monitoring System for Enteric Bacteria (NARMS) [69]. For *N. gonorrhoeae* and *K. pneumoniae*, breakpoints were obtained from the Clinical and Laboratory Standards Institute (CLSI) [46]. Predicting a truly susceptible strain as resistant is a ME and predicting a truly resistant strain as susceptible is a VME. Rate of ME and VME can be calculated by dividing the number of errors by the total number of tested strains.

We report a performance evaluation with all of the metrics for all of the species–antibiotic combinations in Appendix E.

### 2.5. Machine Learning

For each species and each feature extraction method, we trained a separate model for each antibiotic. Similarly to methods in [15,19,20,24,43], we trained the model to predict MIC as a regression task, rather than just classifying the genomes into resistant or susceptible.

In order to choose the best regression package, different methods were tested on an experimental dataset, with *S. enterica* and ampicillin, 4-mers of amino acid, to see which one performed better. After separating 10% of the data as a hold-out set, the remaining genomes were divided into 10 folds of stratified cross-validation. Regression packages that we tried were linear regression, ridge regression, support vector machine regression with three kernels (linear, radial basis function or RBF, and polynomial), random forest regression [70], AdaBoost regression [71] and XGBoost regression [54]. For XGBoost, we used the Python implementation version 1.0.2 [72] with regression objective function and squared loss. For all other methods, we used scikit-learn version 0.23.1 [73]. In this experiment, all methods were trained with their default parameters and no hyper-parameter tuning was performed. Results of the comparison are provided in Section 3.1.2. Since XGBoost performed better than all other methods and had a reasonable computational complexity, we chose XGBoost.

#### 2.5.1. Hyper-Parameter Tuning For XGBoost

**Figure 2:**
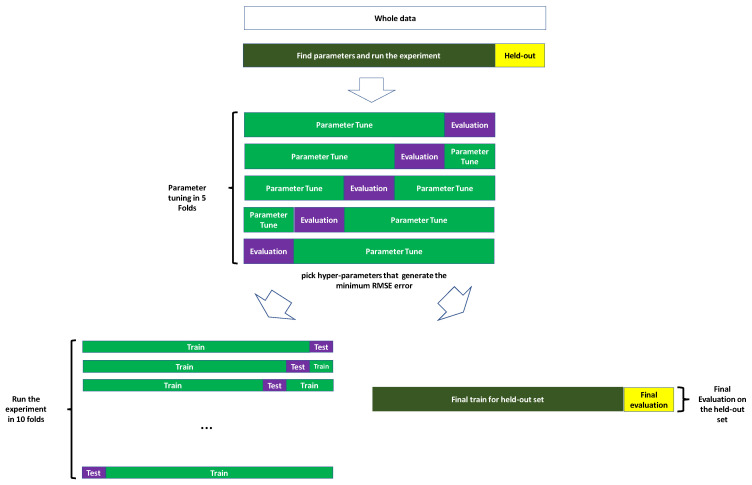
Overall pipeline: First, a hold-out set is separated for the final evaluation. Then for each microbe and each feature extraction method, hyper-parameter tuning is done on one antibiotic with 5 folds of cross-validation, which results in 5 different sets of hyper-parameters in the end. The parameter set that minimizes the RMSE is chosen for 2 experiments: Cross-validation using 10 folds, and a final evaluation on the hold-out set.

After selecting XGBoost, in order to find the best combination of hyper-parameters, we performed hyper-parameter tuning for each species and each feature extraction method separately. Since we had four species and five feature extraction methods, the hyper-parameter tuning was performed 20 times. For *k*-mer counting feature extraction methods, different *k*-mer lengths exist. We ran hyper-parameter tuning on 4-mers of amino acids and 10-mers of canonical nucleotides. Since different antibiotics exist, For each species, one antibiotic was used for tuning. For *C. jejuni* we used clindamycin; for *N. gonorrhoeae*, we used tetracycline; for *K. pneumoniae*, we used cefoxitin; and for *S. enterica*, we used streptomycin. These antibiotics were chosen because they each had (i) a relatively uniform distribution of MIC values, (ii) a large range of values and (iii) a sample size large enough to allow the model can learn the data while being small enough to avoid computational complexity. Before performing hyper-parameter tuning, the genome of each microbe was divided into two parts: 90% of the genomes were used for running the experiment, and 10% were held out (see Figure 2). For each microbe, the hold-out set was not used at any of the hyper-parameter tuning or cross-validation steps and was saved exclusively for the final evaluation. Since there are different number of genomes for different antibiotics, we could not use the same hold-out set for all antibiotics. We also ensured that no genome in the hold-out set would ever be observed by any of the models during the parameter-tuning step. Thus, for each species, the genomes IDs of the genomes that were used for hyper-parameter tuning were saved by the pipeline, and the pipeline made sure that in other antibiotics these genomes did not fall in the hold-out set. In cases where the number of unobserved genomes was less than 10% of the total number of genomes, due to smaller number of genomes for some antibiotics compared to the antibiotic that was used for hyper-parameter-tuning, less than 10% of the data were used as the hold-out set.

In the hyper-parameter tuning stage, we performed cross-validation with five folds. In each fold, 20% of the data were used for validation and 80% were used for parameter tuning (see Figure 2). In each fold, the hyper-parameter combination that minimized the mean squared error of validation was selected as the optimal combination. After running the tests, we had five sets of hyper-parameters corresponding to five folds. The final chosen hyper-parameters were those that minimized the RMSE error on the validation set. Since some of the datasets are very large, more than one hundred gigabytes, it is not computationally feasible for many researchers to experiment with all possible combinations of all hyper-parameters in an exhaustive search manner. We used Optuna [74], a software package that implements early stopping for XGBoost by using the built-in validation check feature of XGBoost and a tree-structured Parzen estimator (TPE) [75] for choosing combinations of hyper-parameters to perform hyper-parameter tuning in each one of the mentioned five folds. The hyper-parameters which we searched were learning rate, maximum tree depth, minimum child weight, lambda, gamma, column sub-sampling, maximum delta step, and number of estimators. Table 1, shows the ranges of the hyper-parameters which were swept.

#### 2.5.2. Training the Model

After optimizing the parameters of XGBoost, it was used to train and test with 10 different feature extraction methods for *C. jejuni*, *S. enterica*, *N. gonorrhoeae* and *K. pneumoniae*. We used two schemes for train and test: 10-fold cross-validation and evaluation of the hold-out set (See Figure 2). For the 10-fold cross, in each fold, 10% of data were used for testing and 90% were used for training. In the training part, 10% of the training data were used for validation to prevent over-fitting and the rest was actually used for training the model. The trained model of each fold was later tested with the corresponding test set. In the hold-out evaluation, all of the data except for the hold-out set were used for training, and the hold-out set was used for final evaluation.

#### 2.5.3. Feature Selection

After training the model with cross-validation in 10 folds, we wanted to extract the important features. Important features serves two purposes:

- A concise model using only important features can be built.
- The features can be used to gain biological insights.

##### 2.5.3.1. Feature Selection Pipeline

To find the important features, we ranked them by their absolute SHAP [76,77] values. SHAP (shapley additive explanations) assigns each feature an importance value based on the contribution of that feature to the output of the model by comparing the output of the model with and without that feature [76]. A feature that makes a great change in the output will have a great absolute SHAP value and a feature that does not make a huge difference in the output will have a small SHAP value.

Since we performed 10-fold cross-validation for each species–antibiotic combination, there were 10 models in each experiment. However, we wanted a unified set of important features from all folds. To combine the features selected in different folds, we used the following method: In each fold, the top $N_{top}$ features are picked and added to the set of important features. Any feature in this set might be in the top $N_{top}$ features in the model in one or more fold. For each feature in the set of important features, the number of folds in which it makes it to the top $N_{top}$ is counted, and features are ranked based on this number. For example, a feature that makes it to the top $N_{top}$ in 9 folds out of 10, receives a higher rank compared to a feature that makes it to the top $N_{top}$ only 3 times. This method returns a list of features ranked based on their importance in all folds of cross-validation. In this pipeline $N_{top}$ is a hyper-parameter. We tuned this hyper-parameter and found the optimal value of 50 for it. Details of this optimization are provided in the rest of this section. Pseudo-code for the feature selection pipeline is presented in Algorithm 1.
**Algorithm 1** Feature selection. 1:**for**i=1,2,⋯,10**do** 2:    Rank the features in fold *i* based on their SHAP values; 3:    Selected features *i* = Top $N_{top}$ features in fold *i*; 4:**end for** 5:Find union of selected features in all folds; 6:**for** feature in union set **do** 7:    Find in how many folds this features makes it to top $N_{top}$; 8:**end for** 9:Rank features in the union based on the number of times each feature appears in the top $N_{top}$;10:Output the ranked list of features;

In the results section, for different antibiotic-species, we are reporting the top features selected by this pipeline. Moreover, for each feature, all of the existing strains (including the ones that are filtered out) are divided into 2 groups: positive strains, strains that have the feature at least once, and negative strains, strains that do not have the feature. The Kruskal–Wallis (K–W) test [78] is used to compare the MICs of two groups and see if there is a significant difference. The reported features are the ones that appear in the top 50 features in at least 8 folds out of 10 folds and their *p*-values are less that the significance threshold (0.05).

##### 2.5.3.2. Training the Model with the Selected Features

After obtaining the list of features sorted by their importance, we built models with the most important features. To do this, first, we picked only the most important features and trained the model in 10 fold cross-validation with only 1 feature. Then we selected the 2 most important features and trained the model in 10 folds. The same processes was repeated up to the 40 most important features. The minimum number of features that reached the maximum accuracy was selected as the required number of features for the “selected-feature” model. Then this selected-feature model was tested in the hold-out evaluation scheme.

##### 2.5.3.3. Tuning the Hyper-Parameter $N_{top}$

In Algorithm 1, $N_{top}$ is a hyper-parameter. We tested the values of [20, 30, 40, 50, 60, 70, 80] for this hyper parameter on a dataset of *C. jejuni* and tetracycline using gene content feature extraction. To find the best value for this hyper-parameter, we needed a metric to evaluate different values. Since the purpose of this pipeline was to find important features, the best performance was achieved by the set of features that resulted in good accuracy with the fewest features. The good accuracy that we choose as the benchmark was the average cross-validation accuracy when all of the features are used. In other words, the question was using which value of $N_{top}$ can a pipeline that is only using the selected features reach the average cross-validation accuracy of a pipeline that is using all features. The best performance was achieved by $N_{top}$ = 50, where the pipeline reached an accuracy better than the average accuracy of model trained with all features using only 10 features.

##### 2.5.3.4. Interpreting the Top Features

After selecting the top features, we wanted to know what these features are and what information can we extract from them, for two purposes:

- Validation: When the model finds top features that we already know are important AMR determinants, we know that the model has been trained and is working properly.
- Discovery of new AMR determinants: In an accurate model, the top features that are derived from the data can be used for discovering new AMR genes/SNPs.

For the gene content pipeline, we interpreted the functions of the selected genes by searching the gene ID of a representative gene of each cluster in the PATRIC database. In some cases we also blasted the important gene sequences in other databases such as CARD [27] and NCBI [53], as reported in Section 3.2. For SNP features, first we extracted the position of each SNP with respect to the reference, and then looked for the gene in which the SNP happened and then found the function of that gene in the gene bank file provided for the reference in NCBI database. For the *k*-mer features, we searched them in NCBI database using BLAST [79]. We specified the species name before each blast search. When a query is searched against a database, it might align with multiple positions in different genes. We only considered the hits with perfect matches to the query and discarded cases where only a part of the *k*-mer matched a subject. When there was a disagreement among perfect matches, meaning that the query aligned with multiple genes, we reported the gene that had the bigger number of hits. In cases where the hits with full query coverage were from different genes and none of these genes outnumbered the others, and where a product of the gene with a maximum number of hits was a hypothetical protein, we did not report anything.

### 2.6. Software Implementation and Availability

All of the computational results reported herein were performed using Python version 3.7.1, CentOS Linux release 7.6.1810 and Red Hat Enterprise Linux Server release 6.5. We have made all of the source code available on Github at https://github.com/TahaAslani/AAk-mer.

## 3. Results

The results section is divided into two subsections: First, we present results of performances of different techniques. Second, we analyze the AMR determinants for different antibiotics by interpreting the top features that our feature selection pipeline picked. Analyses of feature stability and performance evaluations for selected-feature models are provided in Appendices Appendix G and Appendix H, respectively.

### 3.1. Performances of Different Feature Extraction Methods

#### 3.1.1. Comparison of Required Numbers of Features

**Figure 3:**
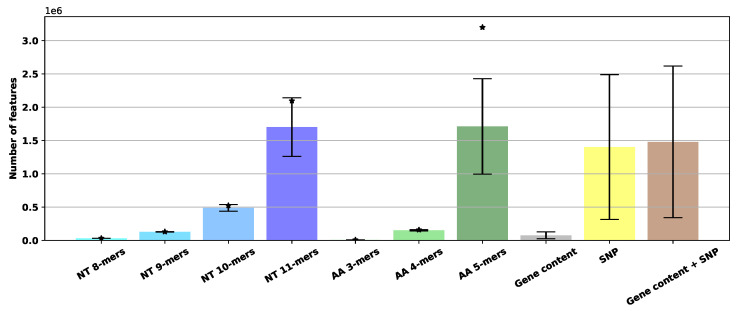
Average and standard deviation of number of features for each method across all species. An asterisk indicates the maximum theoretically possible number of *k*-mer features, where applicable.

When comparing different feature extraction methods, it is important to take the number of features into account, because it directly affects memory usage and computational complexity. For *k*-mer counting methods, the number of features is determined by the number of existing *k*-mers in all genomes. For SNPs, the number of features is the number of existing SNPs in all genomes multiplied by four, because of one-hot coding of four possible nucleotides after mutation. In case of gene content, the number of features is the number of existing clusters after clustering the genomes. Finally, for “gene content + SNP” the number of features is simply the addition of the number for gene content and SNP methods. Figure 3 depicts the average and standard deviation of number of features for each method across all species. For SNP data, although the number of features is large, the data are always sparse (more than 95% zero) so sparse data format can be used to mitigate memory issues.

#### 3.1.2. Comparison of Regression Packages

**Figure 4:**
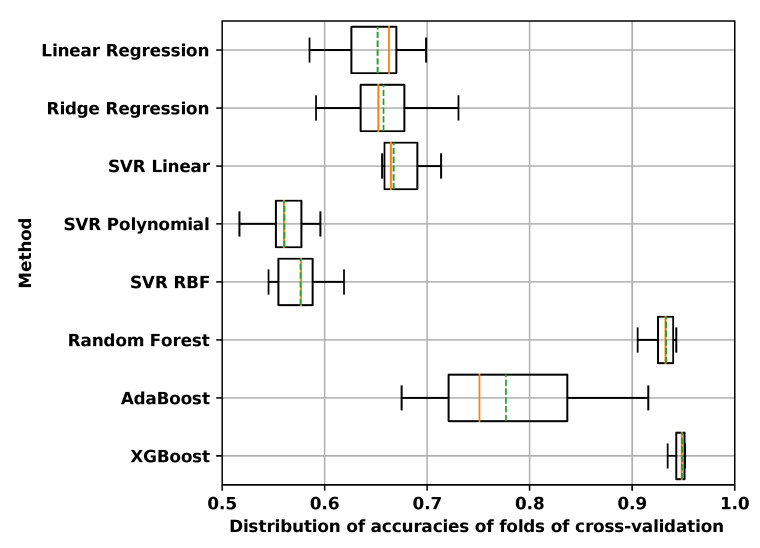
Comparison of ±1 two-fold dilution accuracy of regressors in 10 folds of cross-validation on predicting MIC of ampicillin for *Salmonella enterica* with 4-mers of amino acid. In each box plot, the whiskers represent the maximum and minimum. The boxes represent the first and the third quartiles. The orange line represents the median and the green dashed line represents the mean.

As mentioned in Section 2.5, different regression packages were compared based on predicting MIC of *S. enterica* and ampicillin from 4-mers of amino acid. We chose 4-mers of amino acid because the number of features and data size for this feature extraction method were not too large and we could test computationally expensive methods, like random forest, using this dataset. Figure 4 shows the results of this comparison.

The best performance, as measured by the accuracy of prediction of ±1 two-fold dilution level, was achieved by XGBoost. The second best option was random forest, which performed close to XGBoost in terms of accuracy; however, the computational complexity of XGBoost was significantly lower than that of random forest. XGBoost is thus more scalable to bigger datasets. XGBoost was also reported to outperform other machine learning packages for MIC prediction in [15]. Moreover, in [36] gradient boosting overall performed better than logistic regression, random forest and deep learning.

#### 3.1.3. Accuracy of Different Feature Extraction Methods Using XGBoost

**Figure 5:**
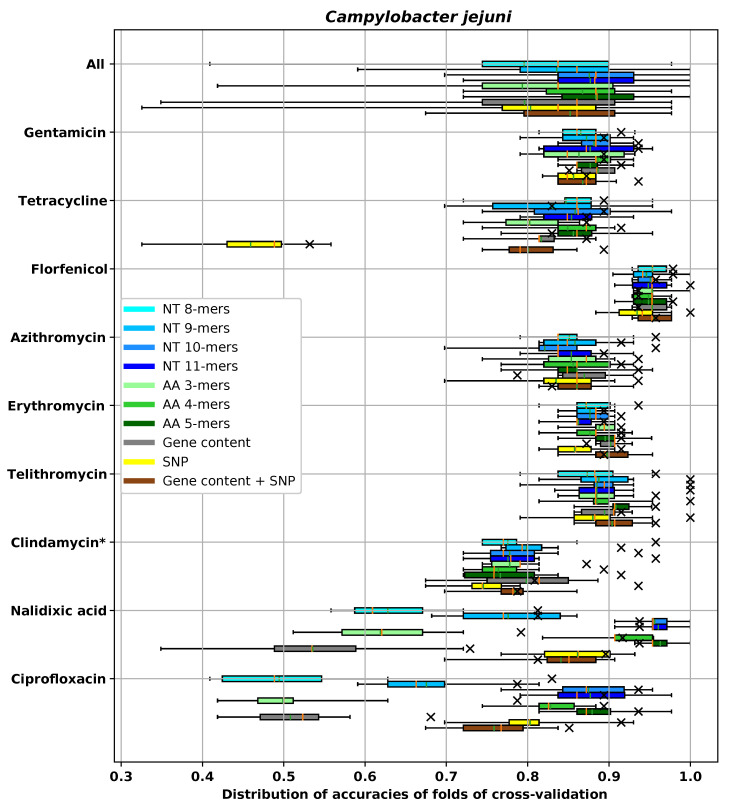
Distribution of ±1 two-fold dilution accuracies in different methods for *Campylobacter jejuni*. The box plots are similar to Figure 4. The orange line represents the median and the green line represents the mean. The × marks represent the accuracy of the hold-out set. The antibiotic used for hyper-parameter tuning is indicated by an asterisk. For each method, the top boxes, labeled as “All,” were obtained by combining all ten folds for all antibiotics.

**Figure 6:**
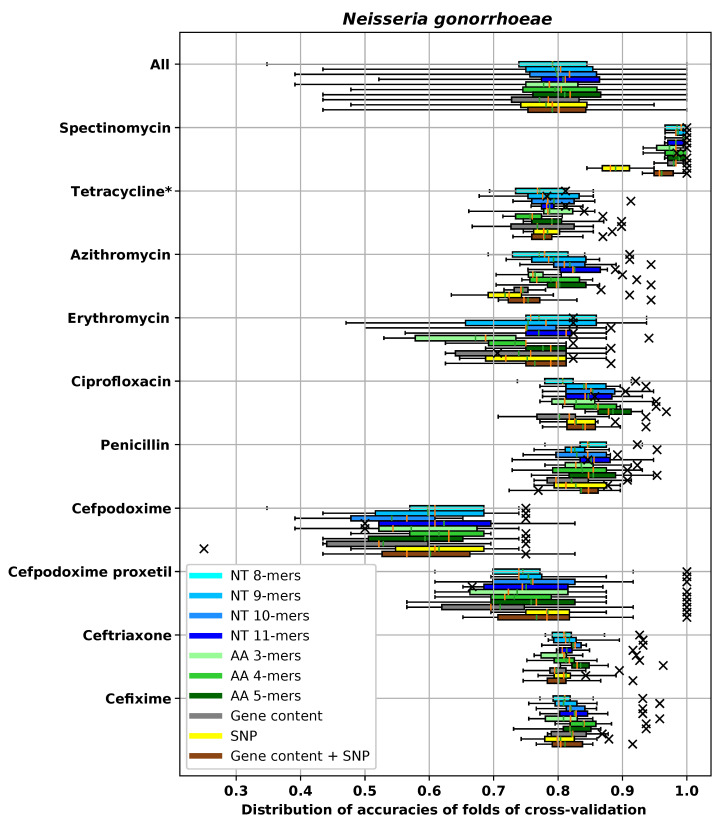
Distribution of ±1 two-fold dilution accuracies in different methods for *Neisseria gonorrhoeae*. Plots are similar to Figure 5.

**Figure 7:**
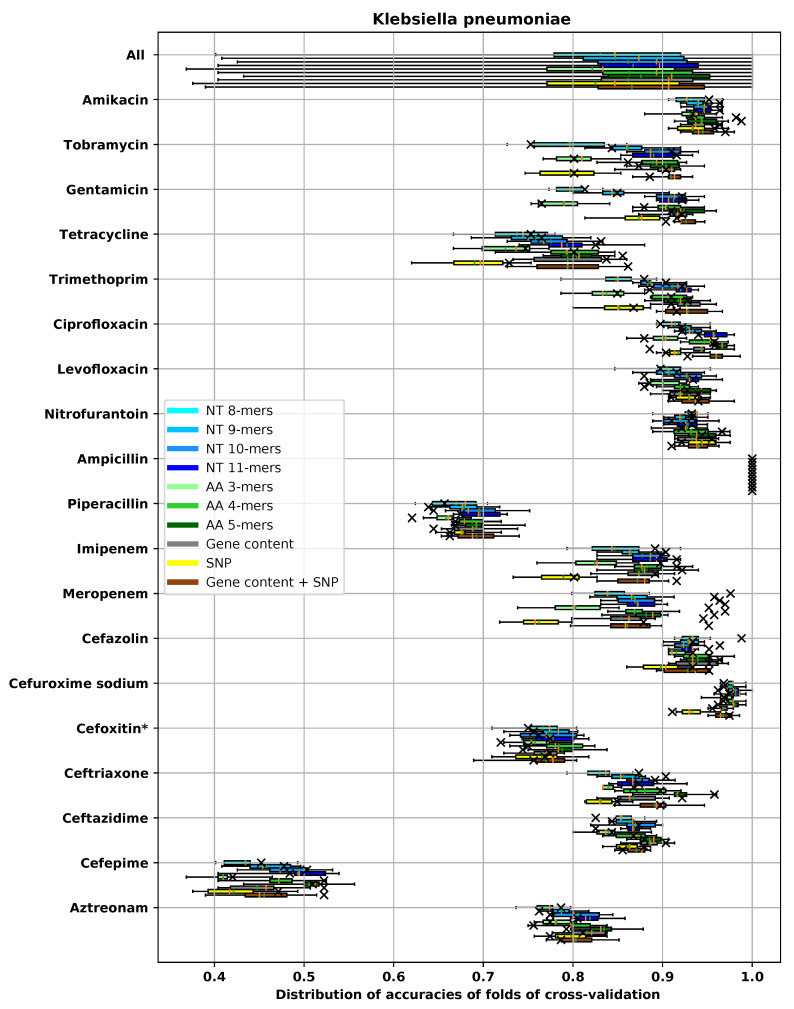
Distribution of ±1 two-fold dilution accuracies in different methods for *Klebsiella pneumoniae*. Plots are similar to Figure 5.

**Figure 8:**
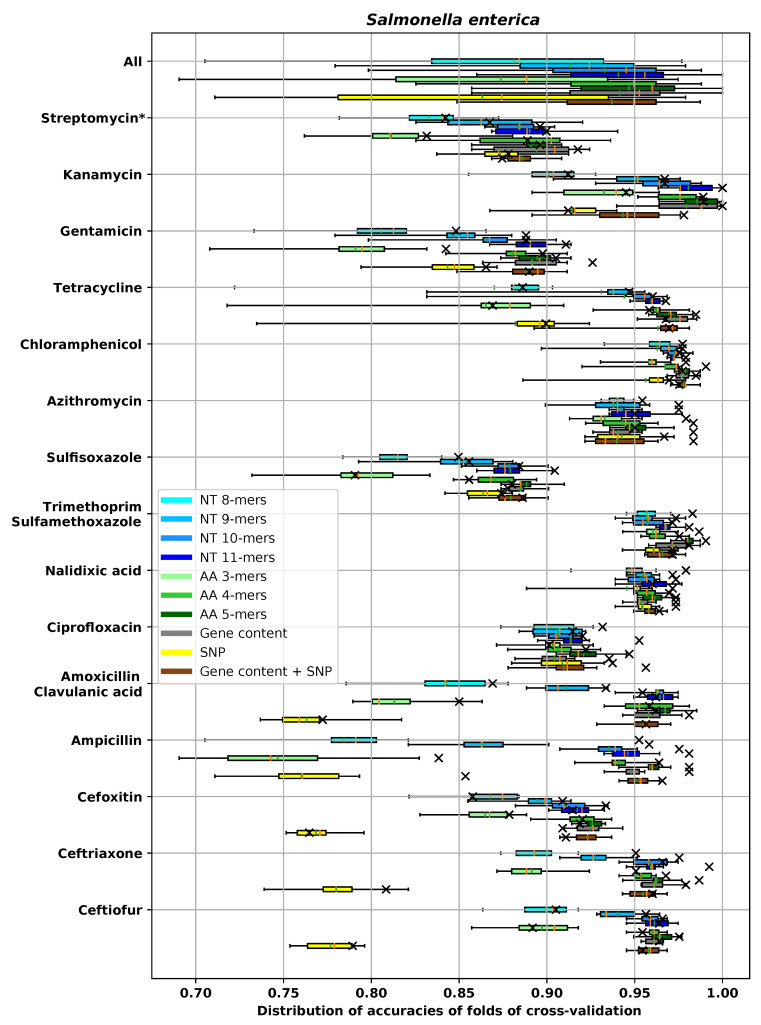
Distribution of ±1 two-fold dilution accuracies in different methods for *S. enterica*. Plots are similar to Figure 5.

For the dataset for each species and antibiotic combination, we tested and compared different feature extraction methods. In the main text, we used ±1 two-fold dilution to measure MIC prediction accuracy. A complete evaluation of all models using all metrics described in Section 2.4 is provided in Appendix E. Since we used cross-validation to measure the performance, in each experiment there were 10 different prediction accuracies corresponding to 10 different folds. The average and the box plot of distribution of the ±1 two-fold dilution accuracies are reported in Figure 5, Figure 6, Figure 7 and Figure 8 for different species. In these figures, the antibiotics are ordered based on their classes. In each box plot, the whiskers represents the maximum and minimum. The boxes represent the first and the third quartiles. The orange line represents the median and the green line represents the mean. The accuracy on the hold-out set is also represented by an “×” mark.

#### 3.1.4. Searching Top Amino Acid and Nucleotide *k*-mers in NCBI Database

In order to compare the interpretability of models trained with nucleotide and amino acid *k*-mers, for eight species–antibiotic combinations, we searched the top amino acid 5-mers and nucleotide 11-mers, chosen by the proposed feature selection pipeline in NCBI database. These datasets were: *K. pneumoniae*–tetracycline, *K. pneumoniae*–tobramycin, *K. pneumoniae*–imipenem, *S. enterica*–cefoxitin, *S. enterica*–amoxicillin clavulanic acid, *S. enterica*–ampicillin, *S. enterica*–chloramphenicol and *S. enterica*–sulfisoxazole. For both amino acid and nucleotide *k*-mers, we chose the longest *k*-mers size, which was determined by computational complexity limitations. As mentioned in Section 3.1.1, both of these methods have virtually the same feature size. The searched features were chosen based on these two criteria: They appear in the top 50 features at least in eight folds out of 10 folds and their K–W test *p*-value is less than the significance threshold (see Section 2.5.3). In all of the searches we used the default parameters of NCBI BLAST.

For the top amino acid 5-mers, the chosen features in all datasets aligned to genes that were known AMR determinants. Results of these alignments are presented in Table 2, Table A5, Table A6, Table A7, Table A8, Table A9, Table A10 and Table A11, respectively. On the contrary, in case of nucleotide 11-mers none of the queries hit a subject on NCBI database.

### 3.2. Analysis of AMR Determinants for Specific Antibiotics Families

In this subsection, we analyze the performances of models that were trained with different feature extraction methods for different families of antibiotics, and we interpret the models by analyzing the top features that they selected.

#### 3.2.1. Resistance to Tetracycline

**Figure A5:**
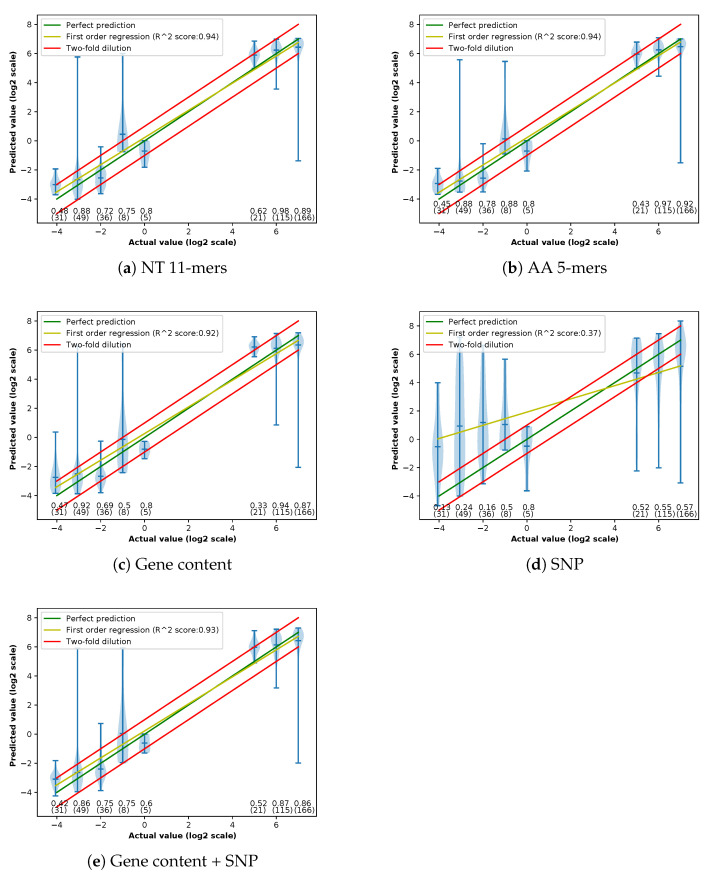
Violin plots of performances of different methods in predicting MIC of tetracycline for *C. jejuni*. X-axis is the actual value and y-axis is the predicted value. Each violin shows the kernel density estimation distribution of predictions for one MIC actual target value. Below each violin, the ±1 two-fold dilution accuracy of that target value is mentioned, and below that, the number of strains with that target value is mentioned in parentheses. The green line represents the perfect prediction and the yellow line represents the first order regression between the actual values and predicted values. The red lines represent the limits of the perfect predictions’s ±1 two-fold dilution. (**a**) NT 11-mers, (**b**) AA-5mers, (**c**) gene content, (**d**) SNP, (**e**) gene content + SNP.

In this section, we analyze determinants of resistance to tetracycline that the models found for different species. It is well-known that resistance of *C. jejuni* to tetracycline is conferred by the presence of tet(O) gene [80,81]. Tet(O) belongs to the class of ribosomal protection proteins that cause resistance by dislodging tetracycline from its primary binding site on the ribosome [81,82,83]. When XGBoost uses feature extraction methods that are capable of detecting the presence of this gene, such as amino acid 5-mers or gene content, it can detect AMR and predict MICs of organisms reasonably well. On the other hand, when SNPs are used as features, it cannot detect presence or absence of this gene. This can be seen in Figure 5, where results of using SNP features are not as good as other methods. Moreover, Figure A5 in Appendix C provides more details about prediction error for models trained with different methods. Our feature selection method found tet(O) for gene content and gene content + SNP. In amino acid 5-mer pipeline, the top feature, “RKAEY,” aligns with this gene. Out of total of 481 strains (including the ones that were filtered out), 327 strains had this gene and 154 strains did not have this gene. The average MIC of strains that had this gene was 33.30 (standard deviation 51.16) mg/L and the average MIC of strains without this gene was 13.11 (standard deviation 30.33) mg/L, K–W test [78] *p*-value: 0.0418.

The same pattern for accuracy of different methods can be seen in Figure 7 and Figure 8 for *K. pneumoniae* and *S. enterica*, respectively. For *K. pneumoniae*, the gene content and gene content + SNP pipelines found tet(A), tet(D), tetR. tet(A), tet(D) and TetR are known to confer resistance to tetracycline in *K. pneumoniae* [84,85]. We found that SNP features are not capable of reflecting the presence of those genes. Important features found by the amino acid 5-mers and gene content, using the method described in Section 2.5.3, for *K. pneumoniae* and tetracycline, are presented in Table A5 in Appendix D. The top op features of amino acid 5-mer pipeline are “DGLTT,” “LIMPV” and “HYGIL,” which align with TetR, Tet(A) and Tet(A), respectively.

##### Two Types of the tet(D) Gene

Gene clustering reveals two clusters associated with tet(D) gene, and two clusters associated with tetR gene. We investigated further to see why and determine whether both clusters are correlated with AMR. In the case of tet(D), two clusters are formed because, in the cluster represented by fig573.13783.peg.5353, the gene has been truncated to approximately one quarter of the length of the corresponding gene found in the cluster represented by fig573.14286.peg.4536. In fact, the gene sequence in most of the members in the cluster represented by fig573.13783.peg.5353 is the last 97 amino acid of the consensus gene sequence of the cluster represented by fig573.14286.peg.4536 (length of all of the sequences in this cluster is 394 amino acids). In the case of tetR, we found that this is due to the mutations in this genes in some strains, i.e., sequence identity of blasting the representative genes of two clusters is 52%.

Interestingly, strains that have the truncated version of the tet(D) have lower average MIC value compared to strains that do not have this gene at all. For 73 strains that have the truncated version of tet(D) gene, the average MIC is 5.92, with a standard deviation of 4.56, and the average MIC of the rest of the strains is 9.78 (standard deviation 6.17) (Kruskal–Wallis *p*-value of $2.54\times 10^{-6}$). When MICs of these strains are compared to CLSI breakpoints, 11 are resistant, 9 are intermediate and 53 are susceptible. Notably, although based on our finding this gene is conferring antimicrobial susceptibility, its function for all of the versions is labeled as “Tetracycline resistance” in PATRIC database, presumably based on sequence homology. The FASTA file and PATRIC ID of the truncated versions of the gene as well as the long versions are provided in Supplementary Material. By contrast, the long version of tet(D) confers resistance as expected: out of 250 strains with the long version, 243 are resistant, 3 are intermediate, and 4 are susceptible.

The gene content pipeline also found a class A β-lactamase gene as a factor for resistance to tetracycline. This shows that some strains are multi-drug resistant.

For *S. enterica*, tet(A) has been reported before to confer resistance [86]. For the gene content method, our feature selection pipeline found tet(A) [86], tet(D) [86] and tetR. For the amino acid 5-mer method, the feature selection pipeline selected “GLIMP” and “GPLLF” which align with tet(B) and “ALYWH,” which aligns with tetR.

For *N. gonorrhoeae*, tet(M) [87] is selected by the gene content pipeline. Top amino acid 5-mers, “LLISA” and “PVSTP” also align with tet(M).

#### 3.2.2. Resistance to Quinolone Antibiotics

In this subsection, we analyze the causes of resistance to quinolone antibiotics, which include nalidixic acid and ciprofloxacin. Quinolone antibiotics target two essential bacterial enzymes, DNA gyrase and DNA topoisomerase IV [88]. Quinolone resistance can be caused by single amino acid changes in gyrase [88]. Gyrase is composed of 2 GyrA and 2 GyrB subunits [89]. For *C. jejuni* and both ciprofloxacin and nalidixic acid, SNP and gene content + SNP pipelines recognize a mutation in position 959,966 with respect to the reference genome (conversion of the original nucleotide to A) as the most important feature. This nucleotide is in the gyrA gene. For nalidixic acid and *C. jejuni*, the mean MIC of strains that have this mutation (N = 87) is 119.26 mg/L (standard deviation 25.56) and for strains without (N = 394) it is 6.43 mg/L (standard deviation 13.91) for a K–W *p*-value of $9.88\times 10^{-62}$. The top three *k*-mers selected by the amino acid 5-mer method also hit this gene. For ciprofloxacin and *C. jejuni*, average the MIC of strains that have this mutation (N = 87) is 7.27 mg/L (standard deviation 7.06) and the average MIC of strains that do not have this mutation (N = 394) is 0.16 mg/L (standard deviation 0.80), for a K–W *p*-value of $2.29\times 10^{-53}$. Mutations in many other positions of gyrA gene were also found for both antibiotics as important features for *C. jejuni*. Both SNP and gene content + SNP methods also found mutations in gyrB as a less important features for both antibiotics and *C. jejuni*. For *C. jejuni* and ciprofloxacin, the amino acid 5-mer pipeline found “PHGDT,” “HGDTA,” “GDTAV” and “DTAVY” as the top four important features. These are parts of a longer sequence “PHGDTAVY,” which aligns with a gene labeled “Campylobacter jejuni gyrA conferring resistance to fluoroquinolones” in CARD database [27] (gene bank accession: AJY14066.1). The top four features of amino acid 5-mers pipeline for *C. jejuni* and nalidixic acid are exactly the same.

**Figure A6:**
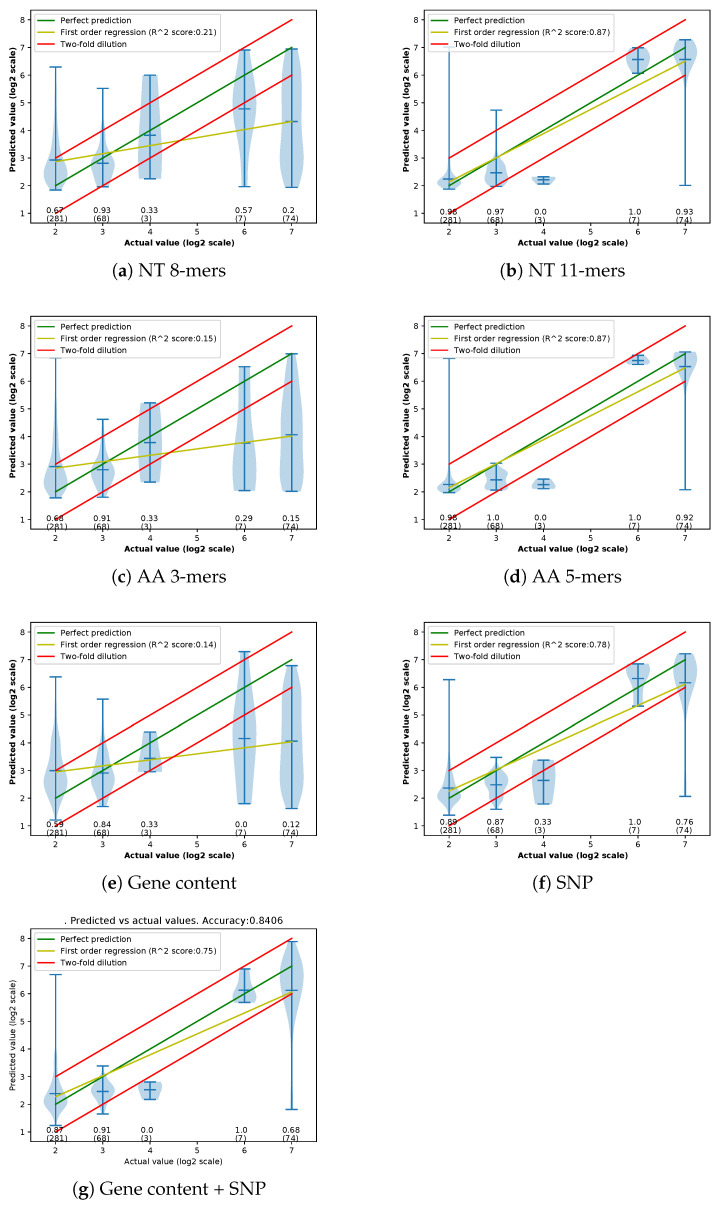
Violin plots of performances of different methods in predicting MIC of nalidixic acid for *C. jejuni*. X-axis is the actual value and y-axis is the predicted value. Each violin shows the kernel density estimation distribution of predictions for one target value. Below each violin, the ±1 two-fold dilution accuracy of that target value is mentioned, and below that, the number of strains with that target value is mentioned in parentheses. The green line represents the perfect prediction and the yellow line represents the first order regression between the actual values and predicted values. The red lines represent the limits of ±1 two-fold dilution. (**a**) NT 8-mers, (**b**) NT 11-mers, (**c**) AA-3mers, (**d**) AA-5mers, (**e**) gene content, (**f**) SNP, (**g**) gene content + SNP.

For these antibiotics, models which use SNP features perform better at predicting MIC than gene content, because the mutations in question occur at a finer resolution than simple gene presence/absence/count. Long amino acid and nucleotide *k*-mers are also capable of detecting these mutations, while shorter *k*-mers cannot distinguish these mutations from other genes with the same motif. In the case of a short *k*-mer size, short motifs are more likely to occur across the genome. The longer the *k*-mer, the more unique (i.e., only occurring once) it is likely to be; therefore, the interpretation based on the *k*-mer count will be less ambiguous. This is reflected in the sparsity of the feature matrix. For *C. jejuni* and nalidixic acid, sparsity (portion of matrix that is zero) of the feature matrix for amino acid 3-mers is 1%. For 4-mers and 5-mers of amino acids, the values are 22% and 58%, respectively. In the same manner, sparsity of the feature matrix for nucleotide 8-mers, 9-mers, 10 mers and 11-mers is 4%, 14%, 28% and 42%, respectively. From Figure A6, it can be seen that pipelines of nucleotide 8-mers, amino acid 3-mers and gene content perform worse at MIC prediction than longer *k*-mer sizes and SNP methods.

The same pattern was observed for performance of models trained with different feature extraction methods in [38] for classification of *Pseudomonas aeruginosa* strains as resistant or susceptible to ciprofloxacin: Methods that did not account for SNPs performed worse than methods that accounted for SNPs.

**Figure 9:**
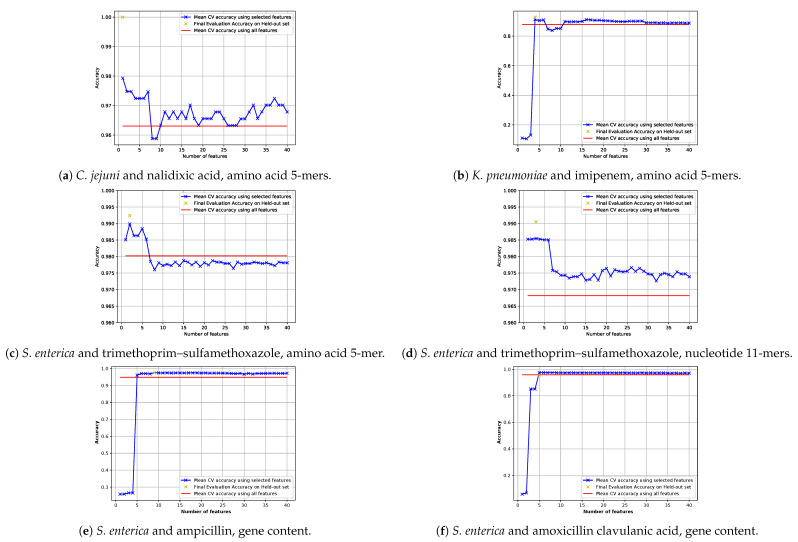
Change in average accuracy of cross-validation when different numbers of features are included in the selected-feature pipeline. (**a**): *C. jejuni* and nalidixic acid, amino acid 5-mers; (**b**): *K. pneumoniae* and imipenem, amino acid 5-mers; (**c**): *S. enterica* and trimethoprim–sulfamethoxazole, amino acid 5-mer; (**d**): *S. enterica* and trimethoprim–sulfamethoxazole, nucleotide 11-mers; (**e**): *S. enterica* and ampicillin, gene content; (**f**): *S. enterica* and amoxicillin clavulanic acid, gene content.

**Figure A7:**
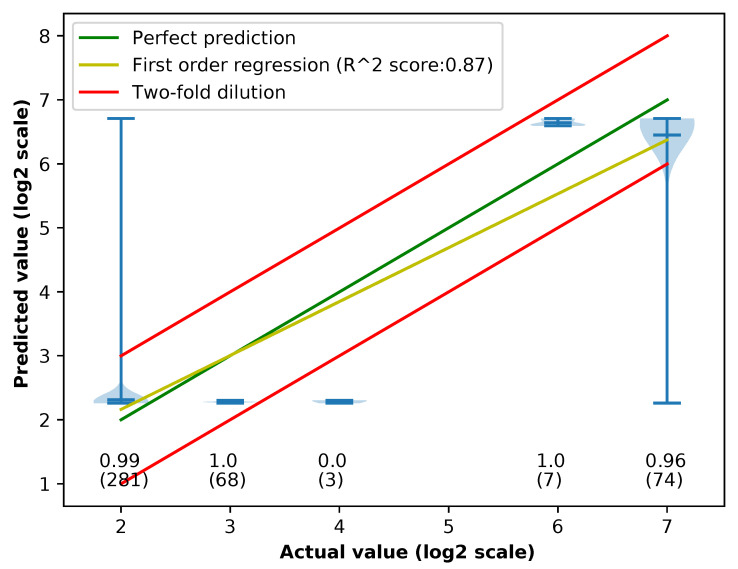
C. jejuni and nalidixic acid. Amino acid 5-mers. Violin plots of performance using only one 5-mer (“GDTAV”). X-axis is the actual value and y-axis is the predicted value. Each violin shows the kernel density estimation distribution of predictions for one target value. Below each violin, the ±1 two-fold dilution accuracy of that target value is mentioned, and below that, the number of strains with that target value is mentioned in parentheses. The green line represents the perfect prediction and the yellow line represents the first order regression between the actual values and predicted values. The red lines represent the limits of ±1 two-fold dilution.

In the case of *C. jejuni* and nalidixic acid, only one amino acid 5-mer is required to get the best accuracy. For this data, the selected-feature pipeline (described in Section 2.5.3.2) with amino acid 5-mers reached a very good accuracy using only the top feature (AA 5-mer: “GDTAV”), which is in “gyrase subunit A” (gene bank accession: AJY14066.1). In Figure 9a, the change of accuracy by increasing number of features is depicted. It can be seen that the prediction with more than one 5-mer feature only decreases accuracy. Additionally, in Figure A7 prediction performance via violin plot of the error using only one feature (“GDTAV”) is depicted.

#### 3.2.3. Resistance to Aminoglycoside Antibiotics

Aminoglycosides (AGs) are a class of antibiotics including tobramycin, gentamicin, amikacin, streptomycin and kanamycin, which bind to the bacterial ribosome and interfere with bacterial protein translation. Although in some rare cases mutations in the ribosomal target of AGs can contribute to resistance, the most widespread mechanism of resistance to these antibiotics is achieved by AG-modifying enzymes (AMEs) [90].

In Table A6, the most important genes discovered by amino acid 5-mers and gene content pipeline for tobramycin and *K. pneumoniae* are provided. The gene content pipeline selected ANT(2″)-la, AAC(6’)-Ib and ANT(3″)-la, which are all known AMEs [91] as important features. Amino acid 5-mer pipeline selected “PYEET” and “DASMV,” which align with AAC(3)-IIe; “YAQSY,” which aligns with AAC(6’)-Ib/AAC(6’)-II; and “DTTQV,” which aligns with ANT(2″)-Ia. All of these genes are known AMEs [91]. (“DTTQV” and “YAQSY” are not shown in Table A6). Models trained with SNP and short *k*-mers are not able to detect these genes. The same relatively poor accuracy of SNP can be seen for gentamicin and *K. pneumoniae*, Kanamycin and *S. enterica* and gentamicin and *S. enterica*.

In Table A6, the gene cluster that is represented by PATRIC ID fig573.12887.peg.5713 is labeled as a hypothetical protein in the PATRIC database, but when we BLAST the protein sequence against NCBI database, it matched a tunicamycin resistance protein in *Escherichia coli* (NCBI Reference Sequence: WP_110074664.1) with 100% coverage and 100% identity. This shows that the same gene is correlated with resistance for *K. pneumoniae* and tobramycin.

Both gene content and amino acid 5-mer pipelines also found the OXA-1, β-lactamase gene (see Section 3.2.4), which is known for conferring resistance to β-lactam antibiotics in *K. pneumoniae* [92]. In the case of the amino acid 5-mer pipeline, this gene was found via 5-mer “QFLRK.” Here, both pipelines selected it as an important factor for tobramycin, which means that there are multi-drug resistant strains in the data.

#### 3.2.4. Resistance to β-Lactam Antibiotics

β-lactam antibiotics are among the most commonly prescribed antibiotics and each have a 3-carbon and 1-nitrogen ring (β-lactam ring) [93]. The most important factor for resistance to these antibiotics is production of beta-lactamases [93]. Other mechanisms of resistance are decreased penetration to the target site, alteration of target site penicillin-binding proteins (PBPs) and efflux from the periplasmic space through specific pumping mechanisms [93].

##### Carbapenem Antibiotics

It can be seen in Figure 7 that models trained with SNP features alone are not capable of providing a good prediction for resistance of *K. pneumoniae* to imipenem and meropenem. Both of these antibiotics belong to a class of β-lactam antibiotics called carbapenem [94]. According to [95], the most common resistance mechanism of *K. pneumoniae* to carbapenem antibiotics is production of enzymes with carbapenemase activity that hydrolyze β-lactam antibiotics. SNP features are not a very effective feature extraction method for finding these genes. Gene content and long *k*-mers pipelines, on the other hand, can find presence of these genes easily.

For imipenem, a class A β-lactamase genes was selected as important feature by the gene content pipeline. The average MIC of strains that have this gene (N = 488) is 12.15 mg/L (standard deviation 5.37) and the average MIC of strains that do not have this gene (N = 1178) is 1.29 mg/L, (standard deviation 2.21), for K–W *p*-value of $1.56\times 10^{-236}$ (see Table A7). The amino acid 5-mer pipeline found “TCGVY” as the fourth important feature (not shown in Table A7). This 5-mer aligns with “carbapenem-hydrolyzing class A beta-lactamas” on NCBI database. Interestingly, as soon as this feature is included in the selected-feature pipeline (described in Section 2.5.3.2), the accuracy increases significantly (see Figure 9b). Amino acid 5-mers “TCGVY” and “WELE” also align with classA beta-lactamase genes.

##### Cephalosporin Antibiotics

Cephalosporins are a class of β-lactam antibiotics that includes cefoxitin, ceftiofur, ceftriaxone, cefazolin, cefepime, cefpodoxime, cefixime, ceftazidime and cefuroxime [96]. Like other β-lactam antibiotics, cephalosporins are inactivated by the β-lactamases produced by the bacteria [97]. Table A8 shows the features that amino acid 5-mer and gene content pipeline find for *S. enterica* and cefoxitin. Based on this table, the found “class C β-lactamases” gene is making a significant difference in the MIC value. Amino acid 5-mer pipeline found “ANKSY” as the most important 5-mer, which aligns with the same gene. The model trained with SNP features is not capable of detecting such genes, and this is why for cefoxitin, ceftiofur and ceftriaxone, SNP features perform relatively poorly compared to other methods (see Figure 8).

##### Other β-Lactam Antibiotics

The same pattern can be observed for other β-lactam antibiotics (ampicillin and amoxicillin clavulanic acid) and *S. enterica* in Figure 8: models trained with SNPs alone do not perform as good as models trained with long *k*-mers and models trained with gene content. Important genes found by amino acid 5-mer and gene content pipelines for *S. enterica*–ampicillin and *S. enterica*–amoxicillin clavulanic acid are provided in Table 2 and Table A9, respectively. It can be seen that for both antibiotics presence of β-lactamase genes is increasing MIC significantly. SNPs are not the best feature extraction method for detecting presence of these genes.

Figure 9e,f depicts results of gene content pipelines trained and tested with few selected features on the datasets of ampicillin and amoxicillin clavulanic acid, respectively. For *S. enterica* and amoxicillin clavulanic acid, the accuracy increases significantly when the third and the fifth features are included (Figure 9e). These genes are “class C beta-lactamase (EC 3.5.2.6) => CMY/CMY-2/CFE/LAT family” and “class A beta-lactamase (EC 3.5.2.6) => TEM family” respectively. For *S. enterica* and ampicillin, the first two features are class A beta-lactamase genes; however, it is only after inclusion of the fifth feature that the accuracy increases (Figure 9f). This gene is “lipocalin Blc.” Presence or absence of this gene alone does not make a significant difference in MIC; however, it is involved in the dissemination of antibiotic resistance genes [98] and that is why it plays an important role in MIC prediction.

For *S. enterica* and amoxicillin clavulanic acid, the amino acid 5-mer pipeline found “TDFLR,” “AHTWI,” “VIDMA,” “QNEQK” and “ASWVH” as important features that align with β-lactamase genes (see Table 2). Another important 5-mer is “NTAAN,” which aligns with “type IV conjugative transfer system coupling protein TraD.” Conjugative plasmids harboring antibiotic resistance genes can be transferred from one bacterium to another through physical contact. After conjugation, the recipient bacterium harbors the antibiotic resistance genes and transfers the acquired plasmid to other bacteria [99]. The amino acid 5-mer pipeline also selects “NQNYG,” that aligns with “cysteine synthase family protein.” It has been reported that cysteine synthesis is associated with antibiotic resistance of swarming *S. enterica* cells [100,101]. Another important 5-mer is “YWDYN,” which aligns with TolC family protein. TolC is required for the function of several drug efflux systems in *S. enterica* serovar Typhimurium [102]. The gene content pipeline found the gene whose product is tetracycline resistance regulatory protein TetR as an important feature for resistance to amoxicillin clavulanic acid. This shows that the strains that have this gene are probably multi-drug resistant.

For *S. enterica* and ampicillin, top amino acid 5-mers, “WMRDD,” “TDFLR,” “ASWVH” and “VIYQG” align with β-lactamase genes. “AMSQN” aligns with “response regulator transcription factor,” which mediates a cell’s response to changes in its environment [103]. These proteins can control the expression of genes that mediate antibiotic resistance as response to environmental signals [103]. The pipeline also found “NTAAN,” which aligns with conjugative transfer system coupling, and “YWDYN” aligns with TolC.

#### 3.2.5. Resistance to Chloramphenicol

Chloramphenicol is a broad-spectrum antibiotic that inhibits bacterial growth by stopping the protein synthesis [104]. Important genes found for *S. enterica* and chloramphenicol by amino acid 5-mer gene content pipelines are presented in Table A10. Top 5-mers, “WAYTL,” “YMVML” and “MDIYL” align with “chloramphenicol/florfenicol efflux MFS transporter FloR,” which is known to mediate resistance to chloramphenicol [105]. “TAWPV” aligns with “CmlA family chloramphenicol efflux MFS transporter,” which confers non-enzymatic chloramphenicol resistance [106]; “CDGFH” and “PVFTM” align with “type A chloramphenicol O-acetyltransferase,” which is a type of chloramphenicol acetyltransferase (CAT) gene that confers enzymatic chloramphenicol resistance to chloramphenicol [105,107,108,109]. “FAKFF” aligns with a Type IV secretion system protein, which can have an antibiotic resistance function [110]. For the gene content pipeline, efflux genes appear in the top genes. The pipeline found chloramphenicol acetyltransferases, which is known to cause resistance to chloramphenicol [108,109]. The model also found LysR transcriptional regulator. The role of this gene in regulation of antibiotic resistance for *Aeromonas hydrophila* was investigated in [111]. TetR resistance gene was found by the model as an important feature. This again shows multi-drug resistance. The importance of all of these genes is causing the SNP method to perform relatively poorly compared to other methods.

#### 3.2.6. Resistance to Sulfonamide

Sulfonamide antibiotics are broad-spectrum antibiotics, including sulfisoxazole and trimethoprim–sulfamethoxazole [112]. These antibiotics interfere with the synthesis of folic acid [112]. Table A11 presented important genes found for *S. enterica* and sulfisoxazole. Amino acid 5-mer pipeline found 5-mers “LDPGM,” “DPGMG,” “GMGFF” and “MGFFL” that all align with “sulfonamide-resistant dihydropteroate synthase Sul1.” In fact, these 5-mers are all different sections of sequence “LDPGMGFFL.” “LDPGM” and “GMGFF” are among the top 50 features in all 10 folds, but “MGFFL” and “DPGMG” only make it to the top features in seven folds (possibly because the model does not need them since they are correlated with the other two important features), and that is why they are not represented in Table A11. The gene content pipeline found two gene clusters represented by sulfonamide resistance proteins as well as ANT(3″)-Ia, which is an AG-resistance gene, discussed in Section 3.2.3 and RmuC, which has been reported to be involved in the resistance against norfloxacin [113].

For trimethoprim–sulfamethoxazole and *S. enterica*, the most important gene that the gene content pipeline found is “dihydrofolate reductase,” which is known for conferring resistance to trimethoprim in *Salmonella typhimurium* [114]. In Figure 9c,d, the accuracy of the selected-feature (i.e., reduced feature number) pipeline is depicted for different numbers of feature for amino acid 5-mers and nucleotide 11-mers, respectively. Interestingly, the amino acid 5-mer pipeline can predict MIC with 99% accuracy, using only two 5-mers: “IPWKI” and “TYNQW.” Both of these amino acid 5-mers align with the same gene using NCBI BLAST, when the organisms is specified: For “IPWKI,” 48 out of the top 50 matches, and for “TYNQW,” 47 out of the top 50 matches. For the same data, nucleotide 11-mers also reach very good accuracy using selected-features. In that case the best accuracy is obtained when the top three 11-mers are used. These three nucleotide 11-mers are “TATTAGGACCA,” “ATATTAGGACC” and “CCCAATAGGAA.” No significant similarity was found by NCBI BLAST when these nucleotide 11-mers were searched because of the short query length. Fortunately, in this particular case, the first and second 11-mers belong to the same region shifted by one position. This made us able to extract a longer 12-mer “ATATTAGGACCA” by combining the two. After the extending the length, NCBI BLAST found significant matches for the query but was not able to identify the gene because the pattern was similar to many other positions in the genome. There was no hit for a “dihydrofolate reductase” gene in the top 50. Seven matches to the target gene were found when the top 100 matches were considered. This example shows the superiority of amino acid *k*-mers compared to nucleotide *k*-mers.

#### 3.2.7. Resistance to Macrolide Antibiotics

Macrolide antibiotics are composed of more than two amino or neutral sugars attached to a lactone ring [115]. These antibiotics include azithromycin and erythromycin [115].

For *N. gonorrhoeae* and erythromycin, the top selected feature by amino acid 5-mer pipeline is “SKSET,” which aligns with two-component sensor histidine kinase. A two-component system (TCS) is a mechanism in bacteria for receiving an external signal, for example, the presence of antibiotic, and a response regulator that conveys a proper change in the bacterial cell physiology [116]. Expression of antibiotic resistance determinants may be regulated by some TCSs [116]. Another important 5-mer is “SINRE,” which aligns with pilus assembly/adherence protein PilC. Mutation in this gene in *Pseudomonas aeruginosa* has been reported to cause resistance to aminoglycosides [117]. The gene content pipeline found “RND efflux system” as an important feature, which is a widespread resistance mechanism [118].

## 4. Discussion

When considering the choice of feature extraction method for machine learning AMR prediction, our results demonstrate that different considerations support the use of alternative methods. In particular, the optimal choice of feature extraction method depends on the importance of necessity of gene assembly, quantitative AMR prediction accuracy, constraints on computational complexity (i.e., speed and memory) and the ability to interpret the model to yield biological insight. Table 3 summarizes the comparison of different methods based on these issues. We discuss the comparative performance in the context of the aforementioned practical issues of feature extraction method in turn below.

### 4.1. Requirement of Gene Assembly

Among the feature extraction methods we present here, nucleotide *k*-mers and SNPs do not require the assembly of genes. The input to these methods can be nucleotide contigs obtained by assembling short reads together, possibly using de novo assembly. In the case of SNP features, full assembly of the genome is required only for the reference because each SNP must have a unique position with respect to the reference genome. The requirement of assembly just for the reference genome is usually not an issue because the reference can be obtained from databases such as NCBI [53].

For amino acid *k*-mers and gene content (and consequently gene content + SNP) methods, by contrast, the input to the feature extraction method is the amino acid sequence of the genes. This means that just assembling the short reads to contigs is not enough and gene finding must be performed to find the regions of the sequence that encode the genes and these regions must translated to amino acid sequences. This adds an extra pre-processing step to these methods.

Not requiring gene assembly makes nucleotide *k*-mers an interesting option for clinical AMR prediction, where next generation sequencing technologies can be used for predicting AMR in real-time without using the culture-based methods [119,120]. In that situation, predicting AMR as fast as possible and as cheaply as possible is the top priority. Thus, the best option will be a model that utilizes selected-features (i.e., a reduced model based on the top features) that also does not require gene assembly to generate features. For example, DNA microarrays can be employed to rapidly identify presence or absence of certain nucleotide *k*-mers (selected features). On the other hand, in a scenario where researchers want to train and interpret a model to learn more about AMR mechanisms and possibly discover new mechanisms, assembly of the genes is not an issue. Thus, amino acid *k*-mers are the better option because of the lower feature size and better interpretability of this method.

### 4.2. Predicting AMr Accurately

In Table 4, performance rankings of each method based on the average ±1 two-fold dilution accuracy for all antibiotics combined are presented for different species. Amino acid 5-mers always get the best performance. Then nucleotide 11-mers are second, except for *S. enterica*, where gene content is the second best.

Generally, gene content features and SNP features, on their own when used separately, are unable to capture all of the AMR determinants in all datasets. As in some cases genes cause resistance and in other cases SNPs cause resistance. This was discussed in detail in Section 3.2. On the other hand, *k*-mer counting methods can capture AMR phenotype from both genes and SNPs. If a certain gene is causing AMR, *k*-mer features of that gene will only show in the resistant genomes and if a certain SNP is causing AMR, *k*-mers can capture the changed nucleotide pattern. However, for both of these tasks, the *k*-mer length must be long enough to be able to distinguish between the genomes that have these patterns and those that don’t. Using long *k*-mers is hard because the number of features increases exponentially with *k* (at least before a the saturation point limited by the entire species’ *k*-mer vocabulary). However, we have shown that for amino acid *k*-mers this increase in feature size is less severe than nucleotide *k*-mers. Moreover, amino acid *k*-mers achieve better performance in terms of average ±1 two-fold dilution accuracy.

### 4.3. Comparison of Required Number of Features

In machine learning, an excessive number of features can increase the required memory and lead to over-fitting [121]. We have shown that longer *k*-mers reach better accuracies since they are more likely to capture a certain gene or SNP that is causing AMR. However, after increasing the *k*-mer length, the dataset becomes too large to handle by the machine learning algorithm. One advantage of amino acid *k*-mers over nucleotide *k*-mers is that it is a more compact representation of the biological information: each codon consists of three nucleotides and translates into one amino acid. Since the alphabet size of a nucleotide is four, there are $4^{3}$ nucleotide distinct codons but they translate to 20 amino acids. The biological redundancy in the nucleotide alphabet compared to that of amino acids can be exploited to decrease the feature size and have a more compact representation of the data, when amino acid *k*-mers are employed instead of nucleotide *k*-mers. A nucleotide *k*-mer of length *k* is equivalent to an amino acid *k*-mer of length k/3. For the case of canonical nucleotide *k*-mers, the ratio of the maximum number of nucleotide *k*-mers to the maximum number of equivalent amino acid *k*-mers is as follows (proof of provided in Appendix B):

(3) $$\left\{\begin{array}{cc} \frac{1}{2}\frac{4^{k}}{20^{k/3}}\approx \frac{1}{2}1.4736^{k} & \;\mathrm{if}\;k\;\mathrm{is}\;\mathrm{odd}\\ \frac{1}{2}\frac{4^{k}+2^{k}}{20^{k/3}}\approx \frac{1}{2}\left(1.4736^{k}+0.7368^{k}\right) & \;\mathrm{if}\;k\;\mathrm{is}\;\mathrm{even} \end{array}\right.$$

This can be seen in Figure 3, where the number of amino acid 5-mers (equivalent to nucleotide 15-mers) is approximately equal to nucleotide 11-mers. This is why Nguyen et al. had to break the total number of strains into different parts and train a separate model for each part of the data to be able train XGBoost with 15-mers of amino acid for *S. enterica* [24]. On the other hand, as we have shown here, 5-mers of amino acid leads to a reasonable number of features. The number of features for the gene content method is usually low compared to *k*-mer counting methods with long *k*-mers (see Figure 3). For the SNP feature extraction method and combined gene content + SNP features, the number of features is large for large datasets, like *S. enterica*. Required resources for dataset of one antibiotic for each species are provided in Appendix I.

### 4.4. Interpretability of the Model

When the AMR prediction model is trained without any a priori knowledge, it does not have any initial bias and learns the entire mechanism from the data. For such a model, a desired characteristic is interpretability. Here, we call a model “interpretable” if it has the following properties:

- Validation: mechanisms predicted to be important in an interpretable model can be compared to previously known AMR mechanisms, so that we know the model is working properly.
- Discovery: predictions of an interpretable model can be used to discover new AMR mechanisms that have not been discovered before.

Unlike nucleotide *k*-mers, amino acid *k*-mers are easy to interpret. We interpreted the models by analyzing the top features, selected by our proposed feature selection pipeline. The interpretation of the models was performed in Section 3.2. For the gene content method, the important features can be interpreted by finding the gene selected by the pipeline in databases, such as PATRIC [26], NCBI [53] or CARD [27]. For SNP features the same process can be performed by finding the genes in which the SNP has happened, and finding the position of the mutation and interpreting the gene and the mutation. Thus, gene content and the SNP model are both easily interpretable, because the genes always align uniquely to the corresponding subject using a method like BLAST. For the *k*-mers features, interpreting the model can be achieved by blasting the important *k*-mers against databases, such as NCBI [53] using BLAST [79] to see where the *k*-mers align. If *k*-mers do not align or align with multiple genes with different functions, we can claim that the model is not easily interpretable. In case of nucleotide *k*-mers, although 10-mers and 11-mers are able to have good accuracy in MIC prediction, in many cases the important 11-mers are not long enough align with the right position when searched with tools like BLAST. This was analyzed in Section 3.1.4. For instance, Nguyen et al. noted that they increased the *k*-mer length to 15 nucleotides to make the features identifiable using BLAST. This increased the computational complexity of their analysis significantly [24]. On the other hand, amino acid 5-mers can be easily searched using BLAST, and they align unambiguously to the target genes. Thus, for amino acid *k*-mers, interpreting the top features using BLAST is easier compared to nucleotide features.

## 5. Conclusions

In this paper, we proposed a new feature extraction method, amino acid *k*-mers, for predicting MIC of an antibiotic on a bacterium from its genome and compared this method to different existing methods, namely, nucleotide *k*-mers, gene content, SNP and gene content + SNP. We applied all of the methods to MIC data of *C. jejuni*, *S. enterica*, *N. gonorrhoeae* and *K. pneumoniae*, finding that amino acid *k*-mers provide comparable or superior performance across multiple metrics, in particular accuracy, interpretability and computational complexity.

Notably, amino acid and nucleotide *k*-mers have different applications. *k*-mer counting methods are robust in predicting AMR, particularly because they can capture absence or presence of a unique gene and SNP. However, these methods are only effective when the *k*-mer length is long enough, which can make the process of training computationally expensive because long *k*-mer length leads to a large feature size. We have shown that amino acid *k*-mers are less prone to the problem of large feature size compared to nucleotide *k*-mers. Moreover, amino acid *k*-mers are easier to interpret, because their top features are more likely to be uniquely identified using search algorithms such as BLAST. The main drawback of amino acid *k*-mers is that they require assembly of the genes. In a situation where assembly of the genes is not a problem, we recommend amino acid *k*-mers. An example of this situation is when researchers want to train a model to predict AMR in dataset of sequenced genomes and learn more about AMR mechanisms by interpreting the model. On the other hand, in a situation where assembling the genes is not possible, and the goal is to predict AMR and interpreting the model is not the top priority; or enough resources are available and having a large enough number of features is not an issue, nucleotide *k*-mers are an alternative option. An example of the latter scenario is predicting AMR in a medical clinic. In such a scenario, we propose utilization of a feature selection pipeline in which the most important features are selected and used to build models with a small number of features. Such selected-feature models, in many cases, achieve better accuracy compared to a model that uses all features. The selected-feature model can be useful in the clinical resistance test applications, where resistance to an antibiotic must be tested in real time and at minimum cost.

## Figures and Tables

**Table 1:** Examined values of hyper-parameters.

Hyper-Parameter	Ranges	Sampling
Learning rate	[$10^{-5}$, 0.5]	Log uniform
Maximum tree depth	[3, 10]	Uniform integer
Minimum child weight	[2, 8]	Uniform
Lambda	[0, 10]	Uniform
Gamma	[0, 3]	Uniform
Column sub-sampling	[0.25, 1]	Uniform
Maximum delta step	[0, 10]	Uniform
Number of estimators	[50, 100]	Uniform integer

**Table 2:** Important genes found for *S. enterica* and amoxicillin clavulanic acid by amino acid 5-mer and gene content methods.

Amino Acid 5-mers					Gene Content			
**5-mer**	**Gene Product**	**Number of +/− Strains**	**Mean (STD) MIC of +/− Strains**	***p*** **-Value**	**Gene Product**	**Number of +/− Strains**	**Mean (STD) MIC of +/− Strains**	***p*** **-Value**
TDFLR	class A beta-lactamase	143/5135	12.7 (12.7)/11.1 (20.8)	$1.5\times 10^{-17}$	Class A beta-lactamase (EC 3.5.2.6) => CARB/PSE family, carbenicillin-hydrolyzing	42/5236	15.3 (2.8)/11.1 (20.7)	$7.7\times 10^{-16}$
AWLWQ		49/5229	12.2 (9.7)/11.1 (20.7)	$1.2\times 10^{-10}$	Class A beta-lactamase (EC 3.5.2.6) => HER family	56/5222	15.2 (10.5)/11.1 (20.7)	$3.9\times 10^{-19}$
NTAAN	type IV conjugative transfer system coupling protein TraD	868/4410	16.6 (15.8)/10.0 (21.3)	$8\times 10^{-309}$	Class C beta-lactamase (EC 3.5.2.6) => CMY/CMY-2/CFE/LAT family	736/4542	58.6 (13.0)/3.4 (6.5)	0.0
NQNYG	cysteine synthase family protein	42/5236	15.3 (2.8)/11.1 (20.7)	$7.7\times 10^{-16}$	Class A beta-lactamase (EC 3.5.2.6) => TEM family	815/4463	16.8 (16.2)/10.1 (21.1)	$1.6\times 10^{-289}$
YWDYN	TolC family protein	42/5236	15.3 (2.8)/11.1 (20.7)	$7.7\times 10^{-16}$	Transposase, IS3/IS911 family	14/5264	16.2 (14.6)/11.1 (20.6)	0.0007
PLKAD		745/4533	58.2 (13.5)/3.4 (6.3)	0.0	Tetracycline resistance regulatory protein TetR	38/5240	15.2 (2.9)/11.1 (20.7)	$2.5\times 10^{-14}$
AHTWI	CMY-2 family class C beta-lactamase	743/4535	58.3 (13.3)/3.4 (6.3)	0.0	Mobile element protein	294/4984	17.3 (17.0)/10.7 (20.7)	$3.3\times 10^{-97}$
QHFRV	pilin outer membrane usher protein SafC	812/4466	16.4 (15.7)/10.2 (21.2)	$1.8\times 10^{-284}$	DNA translocase FtsK	6/5272	33.7 (30.4)/11.1 (20.6)	0.043
VIDMA	CMY-2 family class C beta-lactamase	872/4406	51.4 (21.2)/3.1 (6.0)	0.0	ABC transporter involved in cytochrome c biogenesis, ATPase component CcmA	4/5274	56.0 (13.9)/11.1 (20.6)	0.0006
QNEQK	CMY-2 family class C beta-lactamase	744/4534	58.3 (13.4)/3.4 (6.3)	0.0				
ASWVH	CMY-2 family class C beta-lactamase	745/4533	58.3 (13.4)/3.4 (6.2)	0.0				
WQEVF		5/5273	39.0 (30.6)/11.1 (20.6)	0.016				
TIPPD	ParB/ RepB/ Spo0J family partition	59/5219	14.6 (10.6)/11.1 (20.7)	$4.9\times 10^{-18}$				

**Table 3:** A summary of the comparison of different methods.

	NT *k*-mers	AA *k*-mers	Gene Content	SNP	Gene Content + SNP
**Advantages**					
Ability to capture AMr determinants	Yes	Yes	Only for some datasets	Only for some datasets	Yes
Ability to interpret the model by uniquely finding product of the top features	Only for very long *k*-mers that possibly require a large memory	Yes	Yes	Yes	Yes
**Requirements**					
Assembly of the genes	Not required	Required	Required	Not required	Required

**Table 4:** Rankings of average accuracy among different methods for each species.

	*C. jejuni*	*N. gonorrhoeae*	*K. pneumoniae*	*S. enterica*
1	AA 5-mers	AA 5-mers	AA 5-mers	AA 5-mers
2	NT 11-mers	NT 11-mers	NT 11-mers	Gene content
3	NT 10-mers	NT 10-mers	Gene content + SNP	NT 11-mers
4	AA 4-mers	NT 9-mers	AA 4-mers	Gene content + SNP
5	Gene content + SNP	AA 4-mers	Gene content	AA 4-mers
6	NT 9-mers	NT 8-mers	NT 10-mers	NT 10-mers
7	SNP	Gene content + SNP	NT 9-mers	NT 9-mers
8	Gene content	SNP	NT 8-mers	NT 8-mers
9	NT 8-mers	AA 3-mers	SNP	AA 3-mers
10	AA 3-mers	Gene content	AA 3-mers	SNP

**Figure A8:**
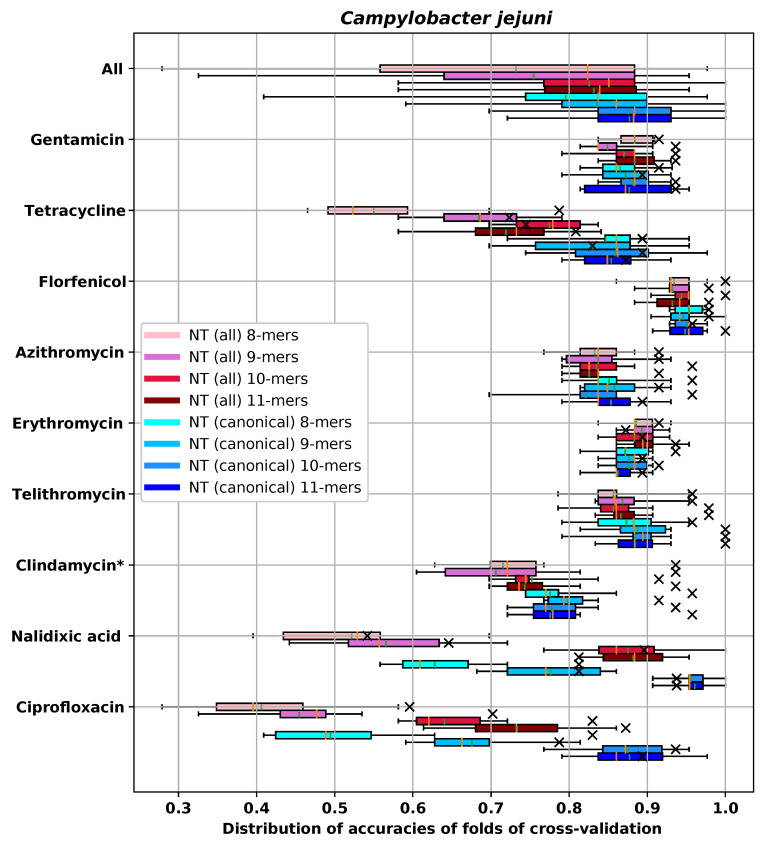
Distribution of accuracies in different nucleotide *k*-mers for *C. jejuni*. Plots are similar to Figure 5.

**Figure A9:**
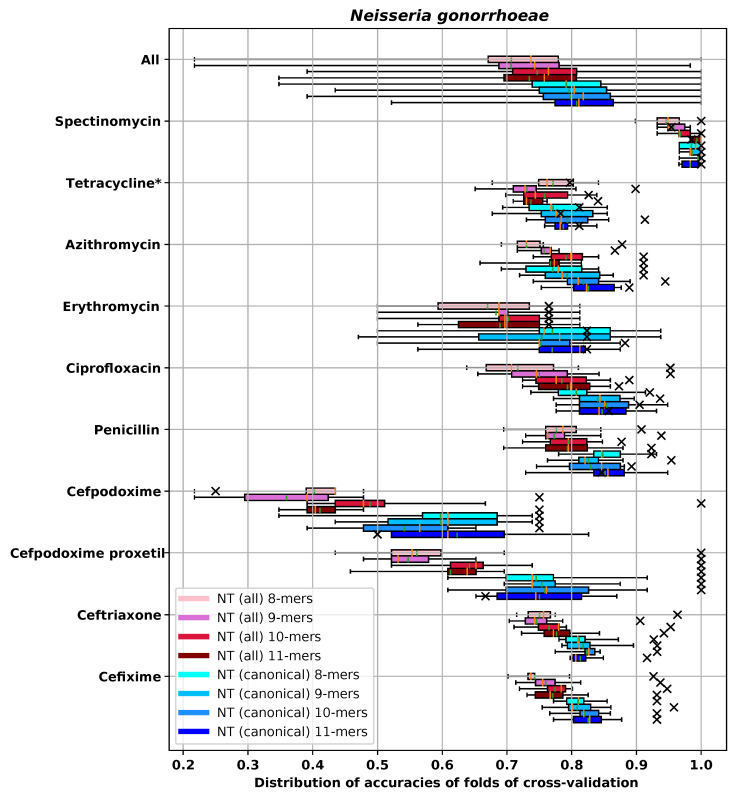
Distribution of accuracies in different nucleotide *k*-mers for *N. gonorrhoeae*. Plots are similar to Figure 5.

**Figure A10:**
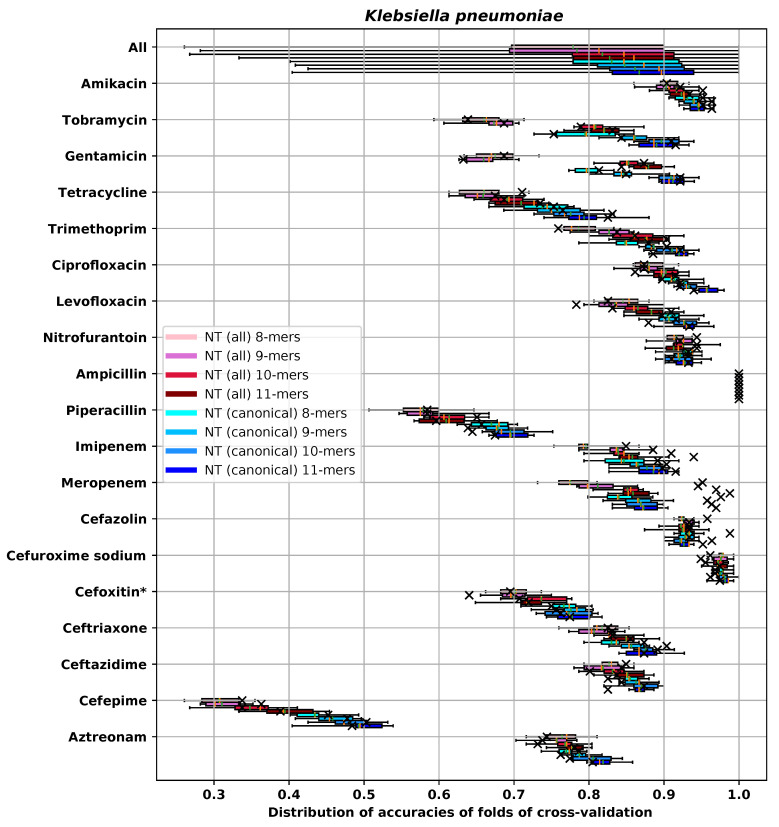
Distribution of accuracies in different nucleotide *k*-mers for *K. pneumoniae*. Plots are similar to Figure 5.

**Figure A11:**
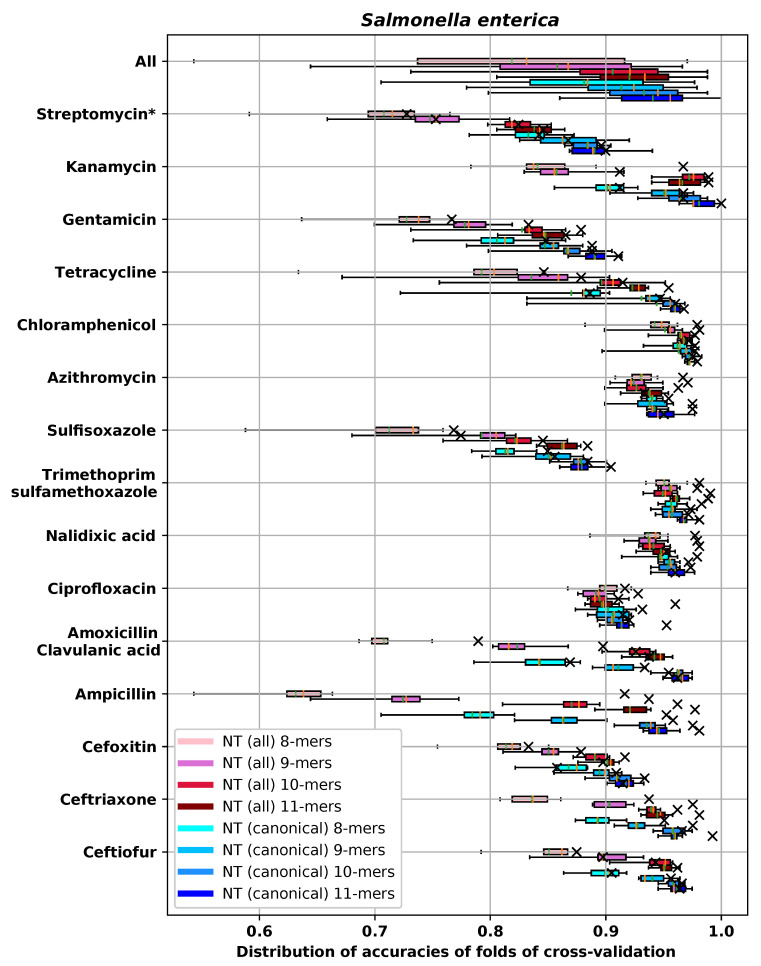
Distribution of accuracies in different nucleotide *k*-mers for *S. enterica*. Plots are similar to Figure 5.

**Figure A12:**
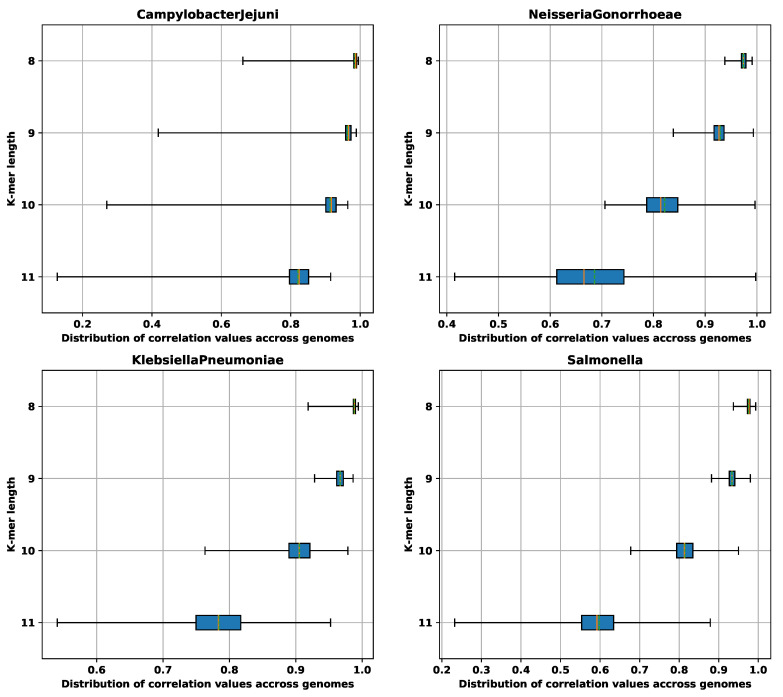
Distribution of correlation coefficient between the frequencies of canonical and non-canonical features, when both frequencies are counted separately across the genomes for different microbes and different *k*-mer lengths. In each box plot, the whiskers represents the maximum and minimum. The boxes represent the first and the third quartiles. The orange line represents the median and the green line represents the mean.

## Supplementary Materials

The following are available online at https://www.mdpi.com/2079-7737/9/11/365/s1.

## Appendix A. Number of Strains and MIC Distributions

**Table A1:** *C. jejuni*. Number of genomes for each antibiotic.

Antibiotic	Initial Number of Strains	Number of Strains after Filtering
erythromycin	481	476
azithromycin	481	477
gentamicin	481	478
clindamycin	481	479
telithromycin	481	472
ciprofloxacin	481	478
nalidixic acid	481	481
tetracycline	481	478
florfenicol	481	474

**Table A2:** *N. gonorrhoeae*. Number of genomes for each antibiotic.

Antibiotic	Initial Number of Strains	Number of Strains after Filtering
ceftriaxone	1926	1917
tetracycline	696	693
erythromycin	179	178
cefpodoxime	236	235
spectinomycin	655	653
cefixime	1907	1903
cefpodoximeproxetil	236	235
penicillin	655	650
azithromycin	911	905
ciprofloxacin	655	639

**Table A3:** *K. pneumoniae*. Number of genomes for each antibiotic.

Antibiotic	Initial Number of Strains	Number of Strains after Filtering
Aztreonam	1644	1644
Cefoxitin	1645	1645
Meropenem	1660	1652
Tobramycin	1666	1666
Gentamicin	1667	1667
Imipenem	1666	1665
Levofloxacin	1666	1666
Nitrofurantoin	895	895
Ampicillin	1666	1662
Tetracycline	1667	1667
Ceftazidime	1667	1667
Amikacin	1667	1667
Ceftriaxone	1667	1667
Cefuroximesodium	1575	1575
Cefazolin	1667	1667
Cefepime	1571	1571
Ciprofloxacin	1664	1664
Piperacillin	1662	1662
Trimethoprim	1667	1667

**Table A4:** *S. enterica*. Number of genomes for each antibiotic.

Antibiotic	Initial Number of Strains	Number of Strains after Filtering
Ampicillin	5277	5269
Amoxicillin-clavulanic acid	5278	5278
Ceftriaxone	5278	5276
Azithromycin	2416	2416
Chloramphenicol	5277	5277
Ciprofloxacin	5277	5276
Trimethoprim-sulfamethoxazole	5277	5271
Sulfisoxazole	4929	4929
Cefoxitin	5278	5278
Gentamicin	5278	5278
Kanamycin	924	919
Nalidixic acid	5278	5277
Streptomycin	2791	2791
Tetracycline	5277	5277
Ceftiofur	5278	5278

## Appendix B. Comparison of Number of Features for Nucleotide and Amino Acid k-mers

A nucleotide *k*-mer of length *k* is equivalent to an amino acid *k*-mer of length k/3. Since each nucleotide has 4 choices and each amino acid has 20 choices, if all nucleotide *k*-mers are counted, the ratio of maximum number of nucleotide *k*-mers to maximum number of amino acid *k*-mers will be

(A1) $$\frac{4^{k}}{20^{k/3}}=\frac{4^{k}}{{\left(20^{1/3}\right)}^{k}}={\left(\frac{4}{20^{1/3}}\right)}^{k}\approx 1.4736^{k}$$

Now we calculate the same ratio for the scenario where only canonical nucleotide *k*-mers are counted. For odd *k*s the maximum number of possible features will be halved because of half of the *k*-mers are non-canonical. For even *k*-mers, the number is more than half, because there are palindromic *k*-mers. Palindromic *k*-mers are *k*-mers that are equal to their reverse compliments. For example, the reverse compliment of “AATT” is “AATT.” If all of possible *k*-mers are listed alphabetically, all of the *k*-mers in the first half of the list will be canonical. In the second half, only palindromic *k*-mers will be canonical because the canonical counter parts of the rest have been considered in the first part of the list. Hence, the total number of theoretically possible canonical *k*-mers for an even *k* is the number of *k*-mers in the first half of the list, which is $(k^{4})/2$, plus the number of the palindromic *k*-mers is the second half. Now, we must count the number of palindromic *k*-mers in the second half of the list. We argue that the first base must be G or T; otherwise the *k*-mer would not be in the second half. Moreover, if a palindromic *k*-mer is divided into 2 chunks of the same length, the second chunk must be the reverse compliment of the first chunk to make it palindromic, so the second chunk is a function of first chunk. To count all palindromic *k*-mer we just have to count all possible combinations of the first chunk, which has k/2 bases. The first base has 2 options (G or T) and the rest of the k/2−1 bases have 4 options each. Hence, the total number of palindromic *k*-mers in the second half of the list will be

(A2) $$2\times 4^{\left(\frac{k}{2}-1\right)}=\frac{2\times 4^{k/2}}{4}=\frac{4^{k/2}}{2}=\frac{{\left(2^{2}\right)}^{k/2}}{2}=\frac{2^{k}}{2}=2^{k-1}$$

Thus, the total number of canonical *k*-mers for an even *k* will be

(A3) $$\frac{1}{2}\times \left(4^{k}\right)+2^{k-1}=\frac{1}{2}\left(4^{k}+2^{k}\right)$$

Thus, generally the total number of canonical *k*-mers will be:

(A4) $$\mathrm{Number}\;\mathrm{of}\;\mathrm{canonical}\;\text{k-mers}=\left\{\begin{array}{cc} \frac{1}{2}4^{k} & \;\mathrm{if}\;k\;\mathrm{is}\;\mathrm{odd}\\ \frac{1}{2}\left(4^{k}+2^{k}\right) & \;\mathrm{if}\;k\;\mathrm{is}\;\mathrm{even} \end{array}\right.$$

Therefore, for odd *k*s the ratio of total number of amino acid *k*-mers to total number of canonical nucleotide *k*-mers will be $\frac{1}{2}\frac{4^{k}}{20^{k/3}}\approx \frac{1}{2}1.4736^{k}$ and for an even *k*, the ratio will be $\frac{1}{2}\frac{4^{k}+2^{k}}{20^{k/3}}\approx \frac{1}{2}\left(1.4736^{k}+0.7368^{k}\right)$ or in a more compact form:

(A5) $$\left\{\begin{array}{cc} \frac{1}{2}\frac{4^{k}}{20^{k/3}}\approx \frac{1}{2}1.4736^{k} & \;\mathrm{if}\;k\;\mathrm{is}\;\mathrm{odd}\\ \frac{1}{2}\frac{4^{k}+2^{k}}{20^{k/3}}\approx \frac{1}{2}\left(1.4736^{k}+0.7368^{k}\right)= & \;\mathrm{if}\;k\;\mathrm{is}\;\mathrm{even} \end{array}\right.$$

## Appendix C. Violin Plots of Error in Different Methods

To visualize the prediction error in different methods, we collected the predicted MIC values vs. the actual values of all predictions pooled across the 10 folds of cross-validation. For example, if the actual value of MIC in one strain is 2, and the model predicts it to be 3, this would create the data-point (2, 3). Then, we grouped all those pairs by the actual values and generated a violin plot of distribution of the predictions for each target value. In these plots, the top and bottom horizontal lines represent minimum and maximum predicted values and the middle horizontal line represents the mean predicted value. The body of the violin is the kernel density estimation of the data. The ±1 two-fold dilution accuracy of that target value, and in parentheses, the number of strains with that actual value (i.e., the target sample size) is labeled under each violin. The green line represents a 1-to-1 perfect prediction, and the yellow line represents the first order regression between the actual values and the predicted values. The red lines represent the limits of ±1 two-fold dilution for the predictions of the model.

Such plots are presented for predictions of tetracycline for *C. jejuni* in Figure A5. They show how the actual versus predicted MIC values vary from a perfect prediction, within or outside the bounds provided by the two-fold dilution error. Using SNP features, the correlation between the actual values and the predictions is not as strong as in other methods and the slope of yellow regression line is not close to the 45 degrees.

In Figure A6, such plots are provided for *C. jejuni* and nalidixic acid. Here SNP features perform better than gene content method.

## Appendix D. Important Features Found by the Models

**Table A5:** Important features found for *K. pneumoniae* and tetracycline by amino acid 5-mer and gene content methods.

Amino Acid 5-mers					Gene Content			
5-mer	Gene Product	Number of +/− Strains	Mean (STD) MIC of +/− Strains	*p*-Value	Gene Product	Number of +/− Strains	Mean (STD) MIC of +/− Strains	*p*-Value
DGLTT	tetracycline resistance transcriptional repressor TetR	635/1032	15.0 (3.3)/6.3 (5.1)	$5\times 10^{-160}$	Permease of the drug/metabolite transporter (DMT) superfamily	341/1326	15.8 (1.6)/8.0 (5.9)	$7.2\times 10^{-87}$
HYGIL	tetracycline efflux MFS transporter Tet(A)	642/1025	15.0 (3.3)/6.2 (5.0)	$2.5\times 10^{-162}$	Tetracycline resistance regulatory protein TetR	282/1385	14.5 (4.0)/8.6 (6.0)	$5.3\times 10^{-45}$
IQWLI	undecaprenyl- phosphate galactose phosphotransferase WbaP	113/1554	12.1 (5.7)/9.4 (6.2)	$3.6\times 10^{-5}$	hypothetical protein (fig573.12878.peg.5387)	115/1552	7.0 (6.8)/9.8 (6.1)	$2.4\times 10^{-11}$
LRHCC		41/1626	5.2 (4.3)/9.7 (6.2)	$10^{-5}$	Tetracycline resistance, MFS efflux pump => Tet(D)	73/1594	5.9 (4.6)/9.8 (6.2)	$2.5\times 10^{-6}$
					Tetracycline resistance regulatory protein TetR	367/1300	15.8 (1.6)/7.9 (5.8)	$1.2\times 10^{-95}$
					Tetracycline resistance, MFS efflux pump => Tet(A)	366/1301	15.8 (1.6)/7.9 (5.8)	$2.6\times 10^{-95}$
					Tetracycline resistance, MFS efflux pump => Tet(D)	250/1417	15.7 (1.8)/8.5 (6.0)	$3.9\times 10^{-59}$
					Class A beta-lactamase (EC 3.5.2.6) => CTX-M family, extended-spectrum	1003/664	11.3 (5.9)/7.0 (5.6)	$1.4\times 10^{-44}$
					hypothetical protein (fig573.12921.peg.5397)	14/1653	2.4 (3.8)/9.7 (6.1)	$2\times 10^{-8}$
					Putative aminotransferase	11/1656	5.9 (6.5)/9.6 (6.2)	0.0061
					FIG002577: Putative lipoprotein precursor	205/1462	13.8 (4.3)/9.0 (6.2)	$8.3\times 10^{-25}$
					hypothetical protein (fig573.12941.peg.329)	18/1649	4.2 (5.3)/9.7 (6.1)	$1.3\times 10^{-6}$

**Table A6:** Important genes found for *K. pneumoniae* and tobramycin by amino acid 5-mer gene content methods.

Amino Acid 5-mers					Gene Content			
5-mer	Gene Product	Number of +/− Strains	Mean (STD) MIC of +/− Strains	*p*-Value	Gene Product	Number of +/− Strains	Mean (STD) MIC of +/− Strains	*p*-Value
PYEET	aminoglycoside N-acetyltransferase AAC(3)-IIe	627/1039	9.8 (5.7)/9.0 (6.5)	0.0016	Mobile element protein	324/1342	15.6 (2.0)/7.8 (6.0)	$1.3\times 10^{-85}$
QFLRK	OXA-1 family class D beta-lactamase	764/902	14.2 (3.7)/5.2 (4.9)	$1.9\times 10^{-183}$	Class D beta-lactamase (EC 3.5.2.6) => OXA-1 family	379/1287	13.4 (3.9)/8.1 (6.3)	$1.1\times 10^{-46}$
GSEMC		160/1506	6.2 (6.5)/9.7 (6.1)	$5.9\times 10^{-22}$	Chloramphenicol O-acetyltransferase (EC 2.3.1.28) => CatB family	354/1312	13.3 (3.9)/8.3 (6.4)	$5\times 10^{-41}$
DASMV	aminoglycoside N-acetyltransferase AAC(3)-IIe	1476/190	9.7 (6.3)/6.5 (4.9)	$2.4\times 10^{-10}$	Aminoglycoside N(6’)-acetyltransferase (EC 2.3.1.82) => AAC(6’)-Ib/AAC(6’)-II	681/985	14.0 (3.7)/6.1 (5.6)	$6\times 10^{-141}$
					Mobile element protein	58/1608	12.0 (5.9)/9.2 (6.3)	0.0015
					hypothetical protein (fig573.14286.peg.4244)	379/1287	7.3 (5.2)/9.9 (6.4)	$1.1\times 10^{-9}$
					hypothetical protein (fig573.14233.peg.3013)	431/1235	15.2 (3.0)/7.3 (5.8)	$7.5\times 10^{-108}$
					Aminoglycoside 3”-nucleotidyltransferase (EC 2.7.7.-) => ANT(3”)-Ia (AadA family)	84/1582	13.9 (4.7)/9.1 (6.2)	$2.3\times 10^{-11}$
					hypothetical protein (fig573.12921.peg.5397)	14/1652	2.1 (3.9)/9.4 (6.2)	$8.5\times 10^{-9}$
					hypothetical protein (fig573.14286.peg.510)	619/1047	5.9 (5.1)/11.3 (6.0)	$1.7\times 10^{-57}$
					hypothetical protein (fig573.13822.peg.6818)	17/1649	5.8 (5.3)/9.4 (6.3)	0.025
					Mobile element protein	38/1628	5.9 (4.5)/9.4 (6.3)	0.00096
					hypothetical protein (fig573.12887.peg.5713)	220/1446	14.2 (3.7)/8.6 (6.2)	$2.2\times 10^{-34}$

**Table A7:** Important genes found for *K. pneumoniae* and imipenem by amino acid 5-mer gene content methods.

Amino Acid 5-mers					Gene Content			
5-mer	Gene Product	Number of +/− Strains	Mean (STD) MIC of +/− Strains	*p*-Value	Gene Product	Number of +/− Strains	Mean (STD) MIC of +/− Strains	*p*-Value
SEPTR		169/1497	1.8 (4.4)/4.8 (6.1)	1.2e-76	Heat shock protein 60 kDa family chaperone GroEL	11/1655	16.0 (0.0)/4.4 (6.0)	$1.4\times 10^{-7}$
TCGVY	carbapenem- hydrolyzing class A beta-lactamase KPC	487/1179	12.2 (5.3)/1.3 (2.1)	$3.1\times 10^{-240}$	hypothetical protein (fig573.12981.peg.5967)	31/1635	3.1 (5.8)/4.5 (6.0)	$3.2\times 10^{-11}$
CIRDR		162/1504	1.6 (4.0)/4.8 (6.1)	$1.6\times 10^{-76}$	Anaerobic dimethyl sulfoxide reductase chain B (EC 1.8.5.3), iron-sulfur binding subunit	261/1405	2.4 (4.7)/4.8 (6.2)	$1.5\times 10^{-54}$
GWIKI	16S rRNA (guanine(1405)- N(7))- methyltransferase	15/1651	13.1 (5.1)/4.4 (6.0)	$1.5\times 10^{-6}$	hypothetical protein (fig573.14374.peg.5559)	11/1655	14.9 (3.4)/4.4 (6.0)	$4.4\times 10^{-7}$
LDFPD	BNR-4 repeat-containing protein	16/1650	12.1 (6.1)/4.4 (6.0)	0.00011	Periplasmic divalent cation tolerance protein CutA	11/1655	16.0 (0.0)/4.4 (6.0)	$1.4\times 10^{-7}$
WELEL	KPC family carbapenem- hydrolyzing class A beta-lactamase	491/1175	12.2 (5.4)/1.3 (2.1)	$2.4\times 10^{-239}$	hypothetical protein (fig573.12878.peg.5387)	115/1551	1.6 (4.1)/4.7 (6.1)	$1.8\times 10^{-56}$
					Transposase	477/1189	12.3 (5.3)/1.3 (2.3)	$2.6\times 10^{-232}$
					Class A beta-lactamase (EC 3.5.2.6) => KPC family, carbapenem-hydrolyzing	488/1178	12.2 (5.4)/1.3 (2.2)	$1.6\times 10^{-236}$
					Transposase InsH for insertion sequence element IS5	10/1656	0.2 (0.0)/4.5 (6.0)	$4.9\times 10^{-9}$
					Mobile element protein	9/1657	1.4 (2.3)/4.5 (6.0)	0.0074
					hypothetical protein (fig573.13500.peg.3164)	186/1480	1.3 (2.2)/4.9 (6.2)	$3.6\times 10^{-11}$
					hypothetical protein (fig573.14417.peg.5283)	6/1660	11.0 (7.1)/4.4 (6.0)	0.025
					Mobile element protein	479/1187	12.3 (5.3)/1.3 (2.2)	$2.3\times 10^{-233}$
					Mobile element protein	56/1610	1.4 (2.1)/4.6 (6.1)	0.036
					IS, phage, Tn; Transposon-related functions	10/1656	14.8 (3.6)/4.4 (6.0)	$1.6\times 10^{-6}$

**Table A8:** Important genes found for *S. enterica* and cefoxitin by amino acid 5-mer gene content method.

Amino Acid 5-mers					Gene Content			
5-mer	Gene Product	Number of +/− Strains	Mean (STD) MIC of +/− Strains	*p*-Value	Gene Product	Number of +/− Strains	Mean (STD) MIC of +/− Strains	*p*-Value
ANKSY	CMY-2 family class C beta-lactamase	714/4458	46.0 (18.7)/3.1 (3.3)	0.0	Outer membrane porin OmpD	4172/1106	8.8 (16.5)/10.3 (17.4)	$1.7\times 10^{-189}$
TWITV	CMY-2 family class C beta-lactamase	713/4459	45.9 (18.7)/3.1 (3.4)	0.0	Mobile element protein	840/4438	38.2 (23.2)/3.6 (6.2)	0.0
GNTHP		9/5163	11.6 (18.7)/9.0 (16.6)	0.048	Class C beta-lactamase (EC 3.5.2.6) => CMY/CMY-2/CFE/LAT family	736/4542	45.7 (18.7)/3.2 (3.7)	0.0
VRTFP		110/5062	4.4 (7.8)/9.1 (16.7)	0.00044	Putative outer membrane lipoprotein	6/5272	16.0 (0.0)/9.1 (16.7)	0.00088
QNTRI		12/5160	20.3 (23.0)/9.0 (16.6)	0.049	Small multidrug resistance (SMR) efflux transporter => SugE, quaternary ammonium compounds	696/4582	46.0 (18.6)/3.5 (5.4)	0.0
HTWIT	CMY-2 family class C beta-lactamase	713/4459	45.9 (18.7)/3.1 (3.4)	0.0	FIG01046993: hypothetical protein	440/4838	42.5 (23.5)/6.1 (12.0)	$2.6\times 10^{-176}$
					Cobalamin synthase (EC 2.7.8.26)	5/5273	33.6 (26.8)/9.1 (16.7)	0.0048

**Table A9:** Important genes found for *S. enterica* and ampicillin by amino acid 5-mer and gene content methods.

Amino Acid 5-mers					Gene Content			
5-mer	Gene Product	Number of +/− Strains	Mean (STD) MIC of +/− Strains	*p*-Value	Gene Product	Number of +/− Strains	Mean (STD) MIC of +/− Strains	*p*-Value
WMRDD	class A beta-lactamase	56/5221	62.9 (8.3)/19.7 (28.6)	$4.7\times 10^{-24}$	Class A beta-lactamase (EC 3.5.2.6) => TEM family	815/4462	63.2 (7.1)/12.3 (23.9)	0.0
TDFLR	class A beta-lactamase	143/5134	45.5 (28.7)/19.4 (28.5)	$3\times 10^{-20}$	Class A beta-lactamase (EC 3.5.2.6) => HER family	56/5221	62.9 (8.3)/19.7 (28.6)	$4.7\times 10^{-24}$
ASWVH	CMY-2 family class C beta-lactamase	745/4532	62.9 (8.1)/13.1 (24.6)	0.0	Mobile element protein	294/4983	63.8 (3.7)/17.6 (27.6)	$2.7\times 10^{-130}$
AMSQN	response regulator transcription factor	10/5267	45.4 (28.4)/20.1 (28.8)	0.0051	hypothetical protein (fig590.14012.peg.5176)	106/5171	63.4 (6.0)/19.2 (28.4)	$1.8\times 10^{-46}$
TMSDN	TEM family class A beta-lactamase	1091/4186	57.2 (19.5)/10.5 (22.3)	0.0	Class A beta-lactamase (EC 3.5.2.6) => CARB/PSE family, carbenicillin-hydrolyzing	42/5235	61.1 (13.1)/19.8 (28.7)	$10^{-17}$
RDIGY		26/5251	49.6 (26.3)/20.0 (28.8)	$1.2\times 10^{-7}$	Integrase	5/5272	64.0 (0.0)/20.1 (28.8)	0.002
NTAAN	type IV conjugative transfer system coupling protein TraD	868/4409	62.9 (8.3)/11.7 (23.5)	0.0	DNA replication protein	26/5251	49.6 (26.3)/20.0 (28.8)	$1.2\times 10^{-7}$
VIYQG	CMY-2 family class C beta-lactamase	751/4526	62.5 (9.5)/13.1 (24.6)	0.0	Class C beta-lactamase (EC 3.5.2.6) => CMY/CMY-2/CFE/LAT family	736/4541	63.1 (7.5)/13.2 (24.7)	0.0
YWDYN	TolC family protein	42/5235	61.1 (13.1)/19.8 (28.7)	$1\times 10^{-17}$	ABC transporter involved in cytochrome c biogenesis, ATPase component CcmA	4/5273	64.0 (0.0)/20.1 (28.8)	0.0056
					hypothetical protein (fig590.17530.peg.3704)	4/5273	64.0 (0.0)/20.1 (28.8)	0.0056

**Table A10:** Important genes found for *S. enterica* and chloramphenicol by amino acid 5-mer and gene content methods.

Amino Acid 5-mers					Gene Content			
5-mer	Gene Product	Number of +/− Strains	Mean (STD) MIC of +/− Strains	*p*-Value	Gene Product	Number of +/− Strains	Mean (STD) MIC of +/− Strains	*p*-Value
WAYTL	chloramphenicol/florfenicol efflux MFS transporter FloR	146/5131	63.3 (6.1)/6.8 (3.7)	$8.8\times 10^{-125}$	Chloramphenicol resistance, MFS efflux pump => CmlA family	26/5251	56.0 (14.9)/8.2 (9.4)	$5.1\times 10^{-23}$
TAWPV	CmlA family chloramphenicol efflux MFS transporter	169/5108	57.7 (17.3)/6.8 (3.3)	$1.1\times 10^{-117}$	Chloramphenicol/florfenicol resistance, MFS efflux pump => FloR family	146/5131	63.3 (6.1)/6.8 (3.7)	$8.8\times 10^{-125}$
CDGFH	type A chloramphenicol O-acetyltransferase	6/5271	64.0 (0.0)/8.3 (9.8)	$1.8\times 10^{-6}$	hypothetical protein (fig590.14843.peg.2228)	4061/1216	9.4 (10.9)/5.2 (4.9)	$4\times 10^{-266}$
QGSGN	Select seq gbEAX8474651.1 RHS repeat protein	1308/3969	6.4 (9.9)/9.0 (10.0)	$5.1\times 10^{-259}$	Small multidrug resistance (SMR) efflux transporter => QacE, quaternary ammonium compounds	13/5264	47.4 (18.9)/8.3 (9.8)	$4.8\times 10^{-11}$
YMVML	chloramphenicol/florfenicol efflux MFS transporter FloR	146/5131	63.3 (6.1)/6.8 (3.7)	$8.8\times 10^{-125}$	Transposase, IS3/IS911 family	692/4585	6.5 (5.8)/8.7 (10.5)	$5.9\times 10^{-20}$
MDIYL	chloramphenicol/florfenicol efflux MFS transporter FloR	146/5131	63.3 (6.1)/6.8 (3.7)	$8.8\times 10^{-125}$	Glycosyltransferase	1179/4098	6.6 (10.3)/8.9 (9.9)	$6.8\times 10^{-221}$
FRMAM	amino acid adenylation domain-containing protein	6/5271	64.0 (0.0)/8.3 (9.8)	$1.8\times 10^{-6}$	Transcriptional regulator, LysR family	107/5170	64.0 (0.0)/7.2 (6.1)	$3.4\times 10^{-92}$
PVFTM	type A chloramphenicol O-acetyltransferase	9/5268	45.8 (25.9)/8.3 (9.8)	$1.7\times 10^{-5}$	Tetracycline resistance regulatory protein TetR	38/5239	61.3 (11.6)/8.0 (8.9)	$3\times 10^{-32}$
FAKFF	type IV secretion system protein TraC	7/5270	17.1 (19.3)/8.4 (10.0)	0.0094	Inner membrane protein of type IV secretion of T-DNA complex, TonB-like, VirB10	6/5271	17.3 (20.9)/8.4 (10.0)	0.041
					Cytochrome c-type heme lyase subunit nrfE, nitrite reductase complex assembly	1960/3317	10.3 (13.4)/7.3 (7.1)	$2.5\times 10^{-31}$
					Type I secretion system ATPase, LssB family LapB	41/5236	5.3 (2.0)/8.4 (10.0)	$2.8\times 10^{-5}$
					hypothetical protein (fig590.13820.peg.5180)	48/5229	52.2 (22.0)/8.0 (8.9)	$5.4\times 10^{-34}$
					YbjA protein	4/5273	64.0 (0.0)/8.4 (9.9)	$9.7\times 10^{-5}$
					Chloramphenicol O-acetyltransferase (EC 2.3.1.28) => CatA1/CatA4 family	4/5273	64.0 (0.0)/8.4 (9.9)	$9.7\times 10^{-5}$
					Transcriptional regulator, LysR family	2478/2799	8.3 (8.7)/8.5 (11.1)	$2.6\times 10^{-17}$
					Mobile element protein	119/5158	59.9 (14.5)/7.2 (5.9)	$8.9\times 10^{-91}$
					Phosphomannomutase (EC 5.4.2.8)	1180/4097	6.6 (10.3)/8.9 (9.9)	$1.8\times 10^{-220}$
					Mercuric transport protein, MerT	450/4827	18.8 (22.3)/7.4 (7.2)	$2.3\times 10^{-59}$

**Table A11:** Important genes found for *S. enterica* and sulfisoxazole by amino acid 5-mer and gene content methods.

Amino Acid 5-mers					Gene Content			
5-mer	Gene Product	Number of +/− Strains	Mean (STD) MIC of +/− Strains	*p*-Value	Gene Product	Number of +/− Strains	Mean (STD) MIC of +/− Strains	*p*-Value
LDPGM	sulfonamide- resistant dihydropteroate synthase Sul1	1565/3364	501.2 (69.6)/52.1 (59.6)	0.0	Mobile element protein	22/4907	512.0 (0.0)/193.3 (217.8)	$8\times 10^{-9}$
GMGFF	sulfonamide- resistant dihydropteroate synthase Sul1	1552/3377	505.0 (56.1)/52.1 (59.4)	0.0	Dihydropteroate synthase type-2 (EC 2.5.1.15) @ Sulfonamide resistance protein	578/4351	506.7 (49.0)/153.3 (197.6)	$3.6\times 10^{-209}$
					hypothetical protein (fig590.17526.peg.3772)	802/4127	506.2 (51.5)/134.2 (184.1)	$5.4\times 10^{-305}$
					Aminoglycoside 3”-nucleotidyltransferase (EC 2.7.7.-) => ANT(3”)-Ia (AadA family)	138/4791	508.8 (38.0)/185.7 (214.6)	$8.6\times 10^{-48}$
					Dihydropteroate synthase type-2 (EC 2.5.1.15) @ Sulfonamide resistance protein	985/3944	506.4 (50.7)/116.9 (169.1)	0.0
					DNA recombination protein RmuC	18/4911	512.0 (0.0)/193.6 (217.9)	$1.8\times 10^{-7}$
					Muconolactone isomerase (EC 5.3.3.4),putative	818/4111	485.0 (108.4)/137.0 (186.3)	$1.6\times 10^{-274}$

## Appendix E. Performance Evaluation

In this section, performances of the different methods are evaluated using different metrics described in Section 2.4. The metrics are root mean square (RMSE), ±1 two-fold accuracy (DD1), ±2 two-fold accuracy (DD2), major error rate (ME) and very major error rate (VME). For each metric, we report the mean and standard deviation of cross-validation labeled as CV and the value for the held out set, labeled as H. For example, RMSE-CV means RMSE in cross-validation, which is reported in terms of mean and standard deviation, in parentheses, across 10 folds. For the hold-out set, only one value is reported. Results of each species—antibiotic combinations are presented in a separate table. In each column, if one of the methods performed better than others we plotted it in bold face. Since currently, there are no approved breakpoints for erythromycin and *N. gonorrhoeae*, major error rate and and very major error rate were not calculated for this data set.

### Appendix E.1. C. Jejuni

**Table A12:** *C. jejuni*–erythromycin.

	RMSE-CV	RMSE-H	DD1-CV	DD1-H	DD2-CV	DD2-H	ME-CV	ME-H	VME-CV	VME-H
NT 8-mers	0.65 (0.027)	**0.457**	0.872 (0.03)	**0.936**	0.991 (0.011)	1.000	0.0 (0.0)	0.0	0.0 (0.0)	0.0
NT 9-mers	0.626 (0.038)	0.587	0.877 (0.021)	0.894	0.995 (0.009)	0.979	0.0 (0.0)	0.0	0.0 (0.0)	0.0
NT 10-mers	0.607 (0.038)	0.554	0.877 (0.025)	0.915	1.0 (0.0)	1.000	0.0 (0.0)	0.0	0.0 (0.0)	0.0
NT 11-mers	0.622 (0.035)	0.586	0.862 (0.025)	0.894	0.998 (0.007)	1.000	0.0 (0.0)	0.0	0.0 (0.0)	0.0
AA 3-mers	0.615 (0.03)	0.553	0.888 (0.023)	0.915	0.995 (0.009)	1.000	0.0 (0.0)	0.0	0.0 (0.0)	0.0
AA 4-mers	0.604 (0.034)	0.581	0.877 (0.029)	0.915	0.998 (0.007)	1.000	0.0 (0.0)	0.0	0.0 (0.0)	0.0
AA 5-mers	**0.598 (0.042)**	0.566	0.895 (0.03)	0.915	1.0 (0.0)	1.000	0.0 (0.0)	0.0	0.0 (0.0)	0.0
Gene content	0.6 (0.041)	0.605	**0.902 (0.014)**	0.872	1.0 (0.0)	1.000	0.0 (0.0)	0.0	0.0 (0.0)	0.0
SNP	0.624 (0.046)	0.593	0.858 (0.026)	0.915	0.998 (0.007)	0.979	0.0 (0.0)	0.0	0.0 (0.0)	0.0
Gene content + SNP	0.612 (0.048)	0.596	0.898 (0.033)	0.894	1.0 (0.0)	1.000	0.0 (0.0)	0.0	0.0 (0.0)	0.0

**Table A13:** *C. jejuni*–azithromycin.

	RMSE-CV	RMSE-H	DD1-CV	DD1-H	DD2-CV	DD2-H	ME-CV	ME-H	VME-CV	VME-H
NT 8-mers	0.698 (0.052)	0.503	0.851 (0.039)	0.957	0.993 (0.011)	0.979	0.0 (0.0)	0.0	0.0 (0.0)	0.0
NT 9-mers	0.696 (0.061)	0.549	0.858 (0.04)	0.915	0.993 (0.015)	0.979	0.0 (0.0)	0.0	0.0 (0.0)	0.0
NT 10-mers	0.729 (0.06)	0.49	0.821 (0.05)	0.957	0.993 (0.011)	1.000	0.0 (0.0)	0.0	0.0 (0.0)	0.0
NT 11-mers	0.717 (0.042)	0.538	0.853 (0.036)	0.894	0.998 (0.007)	1.000	0.0 (0.0)	0.0	0.0 (0.0)	0.0
AA 3-mers	0.699 (0.073)	**0.484**	0.853 (0.051)	0.936	0.995 (0.009)	1.000	0.0 (0.0)	0.0	0.0 (0.0)	0.0
AA 4-mers	0.689 (0.058)	0.572	0.856 (0.052)	0.915	0.998 (0.007)	0.979	0.0 (0.0)	0.0	0.0 (0.0)	0.0
AA 5-mers	**0.683 (0.054)**	0.581	0.86 (0.053)	0.936	0.998 (0.007)	1.000	0.0 (0.0)	0.0	0.0 (0.0)	0.0
Gene content	0.687 (0.054)	0.693	**0.87 (0.032)**	0.787	1.0 (0.0)	1.000	0.0 (0.0)	0.0	0.0 (0.0)	0.0
SNP	0.729 (0.059)	0.582	0.835 (0.069)	0.936	0.998 (0.007)	1.000	0.0 (0.0)	0.0	0.0 (0.0)	0.0
Gene content + SNP	0.7 (0.063)	0.707	0.86 (0.036)	0.830	1.0 (0.0)	0.979	0.0 (0.0)	0.0	0.0 (0.0)	0.0

**Table A14:** *C. jejuni*–gentamicin.

	RMSE-CV	RMSE-H	DD1-CV	DD1-H	DD2-CV	DD2-H	ME-CV	ME-H	VME-CV	VME-H
NT 8-mers	0.657 (0.062)	0.507	0.865 (0.036)	0.915	0.993 (0.011)	1.0	0.0 (0.0)	0.0	0.0 (0.0)	0.0
NT 9-mers	0.664 (0.057)	0.502	0.872 (0.039)	0.894	0.988 (0.021)	1.0	0.0 (0.0)	0.0	0.0 (0.0)	0.0
NT 10-mers	0.665 (0.062)	0.505	0.882 (0.029)	0.936	0.991 (0.011)	1.0	0.0 (0.0)	0.0	0.0 (0.0)	0.0
NT 11-mers	0.653 (0.073)	**0.499**	0.877 (0.053)	0.936	0.993 (0.011)	1.0	0.0 (0.0)	0.0	0.0 (0.0)	0.0
AA 3-mers	0.656 (0.055)	0.504	0.863 (0.051)	0.894	0.991 (0.015)	1.0	0.0 (0.0)	0.0	0.0 (0.0)	0.0
AA 4-mers	0.644 (0.058)	0.547	0.886 (0.024)	0.894	0.993 (0.011)	1.0	0.0 (0.0)	0.0	0.0 (0.0)	0.0
AA 5-mers	0.654 (0.065)	0.528	0.877 (0.03)	0.915	0.993 (0.011)	1.0	0.0 (0.0)	0.0	0.0 (0.0)	0.0
Gene content	**0.622 (0.05)**	0.665	0.886 (0.019)	0.851	**0.998 (0.007)**	1.0	0.0 (0.0)	0.0	0.0 (0.0)	0.0
SNP	0.673 (0.071)	0.5	0.856 (0.024)	0.872	0.986 (0.024)	1.0	0.0 (0.0)	0.0	0.0 (0.0)	0.0
Gene content + SNP	0.64 (0.053)	0.547	0.868 (0.028)	0.936	0.993 (0.011)	1.0	0.0 (0.0)	0.0	0.0 (0.0)	0.0

**Table A15:** *C. jejuni*–clindamycin.

	RMSE-CV	RMSE-H	DD1-CV	DD1-H	DD2-CV	DD2-H	ME-CV	ME-H	VME-CV	VME-H
NT 8-mers	0.913 (0.077)	**0.49**	0.775 (0.036)	0.957	0.954 (0.031)	**1.0**	0.0 (0.0)	0.0	0.009 (0.011)	0.021
NT 9-mers	0.855 (0.075)	0.614	**0.799 (0.026)**	0.915	0.968 (0.028)	0.979	0.0 (0.0)	0.0	0.007 (0.011)	0.043
NT 10-mers	0.87 (0.1)	0.624	0.778 (0.034)	0.936	0.972 (0.017)	0.979	0.0 (0.0)	0.0	0.007 (0.011)	0.043
NT 11-mers	0.867 (0.077)	0.585	0.776 (0.033)	0.957	0.968 (0.033)	0.979	0.0 (0.0)	0.0	0.009 (0.011)	0.043
AA 3-mers	0.892 (0.057)	0.685	0.778 (0.023)	0.872	0.968 (0.021)	0.979	0.0 (0.0)	0.0	0.007 (0.01)	0.043
AA 4-mers	0.866 (0.086)	0.674	0.764 (0.032)	0.894	0.968 (0.029)	0.979	0.0 (0.0)	0.0	0.007 (0.01)	0.043
AA 5-mers	0.848 (0.066)	0.628	0.766 (0.043)	0.915	0.977 (0.023)	0.979	0.0 (0.0)	0.0	0.007 (0.01)	0.043
Gene content	**0.813 (0.073)**	0.835	0.798 (0.062)	0.809	**0.982 (0.02)**	0.979	0.0 (0.0)	0.0	0.007 (0.01)	0.043
SNP	0.908 (0.085)	0.59	0.746 (0.035)	0.936	0.956 (0.03)	0.979	0.0 (0.0)	0.0	0.009 (0.011)	0.021
Gene content + SNP	0.859 (0.066)	0.837	0.785 (0.046)	0.787	0.977 (0.023)	0.957	0.0 (0.0)	0.0	0.009 (0.011)	0.043

**Table A16:** *C. jejuni*–telithromycin.

	RMSE-CV	RMSE-H	DD1-CV	DD1-H	DD2-CV	DD2-H	ME-CV	ME-H	VME-CV	VME-H
NT 8-mers	0.631 (0.047)	0.394	0.873 (0.046)	0.957	0.991 (0.012)	1.0	0.0 (0.0)	0.0	0.0 (0.0)	0.0
NT 9-mers	0.639 (0.038)	0.344	0.887 (0.036)	1.000	0.995 (0.009)	1.0	0.0 (0.0)	0.0	0.0 (0.0)	0.0
NT 10-mers	0.629 (0.043)	0.389	0.885 (0.036)	1.000	0.993 (0.015)	1.0	0.0 (0.0)	0.0	0.0 (0.0)	0.0
NT 11-mers	0.624 (0.042)	0.366	0.885 (0.033)	1.000	0.993 (0.011)	1.0	0.0 (0.0)	0.0	0.0 (0.0)	0.0
AA 3-mers	0.624 (0.025)	0.401	0.885 (0.027)	0.957	0.995 (0.009)	1.0	0.0 (0.0)	0.0	0.0 (0.0)	0.0
AA 4-mers	0.625 (0.037)	0.385	0.885 (0.033)	1.000	0.993 (0.011)	1.0	0.0 (0.0)	0.0	0.0 (0.0)	0.0
AA 5-mers	0.606 (0.03)	0.465	**0.908 (0.025)**	0.957	0.995 (0.009)	1.0	0.0 (0.0)	0.0	0.0 (0.0)	0.0
Gene content	0.615 (0.043)	0.595	0.894 (0.026)	0.915	0.995 (0.01)	1.0	0.0 (0.0)	0.0	0.0 (0.0)	0.0
SNP	0.641 (0.055)	**0.308**	0.88 (0.043)	1.000	0.993 (0.015)	1.0	0.0 (0.0)	0.0	0.0 (0.0)	0.0
Gene content + SNP	**0.604 (0.034)**	0.535	0.904 (0.03)	0.957	0.995 (0.009)	1.0	0.0 (0.0)	0.0	0.0 (0.0)	0.0

**Table A17:** *C. jejuni*–ciprofloxacin.

	RMSE-CV	RMSE-H	DD1-CV	DD1-H	DD2-CV	DD2-H	ME-CV	ME-H	VME-CV	VME-H
NT 8-mers	2.35 (0.259)	1.708	0.494 (0.072)	0.830	0.736 (0.052)	0.915	0.035 (0.019)	0.000	0.095 (0.03)	0.064
NT 9-mers	1.629 (0.202)	0.915	0.675 (0.069)	0.787	0.863 (0.038)	0.936	0.016 (0.021)	0.000	0.032 (0.023)	0.000
NT 10-mers	0.936 (0.291)	0.618	0.875 (0.057)	0.936	0.979 (0.016)	1.000	0.005 (0.009)	0.000	0.007 (0.011)	0.000
NT 11-mers	0.929 (0.312)	0.587	0.877 (0.057)	0.894	0.977 (0.018)	1.000	0.005 (0.009)	0.000	0.009 (0.011)	0.000
AA 3-mers	2.357 (0.295)	1.7	0.499 (0.052)	0.787	0.691 (0.058)	0.872	0.03 (0.023)	0.000	0.091 (0.029)	0.064
AA 4-mers	1.053 (0.282)	0.698	0.828 (0.041)	0.894	0.951 (0.026)	0.979	0.002 (0.007)	0.000	0.014 (0.015)	0.000
AA 5-mers	**0.883 (0.32)**	**0.563**	**0.879 (0.043)**	0.936	**0.981 (0.02)**	1.000	0.002 (0.007)	0.000	0.007 (0.011)	0.000
Gene content	2.414 (0.181)	2.095	0.508 (0.056)	0.681	0.724 (0.044)	0.851	0.014 (0.029)	0.021	0.109 (0.036)	0.064
SNP	1.134 (0.355)	0.611	0.803 (0.056)	0.915	0.947 (0.031)	0.979	0.002 (0.007)	0.000	0.014 (0.018)	0.000
Gene content + SNP	1.208 (0.298)	0.797	0.759 (0.05)	0.851	0.928 (0.024)	0.957	0.002 (0.007)	0.000	0.012 (0.016)	0.000

**Table A18:** *C. jejuni*–nalidixic acid.

	RMSE-CV	RMSE-H	DD1-CV	DD1-H	DD2-CV	DD2-H	ME-CV	ME-H	VME-CV	VME-H
NT 8-mers	1.693 (0.136)	1.485	0.628 (0.05)	0.812	0.804 (0.043)	0.896	0.044 (0.024)	0.0	0.081 (0.016)	0.083
NT 9-mers	1.077 (0.165)	1.124	0.776 (0.066)	0.812	0.928 (0.036)	0.938	0.009 (0.015)	0.0	0.025 (0.013)	0.042
NT 10-mers	0.66 (0.223)	**0.563**	0.956 (0.024)	0.938	0.984 (0.018)	**1.0**	0.005 (0.009)	0.0	0.007 (0.01)	0.000
NT 11-mers	**0.643 (0.228)**	0.659	0.961 (0.026)	0.938	0.986 (0.015)	0.979	0.005 (0.009)	0.0	0.007 (0.01)	0.000
AA 3-mers	1.765 (0.187)	1.432	0.619 (0.067)	0.792	0.772 (0.066)	0.917	0.032 (0.021)	0.0	0.099 (0.023)	0.083
AA 4-mers	0.783 (0.168)	0.841	0.915 (0.041)	0.917	0.968 (0.021)	0.958	0.005 (0.009)	0.0	0.009 (0.011)	0.042
AA 5-mers	0.647 (0.215)	0.639	**0.963 (0.021)**	0.938	0.986 (0.015)	0.979	0.005 (0.009)	0.0	0.007 (0.01)	0.021
Gene content	1.788 (0.111)	1.573	0.536 (0.1)	0.729	0.779 (0.067)	0.896	0.025 (0.016)	0.0	0.09 (0.026)	0.083
SNP	0.847 (0.248)	0.779	0.861 (0.051)	0.896	0.975 (0.026)	0.958	0.007 (0.011)	0.0	0.014 (0.023)	0.021
Gene content + SNP	0.912 (0.241)	0.762	0.841 (0.063)	0.812	0.968 (0.034)	0.958	0.005 (0.009)	0.0	0.014 (0.023)	0.021

**Table A19:** *C. jejuni*–tetracycline.

	RMSE-CV	RMSE-H	DD1-CV	DD1-H	DD2-CV	DD2-H	ME-CV	ME-H	VME-CV	VME-H
NT 8-mers	1.145 (0.584)	2.533	0.847 (0.07)	0.894	0.956 (0.041)	0.915	0.012 (0.016)	0.043	0.007 (0.015)	0.021
NT 9-mers	1.016 (0.482)	2.525	0.831 (0.078)	0.83	0.977 (0.018)	0.894	0.005 (0.009)	0.043	0.005 (0.009)	0.021
NT 10-mers	1.007 (0.513)	**2.126**	**0.858 (0.069)**	0.894	0.97 (0.031)	0.936	0.007 (0.011)	**0.021**	0.005 (0.009)	0.021
NT 11-mers	0.97 (0.435)	2.424	0.854 (0.042)	0.872	0.979 (0.016)	0.936	0.005 (0.009)	0.043	0.005 (0.009)	0.021
AA 3-mers	1.183 (0.508)	2.417	0.803 (0.046)	0.872	0.956 (0.03)	0.915	0.009 (0.015)	0.043	0.007 (0.011)	0.021
AA 4-mers	**0.909 (0.359)**	2.463	0.849 (0.051)	**0.915**	**0.986 (0.015)**	0.936	0.005 (0.009)	0.043	**0.002 (0.007)**	0.021
AA 5-mers	0.923 (0.45)	2.533	0.856 (0.051)	0.83	0.977 (0.028)	0.936	0.005 (0.009)	0.043	0.005 (0.009)	0.021
Gene content	1.181 (0.425)	2.239	0.817 (0.041)	0.872	0.963 (0.021)	0.936	0.005 (0.009)	0.043	0.005 (0.009)	0.021
SNP	3.445 (0.544)	4.4	0.459 (0.07)	0.532	0.617 (0.065)	0.638	0.109 (0.039)	0.17	0.06 (0.028)	0.085
Gene content + SNP	1.111 (0.389)	2.279	0.801 (0.034)	0.894	0.963 (0.015)	0.936	0.007 (0.011)	0.043	0.005 (0.009)	0.021

**Table A20:** *C. jejuni*–florfenicol.

	RMSE-CV	RMSE-H	DD1-CV	DD1-H	DD2-CV	DD2-H	ME-CV	ME-H	VME-CV	VME-H
NT 8-mers	0.535 (0.024)	0.355	0.953 (0.018)	0.979	1.0 (0.0)	1.0	0.0 (0.0)	0.0	0.0 (0.0)	0.0
NT 9-mers	0.528 (0.043)	0.381	0.946 (0.026)	0.979	1.0 (0.0)	1.0	0.0 (0.0)	0.0	0.0 (0.0)	0.0
NT 10-mers	0.521 (0.048)	0.388	0.951 (0.017)	0.957	0.998 (0.007)	1.0	0.0 (0.0)	0.0	0.0 (0.0)	0.0
NT 11-mers	0.52 (0.036)	0.369	0.948 (0.023)	1.000	1.0 (0.0)	1.0	0.0 (0.0)	0.0	0.0 (0.0)	0.0
AA 3-mers	0.513 (0.036)	0.402	0.953 (0.021)	0.936	1.0 (0.0)	1.0	0.0 (0.0)	0.0	0.0 (0.0)	0.0
AA 4-mers	0.52 (0.039)	0.39	0.949 (0.017)	0.936	1.0 (0.0)	1.0	0.0 (0.0)	0.0	0.0 (0.0)	0.0
AA 5-mers	**0.51 (0.037)**	0.397	0.949 (0.023)	0.979	1.0 (0.0)	1.0	0.0 (0.0)	0.0	0.0 (0.0)	0.0
Gene content	0.511 (0.037)	0.501	0.953 (0.018)	0.936	1.0 (0.0)	1.0	0.0 (0.0)	0.0	0.0 (0.0)	0.0
SNP	0.537 (0.042)	**0.272**	0.934 (0.033)	1.000	1.0 (0.0)	1.0	0.0 (0.0)	0.0	0.0 (0.0)	0.0
Gene content + SNP	0.516 (0.036)	0.454	**0.955 (0.02)**	0.957	1.0 (0.0)	1.0	0.0 (0.0)	0.0	0.0 (0.0)	0.0

### Appendix E.2. N. gonorrhoeae

**Table A21:** *N. gonorrhoeae*–ceftriaxone.

	RMSE-CV	RMSE-H	DD1-CV	DD1-H	DD2-CV	DD2-H	ME-CV	ME-H	VME-CV	VME-H
NT 8-mers	0.87 (0.08)	0.59	0.812 (0.026)	0.927	0.959 (0.016)	0.979	0.0 (0.0)	0.0	0.0 (0.0)	0.0
NT 9-mers	0.839 (0.074)	0.538	0.818 (0.031)	0.932	0.968 (0.014)	0.995	0.0 (0.0)	0.0	0.0 (0.0)	0.0
NT 10-mers	0.846 (0.076)	0.604	0.823 (0.019)	0.932	0.966 (0.017)	0.990	0.0 (0.0)	0.0	0.0 (0.0)	0.0
NT 11-mers	0.83 (0.068)	0.571	0.814 (0.017)	0.916	0.97 (0.013)	0.990	0.0 (0.0)	0.0	0.0 (0.0)	0.0
AA 3-mers	0.891 (0.046)	0.492	0.799 (0.026)	0.921	0.957 (0.009)	0.990	0.0 (0.0)	0.0	0.0 (0.0)	0.0
AA 4-mers	0.84 (0.075)	0.596	0.812 (0.033)	0.927	0.966 (0.01)	0.984	0.0 (0.0)	0.0	0.0 (0.0)	0.0
AA 5-mers	**0.796 (0.067)**	**0.463**	**0.835 (0.023)**	**0.963**	**0.974 (0.012)**	0.995	0.0 (0.0)	0.0	0.0 (0.0)	0.0
Gene content	0.873 (0.068)	0.683	0.797 (0.024)	0.895	0.961 (0.015)	0.984	0.0 (0.0)	0.0	0.0 (0.0)	0.0
SNP	0.852 (0.071)	0.673	0.812 (0.031)	0.843	0.963 (0.018)	0.995	0.0 (0.0)	0.0	0.0 (0.0)	0.0
Gene + SNP	0.86 (0.075)	0.59	0.801 (0.033)	0.916	0.965 (0.015)	0.995	0.0 (0.0)	0.0	0.0 (0.0)	0.0

**Table A22:** *N. gonorrhoeae*–tetracycline.

	RMSE-CV	RMSE-H	DD1-CV	DD1-H	DD2-CV	DD2-H	ME-CV	ME-H	VME-CV	VME-H
NT 8-mers	1.001 (0.141)	0.774	0.771 (0.052)	0.812	0.944 (0.018)	0.971	0.006 (0.008)	0.000	0.0 (0.0)	0.0
NT 9-mers	0.979 (0.142)	0.824	0.785 (0.053)	0.783	0.939 (0.032)	0.986	0.008 (0.008)	0.000	0.0 (0.0)	0.0
NT 10-mers	0.963 (0.131)	0.694	**0.794 (0.042)**	**0.913**	0.939 (0.032)	0.971	0.005 (0.007)	0.000	0.0 (0.0)	0.0
NT 11-mers	0.967 (0.112)	0.905	0.787 (0.023)	0.812	0.942 (0.029)	0.928	0.005 (0.007)	0.000	0.0 (0.0)	0.0
AA 3-mers	0.999 (0.134)	0.861	0.787 (0.051)	0.841	0.941 (0.029)	0.928	0.005 (0.007)	0.000	0.0 (0.0)	0.0
AA 4-mers	1.029 (0.063)	0.834	0.757 (0.026)	0.87	0.936 (0.02)	0.928	0.005 (0.007)	0.000	0.0 (0.0)	0.0
AA 5-mers	0.971 (0.116)	0.838	0.791 (0.039)	0.899	0.936 (0.02)	0.957	**0.003 (0.006)**	0.014	0.0 (0.0)	0.0
Gene content	0.979 (0.132)	**0.666**	0.768 (0.064)	0.899	0.947 (0.033)	0.986	0.005 (0.01)	0.000	0.0 (0.0)	0.0
SNP	1.034 (0.121)	0.679	0.784 (0.031)	0.884	0.926 (0.023)	0.986	0.01 (0.011)	0.000	0.0 (0.0)	0.0
Gene + SNP	**0.955 (0.107)**	0.724	0.779 (0.029)	0.87	**0.952 (0.026)**	0.971	0.005 (0.007)	0.000	0.0 (0.0)	0.0

**Table A23:** *N. gonorrhoeae*–erythromycin.

	RMSE-CV	RMSE-H	DD1-CV	DD1-H	DD2-CV	DD2-H	ME-CV	ME-H	VME-CV	VME-H
NT 8-mers	1.196 (0.383)	1.367	0.77 (0.115)	0.824	0.938 (0.048)	0.882	-	-	-	-
NT 9-mers	1.2 (0.426)	1.314	0.753 (0.135)	0.824	0.932 (0.051)	0.941	-	-	-	-
NT 10-mers	**1.166 (0.365)**	1.316	0.752 (0.1)	0.882	0.932 (0.043)	0.882	-	-	-	-
NT 11-mers	1.279 (0.362)	1.476	0.77 (0.098)	0.824	0.913 (0.064)	0.941	-	-	-	-
AA 3-mers	1.371 (0.428)	**0.356**	0.672 (0.1)	**0.941**	0.864 (0.06)	1.000	-	-	-	-
AA 4-mers	1.228 (0.311)	1.595	0.733 (0.055)	0.824	0.913 (0.03)	0.882	-	-	-	-
AA 5-mers	1.17 (0.35)	1.247	**0.776 (0.057)**	0.882	0.938 (0.038)	0.941	-	-	-	-
Gene content	1.263 (0.367)	1.008	0.739 (0.088)	0.706	0.913 (0.041)	0.941	-	-	-	-
SNP	1.226 (0.402)	0.669	0.752 (0.081)	0.824	0.926 (0.067)	1.000	-	-	-	-
Gene + SNP	1.277 (0.395)	0.846	0.764 (0.061)	0.882	0.913 (0.057)	0.941	-	-	-	-

**Table A24:** *N. gonorrhoeae*–cefpodoxime.

	RMSE-CV	RMSE-H	DD1-CV	DD1-H	DD2-CV	DD2-H	ME-CV	ME-H	VME-CV	VME-H
NT 8-mers	1.731 (0.328)	1.064	0.597 (0.11)	0.75	0.827 (0.062)	0.75	0.0 (0.0)	0.0	0.0 (0.0)	0.0
NT 9-mers	1.699 (0.283)	**0.607**	0.598 (0.099)	0.75	0.831 (0.077)	1.00	0.0 (0.0)	0.0	0.0 (0.0)	0.0
NT 10-mers	1.785 (0.283)	1.037	0.542 (0.092)	0.75	0.766 (0.079)	1.00	0.0 (0.0)	0.0	0.0 (0.0)	0.0
NT 11-mers	1.698 (0.318)	1.2	**0.623 (0.102)**	0.50	0.809 (0.094)	0.75	0.0 (0.0)	0.0	0.0 (0.0)	0.0
AA 3-mers	1.654 (0.307)	1.558	0.572 (0.117)	0.50	0.814 (0.056)	0.75	0.0 (0.0)	0.0	0.0 (0.0)	0.0
AA 4-mers	1.625 (0.286)	0.841	0.615 (0.073)	0.75	0.823 (0.052)	1.00	0.0 (0.0)	0.0	0.0 (0.0)	0.0
AA 5-mers	1.734 (0.261)	1.402	0.593 (0.097)	0.75	0.813 (0.059)	0.75	0.0 (0.0)	0.0	0.0 (0.0)	0.0
Gene content	1.722 (0.281)	0.903	0.528 (0.088)	0.75	0.783 (0.065)	1.00	0.0 (0.0)	0.0	0.0 (0.0)	0.0
SNP	1.662 (0.319)	1.086	0.615 (0.086)	0.25	0.827 (0.051)	1.00	0.0 (0.0)	0.0	0.0 (0.0)	0.0
Gene + SNP	**1.618 (0.34)**	1.126	0.602 (0.118)	0.75	0.831 (0.069)	0.75	0.0 (0.0)	0.0	0.0 (0.0)	0.0

**Table A25:** *N. gonorrhoeae*–spectinomycin.

	RMSE-CV	RMSE-H	DD1-CV	DD1-H	DD2-CV	DD2-H	ME-CV	ME-H	VME-CV	VME-H
NT 8-mers	0.352 (0.082)	**0.082**	0.985 (0.016)	1.000	0.995 (0.008)	1.0	0.0 (0.0)	0.0	0.0 (0.0)	0.0
NT 9-mers	0.337 (0.101)	0.109	0.986 (0.013)	1.000	0.995 (0.011)	1.0	0.0 (0.0)	0.0	0.0 (0.0)	0.0
NT 10-mers	**0.305 (0.049)**	0.161	**0.995 (0.011)**	1.000	**0.998 (0.005)**	1.0	0.0 (0.0)	0.0	0.0 (0.0)	0.0
NT 11-mers	0.323 (0.057)	0.148	0.983 (0.013)	1.000	0.995 (0.008)	1.0	0.0 (0.0)	0.0	0.0 (0.0)	0.0
AA 3-mers	0.38 (0.117)	0.109	0.973 (0.022)	1.000	0.995 (0.008)	1.0	0.0 (0.0)	0.0	0.0 (0.0)	0.0
AA 4-mers	0.332 (0.088)	0.227	0.98 (0.021)	0.985	0.997 (0.007)	1.0	0.0 (0.0)	0.0	0.0 (0.0)	0.0
AA 5-mers	0.332 (0.137)	0.154	0.988 (0.015)	1.000	0.997 (0.01)	1.0	0.0 (0.0)	0.0	0.0 (0.0)	0.0
Gene content	0.36 (0.08)	0.177	0.98 (0.015)	1.000	0.997 (0.007)	1.0	0.0 (0.0)	0.0	0.0 (0.0)	0.0
SNP	0.722 (0.183)	0.224	0.889 (0.033)	1.000	0.968 (0.025)	1.0	0.0 (0.0)	0.0	0.0 (0.0)	0.0
Gene + SNP	0.402 (0.119)	0.153	0.961 (0.022)	1.000	0.997 (0.007)	1.0	0.0 (0.0)	0.0	0.0 (0.0)	0.0

**Table A26:** *N. gonorrhoeae*–cefixime.

	RMSE-CV	RMSE-H	DD1-CV	DD1-H	DD2-CV	DD2-H	ME-CV	ME-H	VME-CV	VME-H
NT 8-mers	0.94 (0.076)	0.637	0.81 (0.025)	0.932	0.952 (0.014)	0.989	0.0 (0.0)	0.0	0.0 (0.0)	0.0
NT 9-mers	0.944 (0.122)	0.617	0.808 (0.029)	0.958	0.953 (0.016)	0.979	0.0 (0.0)	0.0	0.0 (0.0)	0.0
NT 10-mers	0.959 (0.069)	0.635	0.82 (0.027)	0.932	0.949 (0.011)	0.974	0.0 (0.0)	0.0	0.0 (0.0)	0.0
NT 11-mers	0.91 (0.105)	0.537	0.825 (0.03)	0.932	0.956 (0.018)	**0.995**	0.0 (0.0)	0.0	0.0 (0.0)	0.0
AA 3-mers	0.981 (0.12)	**0.456**	0.809 (0.031)	0.958	0.938 (0.02)	0.984	0.0 (0.0)	0.0	0.0 (0.0)	0.0
AA 4-mers	**0.895 (0.09)**	0.62	**0.839 (0.029)**	0.937	0.953 (0.014)	0.979	0.0 (0.0)	0.0	0.0 (0.0)	0.0
AA 5-mers	0.938 (0.098)	0.535	0.819 (0.039)	0.937	0.951 (0.015)	0.979	0.0 (0.0)	0.0	0.0 (0.0)	0.0
Gene content	0.902 (0.084)	0.754	0.821 (0.031)	0.868	**0.961 (0.014)**	0.974	0.0 (0.0)	0.0	0.0 (0.0)	0.0
SNP	0.943 (0.11)	0.689	0.804 (0.036)	0.879	0.953 (0.021)	0.989	0.0 (0.0)	0.0	0.0 (0.0)	0.0
Gene + SNP	0.916 (0.078)	0.66	0.811 (0.027)	0.916	0.957 (0.011)	0.968	0.0 (0.0)	0.0	0.0 (0.0)	0.0

**Table A27:** *N. gonorrhoeae*–cefpodoximeproxetil.

	RMSE-CV	RMSE-H	DD1-CV	DD1-H	DD2-CV	DD2-H	ME-CV	ME-H	VME-CV	VME-H
NT 8-mers	1.146 (0.268)	0.096	0.745 (0.079)	1.000	**0.935 (0.039)**	1.0	0.0 (0.0)	0.0	0.0 (0.0)	0.0
NT 9-mers	1.162 (0.196)	0.096	0.754 (0.049)	1.000	0.922 (0.038)	1.0	0.0 (0.0)	0.0	0.0 (0.0)	0.0
NT 10-mers	1.119 (0.26)	0.174	0.758 (0.099)	1.000	0.913 (0.055)	1.0	0.0 (0.0)	0.0	0.0 (0.0)	0.0
NT 11-mers	1.148 (0.236)	0.849	0.75 (0.073)	0.667	0.922 (0.043)	1.0	0.0 (0.0)	0.0	0.0 (0.0)	0.0
AA 3-mers	1.143 (0.256)	0.194	0.736 (0.085)	1.000	0.914 (0.073)	1.0	0.0 (0.0)	0.0	0.0 (0.0)	0.0
AA 4-mers	1.143 (0.282)	0.143	0.732 (0.084)	1.000	0.913 (0.062)	1.0	0.0 (0.0)	0.0	0.0 (0.0)	0.0
AA 5-mers	1.117 (0.29)	0.230	0.758 (0.092)	1.000	0.922 (0.054)	1.0	0.0 (0.0)	0.0	0.0 (0.0)	0.0
Gene content	1.122 (0.278)	0.210	0.71 (0.108)	1.000	0.918 (0.05)	1.0	0.0 (0.0)	0.0	0.0 (0.0)	0.0
SNP	**1.088 (0.267)**	0.180	**0.784 (0.059)**	1.000	0.913 (0.034)	1.0	0.0 (0.0)	0.0	0.0 (0.0)	0.0
Gene + SNP	1.104 (0.296)	0.162	0.766 (0.079)	1.000	0.931 (0.056)	1.0	0.0 (0.0)	0.0	0.0 (0.0)	0.0

**Table A28:** *N. gonorrhoeae*–penicillin.

	RMSE-CV	RMSE-H	DD1-CV	DD1-H	DD2-CV	DD2-H	ME-CV	ME-H	VME-CV	VME-H
NT 8-mers	0.867 (0.093)	0.648	0.848 (0.043)	0.923	0.947 (0.019)	0.985	0.0 (0.0)	0.0	0.0 (0.0)	0.0
NT 9-mers	0.903 (0.108)	0.63	0.826 (0.034)	0.954	0.944 (0.022)	0.985	0.0 (0.0)	0.0	0.0 (0.0)	0.0
NT 10-mers	0.899 (0.125)	0.583	0.829 (0.048)	0.892	0.944 (0.025)	1.000	0.0 (0.0)	0.0	0.0 (0.0)	0.0
NT 11-mers	0.894 (0.133)	0.725	0.851 (0.057)	0.846	0.945 (0.02)	0.985	0.0 (0.0)	0.0	0.0 (0.0)	0.0
AA 3-mers	0.886 (0.133)	0.59	0.836 (0.041)	0.923	0.949 (0.026)	0.954	0.0 (0.0)	0.0	0.0 (0.0)	0.0
AA 4-mers	0.83 (0.124)	0.552	0.838 (0.064)	0.908	0.956 (0.031)	0.969	0.0 (0.0)	0.0	0.0 (0.0)	0.0
AA 5-mers	**0.754 (0.112)**	**0.529**	**0.855 (0.059)**	0.954	**0.969 (0.017)**	0.969	0.0 (0.0)	0.0	0.0 (0.0)	0.0
Gene content	0.876 (0.102)	0.61	0.821 (0.05)	0.908	0.957 (0.035)	1.000	0.0 (0.0)	0.0	0.0 (0.0)	0.0
SNP	0.923 (0.176)	0.846	0.828 (0.046)	0.877	0.942 (0.031)	0.938	0.0 (0.0)	0.0	0.0 (0.0)	0.0
Gene + SNP	0.854 (0.149)	0.788	0.836 (0.047)	0.769	0.959 (0.028)	1.000	0.0 (0.0)	0.0	0.0 (0.0)	0.0

**Table A29:** *N. gonorrhoeae*–azithromycin.

	RMSE-CV	RMSE-H	DD1-CV	DD1-H	DD2-CV	DD2-H	ME-CV	ME-H	VME-CV	VME-H
NT 8-mers	1.271 (0.175)	1.229	0.771 (0.052)	0.911	0.906 (0.021)	0.933	0.0 (0.0)	0.0	0.0 (0.0)	0.0
NT 9-mers	1.146 (0.226)	0.815	0.793 (0.05)	0.911	0.928 (0.018)	0.967	0.0 (0.0)	0.0	0.0 (0.0)	0.0
NT 10-mers	1.093 (0.273)	0.744	0.818 (0.046)	0.944	0.935 (0.033)	0.978	0.0 (0.0)	0.0	0.0 (0.0)	0.0
NT 11-mers	**1.064 (0.202)**	0.636	**0.826 (0.041)**	0.889	**0.944 (0.025)**	0.989	0.0 (0.0)	0.0	0.0 (0.0)	0.0
AA 3-mers	1.31 (0.203)	0.829	0.764 (0.028)	0.900	0.912 (0.022)	0.956	0.0 (0.0)	0.0	0.0 (0.0)	0.0
AA 4-mers	1.195 (0.239)	0.747	0.789 (0.041)	0.922	0.913 (0.027)	0.989	0.0 (0.0)	0.0	0.0 (0.0)	0.0
AA 5-mers	1.245 (0.251)	**0.414**	0.802 (0.046)	0.944	0.92 (0.024)	**1.0**	0.0 (0.0)	0.0	0.0 (0.0)	0.0
Gene content	1.225 (0.152)	0.821	0.745 (0.02)	0.867	0.911 (0.017)	0.967	0.0 (0.0)	0.0	0.0 (0.0)	0.0
SNP	1.39 (0.161)	0.712	0.718 (0.042)	0.911	0.891 (0.017)	0.978	0.0 (0.0)	0.0	0.0 (0.0)	0.0
Gene + SNP	1.241 (0.214)	0.77	0.753 (0.038)	0.944	0.912 (0.026)	0.978	0.0 (0.0)	0.0	0.0 (0.0)	0.0

**Table A30:** *N. gonorrhoeae*–ciprofloxacin.

	RMSE-CV	RMSE-H	DD1-CV	DD1-H	DD2-CV	DD2-H	ME-CV	ME-H	VME-CV	VME-H
NT 8-mers	1.691 (0.594)	1.44	0.807 (0.047)	0.921	0.902 (0.05)	0.984	0.012 (0.018)	0.000	0.01 (0.009)	0.016
NT 9-mers	**1.4 (0.565)**	**0.448**	0.84 (0.043)	0.937	0.941 (0.031)	1.000	0.01 (0.012)	0.000	0.007 (0.009)	0.000
NT 10-mers	1.521 (0.5)	0.547	0.854 (0.051)	0.905	0.944 (0.023)	0.968	0.01 (0.012)	0.000	0.01 (0.012)	0.000
NT 11-mers	1.563 (0.548)	1.696	0.847 (0.049)	0.857	0.939 (0.032)	0.905	0.012 (0.008)	0.032	0.012 (0.014)	0.000
AA 3-mers	1.987 (0.836)	1.526	0.828 (0.052)	0.952	0.892 (0.052)	0.968	0.016 (0.018)	0.000	0.019 (0.02)	0.016
AA 4-mers	1.403 (0.775)	1.424	0.858 (0.033)	0.952	0.939 (0.038)	0.984	0.009 (0.012)	0.000	0.009 (0.014)	0.016
AA 5-mers	1.665 (0.698)	1.235	**0.885 (0.033)**	**0.968**	**0.953 (0.036)**	0.984	0.017 (0.019)	0.000	0.012 (0.011)	0.016
Gene content	1.772 (0.567)	0.484	0.802 (0.048)	0.937	0.91 (0.032)	1.000	0.017 (0.017)	0.000	0.014 (0.017)	0.000
SNP	1.54 (0.507)	1.622	0.828 (0.03)	0.889	0.929 (0.028)	0.952	0.01 (0.012)	0.000	**0.004 (0.007)**	0.016
Gene + SNP	1.504 (0.422)	0.745	0.838 (0.034)	0.937	0.934 (0.03)	0.968	0.009 (0.016)	0.000	0.007 (0.012)	0.000

### Appendix E.3. K. pneumoniae

**Table A31:** *K. pneumoniae*–aztreonam.

	RMSE-CV	RMSE-H	DD1-CV	DD1-H	DD2-CV	DD2-H	ME-CV	ME-H	VME-CV	VME-H
NT 8-mers	1.128 (0.056)	1.258	0.77 (0.017)	0.787	0.903 (0.014)	0.878	0.046 (0.008)	0.067	0.007 (0.005)	0.012
NT 9-mers	1.093 (0.081)	1.22	0.787 (0.017)	0.762	0.91 (0.015)	0.866	0.045 (0.006)	0.067	0.005 (0.004)	0.006
NT 10-mers	1.057 (0.062)	1.135	0.805 (0.026)	0.774	0.914 (0.015)	0.896	0.043 (0.008)	0.049	0.007 (0.006)	0.006
NT 11-mers	1.046 (0.08)	**1.064**	0.819 (0.019)	0.805	0.915 (0.017)	0.902	0.043 (0.01)	0.049	0.008 (0.009)	0.006
AA 3-mers	1.129 (0.047)	1.175	0.782 (0.018)	0.774	0.896 (0.021)	0.884	0.048 (0.01)	0.055	0.005 (0.005)	0.012
AA 4-mers	1.076 (0.081)	1.161	0.801 (0.024)	0.756	0.911 (0.012)	0.890	0.043 (0.011)	0.061	0.005 (0.006)	0.006
AA 5-mers	**1.022 (0.082)**	1.145	**0.834 (0.024)**	0.793	**0.922 (0.017)**	0.902	0.043 (0.014)	0.055	0.005 (0.004)	0.006
Gene content	1.037 (0.045)	1.145	0.816 (0.021)	**0.811**	0.918 (0.009)	0.884	0.043 (0.014)	0.061	**0.003 (0.003)**	0.006
SNP	1.124 (0.085)	1.199	0.798 (0.026)	0.774	0.898 (0.016)	0.884	0.047 (0.008)	0.055	0.006 (0.008)	0.006
Gene + SNP	1.106 (0.073)	1.177	0.803 (0.024)	0.787	0.901 (0.015)	0.902	0.046 (0.011)	0.079	0.008 (0.009)	**0.0**

**Table A32:** *K. pneumoniae*–cefoxitin.

	RMSE-CV	RMSE-H	DD1-CV	DD1-H	DD2-CV	DD2-H	ME-CV	ME-H	VME-CV	VME-H
NT 8-mers	0.889 (0.049)	0.907	0.766 (0.026)	0.750	0.951 (0.017)	0.945	0.001 (0.004)	0.006	0.032 (0.012)	0.037
NT 9-mers	0.894 (0.043)	0.938	0.776 (0.026)	0.756	0.948 (0.013)	0.927	0.001 (0.002)	0.000	0.04 (0.01)	0.049
NT 10-mers	0.887 (0.061)	0.918	0.773 (0.031)	0.762	0.949 (0.017)	0.945	0.001 (0.002)	0.000	0.034 (0.009)	0.037
NT 11-mers	0.9 (0.051)	**0.865**	0.778 (0.024)	0.774	0.945 (0.014)	0.951	0.001 (0.002)	0.000	0.037 (0.011)	0.043
AA 3-mers	0.898 (0.039)	0.976	0.758 (0.023)	0.720	0.956 (0.014)	0.945	0.001 (0.002)	0.000	**0.028 (0.013)**	0.037
AA 4-mers	0.865 (0.054)	0.91	0.785 (0.028)	0.750	0.955 (0.015)	0.951	0.0 (0.0)	0.000	0.029 (0.01)	0.037
AA 5-mers	**0.854 (0.053)**	0.887	**0.788 (0.024)**	0.744	0.955 (0.011)	0.951	0.001 (0.002)	0.000	0.034 (0.013)	0.043
Gene content	0.87 (0.047)	0.866	0.766 (0.02)	0.774	0.959 (0.016)	**0.963**	0.0 (0.0)	0.000	0.03 (0.013)	**0.018**
SNP	0.885 (0.057)	0.93	0.765 (0.034)	0.768	0.959 (0.015)	0.927	0.001 (0.002)	0.000	0.032 (0.01)	0.049
Gene + SNP	0.87 (0.065)	0.905	0.77 (0.032)	0.756	0.955 (0.016)	0.951	0.001 (0.003)	0.000	0.03 (0.009)	0.037

**Table A33:** *K. pneumoniae*–meropenem.

	RMSE-CV	RMSE-H	DD1-CV	DD1-H	DD2-CV	DD2-H	ME-CV	ME-H	VME-CV	VME-H
NT 8-mers	0.934 (0.094)	**0.44**	0.84 (0.024)	**0.976**	0.948 (0.01)	**0.994**	**0.011 (0.009)**	0.000	0.004 (0.004)	0.000
NT 9-mers	0.849 (0.128)	0.564	0.867 (0.028)	0.958	0.95 (0.015)	0.982	0.012 (0.008)	0.006	0.004 (0.004)	0.006
NT 10-mers	0.846 (0.096)	0.627	0.87 (0.024)	0.964	0.951 (0.01)	0.976	0.013 (0.008)	0.012	0.003 (0.003)	0.006
NT 11-mers	0.824 (0.126)	0.528	0.873 (0.021)	0.97	0.952 (0.018)	0.988	0.012 (0.009)	0.006	0.003 (0.003)	0.006
AA 3-mers	0.996 (0.101)	0.527	0.803 (0.034)	0.952	0.933 (0.021)	0.988	0.016 (0.01)	0.000	0.01 (0.006)	0.012
AA 4-mers	0.868 (0.098)	0.529	0.869 (0.021)	0.97	0.948 (0.014)	0.982	0.015 (0.007)	0.012	0.007 (0.006)	0.000
AA 5-mers	**0.794 (0.122)**	0.527	**0.882 (0.023)**	0.958	**0.956 (0.021)**	0.988	0.015 (0.009)	0.006	0.004 (0.004)	0.006
Gene content	0.845 (0.125)	0.634	0.854 (0.03)	0.945	0.95 (0.018)	0.982	0.015 (0.011)	0.000	0.005 (0.004)	0.012
SNP	1.18 (0.109)	0.85	0.762 (0.024)	0.879	0.892 (0.02)	0.945	0.026 (0.009)	0.012	0.009 (0.005)	0.006
Gene + SNP	0.889 (0.114)	0.594	0.861 (0.031)	0.952	0.945 (0.021)	0.988	0.02 (0.011)	0.000	0.006 (0.005)	0.012

**Table A34:** *K. pneumoniae*–tobramycin.

	RMSE-CV	RMSE-H	DD1-CV	DD1-H	DD2-CV	DD2-H	ME-CV	ME-H	VME-CV	VME-H
NT 8-mers	0.841 (0.075)	0.925	0.795 (0.046)	0.753	0.965 (0.012)	0.934	0.0 (0.0)	0.0	0.015 (0.008)	0.054
NT 9-mers	0.73 (0.079)	0.748	0.861 (0.03)	0.843	0.972 (0.015)	0.97	0.0 (0.0)	0.0	0.015 (0.009)	0.024
NT 10-mers	0.669 (0.079)	0.693	0.897 (0.025)	0.91	0.976 (0.012)	0.952	0.0 (0.0)	0.0	0.015 (0.008)	0.042
NT 11-mers	0.644 (0.08)	0.698	0.89 (0.027)	**0.916**	0.981 (0.011)	0.97	0.0 (0.0)	0.0	0.011 (0.007)	0.024
AA 3-mers	0.808 (0.046)	0.802	0.803 (0.026)	0.801	0.971 (0.011)	0.976	0.0 (0.0)	0.0	0.013 (0.008)	0.018
AA 4-mers	0.667 (0.076)	0.739	0.889 (0.033)	0.861	0.976 (0.008)	0.976	0.0 (0.0)	0.0	0.014 (0.011)	0.018
AA 5-mers	0.641 (0.055)	0.671	0.904 (0.027)	0.873	0.981 (0.007)	**0.982**	0.0 (0.0)	0.0	**0.01 (0.006)**	**0.012**
Gene content	0.649 (0.063)	**0.668**	0.9 (0.021)	0.904	**0.982 (0.009)**	0.976	0.0 (0.0)	0.0	0.012 (0.007)	0.018
SNP	0.856 (0.089)	0.891	0.797 (0.037)	0.801	0.953 (0.019)	0.964	0.001 (0.002)	0.0	0.023 (0.014)	0.024
Gene + SNP	**0.625 (0.081)**	0.7	**0.909 (0.017)**	0.886	0.979 (0.013)	0.97	0.0 (0.0)	0.0	0.012 (0.008)	0.024

**Table A35:** *K. pneumoniae*–gentamicin.

	RMSE-CV	RMSE-H	DD1-CV	DD1-H	DD2-CV	DD2-H	ME-CV	ME-H	VME-CV	VME-H
NT 8-mers	0.879 (0.043)	0.812	0.799 (0.02)	0.813	0.943 (0.011)	0.952	0.001 (0.002)	0.000	0.038 (0.01)	0.042
NT 9-mers	0.781 (0.055)	0.762	0.847 (0.027)	0.849	0.958 (0.015)	0.964	0.001 (0.002)	0.000	0.027 (0.011)	0.030
NT 10-mers	0.676 (0.068)	0.585	0.909 (0.021)	0.922	0.966 (0.01)	0.982	0.002 (0.003)	0.000	0.022 (0.006)	0.018
NT 11-mers	0.629 (0.053)	**0.546**	0.912 (0.018)	0.922	0.973 (0.01)	0.982	0.001 (0.002)	0.000	0.019 (0.006)	0.018
AA 3-mers	0.869 (0.07)	0.851	0.789 (0.028)	0.765	0.951 (0.023)	0.964	0.001 (0.003)	0.006	0.028 (0.014)	0.018
AA 4-mers	0.664 (0.075)	0.646	0.905 (0.022)	0.880	0.973 (0.012)	0.982	0.001 (0.003)	0.000	0.017 (0.004)	0.012
AA 5-mers	**0.608 (0.081)**	0.57	0.924 (0.026)	0.910	0.975 (0.01)	0.988	0.001 (0.003)	0.000	**0.015 (0.007)**	0.012
Gene content	0.634 (0.044)	0.557	0.913 (0.017)	0.922	0.975 (0.011)	0.982	0.001 (0.002)	0.000	0.019 (0.007)	0.018
SNP	0.766 (0.075)	0.657	0.873 (0.027)	0.916	0.951 (0.018)	0.964	0.001 (0.003)	0.000	0.033 (0.012)	0.030
Gene + SNP	0.636 (0.039)	0.587	**0.926 (0.011)**	0.904	0.972 (0.008)	0.988	**0.0 (0.0)**	0.000	0.021 (0.007)	0.012

**Table A36:** *K. pneumoniae*–imipenem.

	RMSE-CV	RMSE-H	DD1-CV	DD1-H	DD2-CV	DD2-H	ME-CV	ME-H	VME-CV	VME-H
NT 8-mers	0.91 (0.131)	0.758	0.847 (0.037)	0.892	0.94 (0.024)	0.97	0.019 (0.014)	0.006	0.005 (0.004)	0.000
NT 9-mers	0.845 (0.095)	0.773	0.865 (0.019)	0.904	0.949 (0.019)	0.964	0.019 (0.01)	0.012	0.005 (0.008)	0.000
NT 10-mers	0.831 (0.098)	0.725	0.877 (0.026)	0.898	0.954 (0.018)	0.958	0.019 (0.011)	0.006	**0.002 (0.004)**	0.000
NT 11-mers	0.819 (0.099)	0.771	**0.886 (0.027)**	0.916	0.951 (0.023)	0.97	0.019 (0.013)	0.012	0.005 (0.007)	0.000
AA 3-mers	0.97 (0.099)	0.74	0.824 (0.032)	0.898	0.933 (0.022)	0.97	0.02 (0.015)	**0.0**	0.009 (0.007)	0.000
AA 4-mers	0.827 (0.099)	**0.63**	0.883 (0.024)	0.916	0.953 (0.017)	**0.982**	0.021 (0.011)	0.006	0.005 (0.004)	0.006
AA 5-mers	**0.799 (0.104)**	0.751	0.878 (0.027)	**0.922**	**0.958 (0.02)**	0.964	0.019 (0.014)	0.012	0.005 (0.006)	0.006
Gene content	0.825 (0.084)	0.708	0.874 (0.029)	0.892	0.956 (0.017)	0.97	0.021 (0.012)	0.018	0.003 (0.004)	0.000
SNP	1.053 (0.087)	0.93	0.789 (0.03)	0.801	0.921 (0.021)	0.964	0.034 (0.016)	0.018	0.006 (0.007)	0.012
Gene + SNP	0.82 (0.105)	0.78	0.872 (0.026)	0.916	0.956 (0.02)	0.964	0.02 (0.012)	0.018	0.006 (0.008)	0.006

**Table A37:** *K. pneumoniae*–levofloxacin.

	RMSE-CV	RMSE-H	DD1-CV	DD1-H	DD2-CV	DD2-H	ME-CV	ME-H	VME-CV	VME-H
NT 8-mers	0.584 (0.108)	0.599	0.905 (0.027)	0.898	0.977 (0.02)	0.988	0.0 (0.0)	0.0	0.0 (0.0)	0.0
NT 9-mers	0.57 (0.077)	0.539	0.907 (0.021)	0.916	0.979 (0.014)	0.988	0.0 (0.0)	0.0	0.0 (0.0)	0.0
NT 10-mers	0.553 (0.093)	0.627	0.927 (0.02)	0.880	0.979 (0.014)	0.976	0.0 (0.0)	0.0	0.0 (0.0)	0.0
NT 11-mers	0.523 (0.113)	**0.494**	0.932 (0.019)	0.934	0.977 (0.018)	0.982	0.0 (0.0)	0.0	0.0 (0.0)	0.0
AA 3-mers	0.613 (0.07)	0.605	0.901 (0.018)	0.886	0.976 (0.015)	0.976	0.0 (0.0)	0.0	0.0 (0.0)	0.0
AA 4-mers	0.559 (0.068)	0.656	0.92 (0.014)	0.880	0.978 (0.01)	0.970	0.0 (0.0)	0.0	0.0 (0.0)	0.0
AA 5-mers	**0.51 (0.086)**	0.548	0.937 (0.021)	0.928	0.979 (0.011)	0.970	0.0 (0.0)	0.0	0.0 (0.0)	0.0
Gene content	0.56 (0.06)	0.553	0.918 (0.018)	0.940	0.98 (0.012)	0.976	0.0 (0.0)	0.0	0.0 (0.0)	0.0
SNP	0.549 (0.06)	0.576	0.929 (0.017)	0.910	0.977 (0.009)	0.970	0.0 (0.0)	0.0	0.0 (0.0)	0.0
Gene + SNP	0.514 (0.113)	0.538	**0.938 (0.022)**	0.940	0.98 (0.014)	0.970	0.0 (0.0)	0.0	0.0 (0.0)	0.0

**Table A38:** *K. pneumoniae*–nitrofurantoin.

	RMSE-CV	RMSE-H	DD1-CV	DD1-H	DD2-CV	DD2-H	ME-CV	ME-H	VME-CV	VME-H
NT 8-mers	0.575 (0.069)	0.504	0.929 (0.019)	0.933	0.985 (0.007)	1.000	0.0 (0.0)	0.0	0.004 (0.006)	0.000
NT 9-mers	0.58 (0.065)	0.516	0.922 (0.014)	0.933	0.986 (0.007)	1.000	0.0 (0.0)	0.0	0.002 (0.005)	0.000
NT 10-mers	0.613 (0.075)	0.646	0.921 (0.027)	0.91	0.979 (0.011)	0.989	0.0 (0.0)	0.0	0.007 (0.01)	0.011
NT 11-mers	0.576 (0.046)	0.581	0.928 (0.015)	0.933	0.984 (0.008)	0.989	0.0 (0.0)	0.0	0.005 (0.008)	0.011
AA 3-mers	0.58 (0.037)	0.531	0.927 (0.019)	0.921	0.984 (0.008)	1.000	0.001 (0.004)	0.0	0.002 (0.005)	0.000
AA 4-mers	0.565 (0.051)	**0.46**	0.932 (0.027)	**0.966**	0.983 (0.006)	1.000	0.001 (0.004)	0.0	0.002 (0.005)	0.000
AA 5-mers	0.535 (0.044)	0.469	**0.944 (0.02)**	0.944	0.988 (0.008)	1.000	0.0 (0.0)	0.0	0.001 (0.004)	0.000
Gene content	0.558 (0.044)	0.482	0.933 (0.016)	0.955	0.985 (0.007)	1.000	0.0 (0.0)	0.0	0.001 (0.004)	0.000
SNP	**0.528 (0.055)**	0.549	0.943 (0.02)	0.921	**0.989 (0.007)**	1.000	0.0 (0.0)	0.0	0.001 (0.004)	0.000
Gene + SNP	0.539 (0.066)	0.555	0.94 (0.015)	0.91	0.988 (0.01)	1.000	0.0 (0.0)	0.0	0.001 (0.004)	0.000

**Table A39:** *K. pneumoniae*–ampicillin.

	RMSE-CV	RMSE-H	DD1-CV	DD1-H	DD2-CV	DD2-H	ME-CV	ME-H	VME-CV	VME-H
NT 8-mers	0.111 (0.013)	0.084	1.0 (0.0)	1.0	1.0 (0.0)	1.0	0.0 (0.0)	0.0	0.0 (0.0)	0.0
NT 9-mers	0.108 (0.012)	0.11	1.0 (0.0)	1.0	1.0 (0.0)	1.0	0.0 (0.0)	0.0	0.0 (0.0)	0.0
NT 10-mers	0.12 (0.008)	**0.083**	1.0 (0.0)	1.0	1.0 (0.0)	1.0	0.0 (0.0)	0.0	0.0 (0.0)	0.0
NT 11-mers	0.107 (0.012)	0.106	1.0 (0.0)	1.0	1.0 (0.0)	1.0	0.0 (0.0)	0.0	0.0 (0.0)	0.0
AA 3-mers	0.111 (0.014)	0.096	1.0 (0.0)	1.0	1.0 (0.0)	1.0	0.0 (0.0)	0.0	0.0 (0.0)	0.0
AA 4-mers	0.108 (0.007)	0.105	1.0 (0.0)	1.0	1.0 (0.0)	1.0	0.0 (0.0)	0.0	0.0 (0.0)	0.0
AA 5-mers	0.113 (0.009)	0.098	1.0 (0.0)	1.0	1.0 (0.0)	1.0	0.0 (0.0)	0.0	0.0 (0.0)	0.0
Gene content	0.107 (0.008)	0.118	1.0 (0.0)	1.0	1.0 (0.0)	1.0	0.0 (0.0)	0.0	0.0 (0.0)	0.0
SNP	0.115 (0.002)	0.143	1.0 (0.0)	1.0	1.0 (0.0)	1.0	0.0 (0.0)	0.0	0.0 (0.0)	0.0
Gene + SNP	0.109 (0.012)	0.104	1.0 (0.0)	1.0	1.0 (0.0)	1.0	0.0 (0.0)	0.0	0.0 (0.0)	0.0

**Table A40:** *K. pneumoniae*–tetracycline.

	RMSE-CV	RMSE-H	DD1-CV	DD1-H	DD2-CV	DD2-H	ME-CV	ME-H	VME-CV	VME-H
NT 8-mers	0.95 (0.042)	0.966	0.738 (0.039)	0.753	0.949 (0.012)	0.928	0.0 (0.0)	0.0	0.025 (0.01)	0.036
NT 9-mers	0.897 (0.053)	0.899	0.758 (0.04)	0.765	0.957 (0.01)	0.970	0.001 (0.002)	0.0	0.025 (0.009)	0.018
NT 10-mers	0.846 (0.043)	0.761	0.775 (0.032)	0.831	0.971 (0.009)	0.970	0.001 (0.003)	0.0	0.017 (0.01)	0.018
NT 11-mers	0.844 (0.056)	0.792	0.793 (0.037)	0.825	0.966 (0.01)	0.964	0.001 (0.003)	0.0	0.021 (0.009)	0.036
AA 3-mers	0.95 (0.044)	0.894	0.725 (0.036)	0.747	0.96 (0.015)	0.952	0.001 (0.003)	0.0	**0.011 (0.011)**	0.024
AA 4-mers	0.847 (0.04)	0.844	0.791 (0.041)	0.801	0.959 (0.008)	0.958	0.001 (0.003)	0.0	0.023 (0.006)	0.030
AA 5-mers	0.798 (0.047)	**0.722**	**0.804 (0.025)**	0.855	0.977 (0.01)	0.970	0.0 (0.0)	0.0	0.014 (0.01)	0.024
Gene content	**0.79 (0.048)**	0.795	0.794 (0.041)	0.837	0.977 (0.007)	0.970	0.001 (0.003)	0.0	0.014 (0.004)	0.030
SNP	1.022 (0.068)	0.997	0.69 (0.041)	0.729	0.949 (0.014)	0.946	0.001 (0.002)	0.0	0.015 (0.009)	0.030
Gene + SNP	0.812 (0.049)	0.785	0.793 (0.041)	**0.861**	0.969 (0.007)	0.964	0.001 (0.003)	0.0	0.021 (0.009)	0.024

**Table A41:** *K. pneumoniae*–ceftazidime.

	RMSE-CV	RMSE-H	DD1-CV	DD1-H	DD2-CV	DD2-H	ME-CV	ME-H	VME-CV	VME-H
NT 8-mers	1.016 (0.097)	0.955	0.855 (0.014)	0.825	0.929 (0.016)	0.916	0.017 (0.008)	0.018	0.008 (0.006)	0.000
NT 9-mers	0.975 (0.134)	0.861	0.863 (0.016)	0.843	0.937 (0.009)	0.94	0.011 (0.011)	0.012	0.008 (0.005)	0.000
NT 10-mers	0.953 (0.132)	0.719	0.871 (0.025)	0.873	0.935 (0.019)	0.964	0.007 (0.007)	0.018	0.012 (0.008)	0.000
NT 11-mers	0.906 (0.1)	0.834	0.869 (0.009)	0.825	0.948 (0.014)	0.946	0.006 (0.007)	0.012	0.007 (0.008)	0.000
AA 3-mers	1.1 (0.093)	0.928	0.839 (0.022)	0.843	0.921 (0.014)	0.928	0.017 (0.01)	0.012	0.008 (0.008)	0.000
AA 4-mers	0.977 (0.112)	0.778	0.865 (0.02)	0.867	0.938 (0.014)	0.952	0.01 (0.007)	0.012	0.01 (0.01)	0.000
AA 5-mers	**0.877 (0.122)**	0.712	0.885 (0.016)	0.88	**0.955 (0.014)**	**0.97**	0.006 (0.006)	0.018	0.011 (0.008)	0.000
Gene content	0.889 (0.112)	**0.706**	0.885 (0.017)	**0.904**	0.947 (0.017)	0.964	0.007 (0.006)	0.018	**0.005 (0.004)**	0.000
SNP	1.06 (0.101)	1.021	0.862 (0.017)	0.88	0.925 (0.014)	0.928	0.01 (0.006)	0.030	0.015 (0.008)	0.006
Gene + SNP	0.959 (0.105)	0.911	0.871 (0.014)	0.855	0.939 (0.015)	0.934	0.007 (0.008)	0.018	0.009 (0.006)	0.012

**Table A42:** *K. pneumoniae*–amikacin.

	RMSE-CV	RMSE-H	DD1-CV	DD1-H	DD2-CV	DD2-H	ME-CV	ME-H	VME-CV	VME-H
NT 8-mers	0.522 (0.045)	0.408	0.931 (0.02)	0.952	0.989 (0.007)	0.994	0.0 (0.0)	0.0	0.007 (0.005)	0.006
NT 9-mers	0.505 (0.057)	0.384	0.939 (0.014)	0.964	0.989 (0.009)	1.000	0.0 (0.0)	0.0	0.007 (0.005)	0.000
NT 10-mers	**0.476 (0.058)**	0.375	0.945 (0.015)	0.952	0.991 (0.007)	1.000	0.0 (0.0)	0.0	0.006 (0.006)	0.000
NT 11-mers	0.478 (0.063)	0.341	**0.946 (0.012)**	0.964	0.989 (0.008)	1.000	0.0 (0.0)	0.0	0.006 (0.005)	0.000
AA 3-mers	0.521 (0.07)	0.431	0.931 (0.022)	0.934	0.989 (0.01)	1.000	0.0 (0.0)	0.0	0.007 (0.005)	0.000
AA 4-mers	0.488 (0.07)	0.361	0.942 (0.014)	0.982	0.989 (0.009)	1.000	0.0 (0.0)	0.0	0.006 (0.006)	0.000
AA 5-mers	0.48 (0.063)	**0.323**	0.944 (0.019)	**0.988**	0.991 (0.006)	1.000	0.0 (0.0)	0.0	**0.005 (0.005)**	0.000
Gene content	0.5 (0.07)	0.369	0.942 (0.014)	0.964	0.987 (0.012)	1.000	0.0 (0.0)	0.0	0.007 (0.005)	0.000
SNP	0.549 (0.057)	0.455	0.934 (0.021)	0.958	0.986 (0.008)	0.994	0.0 (0.0)	0.0	0.008 (0.005)	0.006
Gene + SNP	0.494 (0.067)	0.363	0.945 (0.018)	0.97	0.989 (0.007)	0.994	0.0 (0.0)	0.0	0.007 (0.005)	0.006

**Table A43:** *K. pneumoniae*–ceftriaxone.

	RMSE-CV	RMSE-H	DD1-CV	DD1-H	DD2-CV	DD2-H	ME-CV	ME-H	VME-CV	VME-H
NT 8-mers	1.139 (0.137)	0.73	0.83 (0.021)	0.873	0.913 (0.021)	0.97	0.012 (0.008)	0.000	0.0 (0.0)	0.0
NT 9-mers	0.999 (0.125)	0.682	0.857 (0.019)	0.904	0.929 (0.017)	0.958	0.009 (0.008)	0.006	0.001 (0.002)	0.0
NT 10-mers	0.891 (0.131)	0.68	0.875 (0.016)	0.892	0.949 (0.014)	0.958	0.006 (0.006)	0.000	0.001 (0.003)	0.0
NT 11-mers	0.862 (0.129)	0.789	0.873 (0.026)	0.873	0.947 (0.019)	0.946	0.003 (0.004)	0.006	0.0 (0.0)	0.0
AA 3-mers	1.136 (0.112)	0.92	0.839 (0.008)	0.867	0.909 (0.013)	0.958	0.016 (0.01)	0.012	0.0 (0.0)	0.0
AA 4-mers	0.937 (0.2)	0.674	0.88 (0.025)	0.898	0.946 (0.023)	0.97	0.011 (0.009)	0.000	0.003 (0.004)	0.0
AA 5-mers	**0.749 (0.142)**	**0.444**	**0.916 (0.026)**	**0.958**	**0.963 (0.016)**	**0.988**	0.003 (0.003)	0.000	0.001 (0.002)	0.0
Gene content	0.893 (0.096)	0.651	0.867 (0.027)	0.922	0.937 (0.01)	0.97	0.015 (0.008)	0.000	0.0 (0.0)	0.0
SNP	1.255 (0.121)	0.923	0.831 (0.017)	0.849	0.898 (0.01)	0.946	0.031 (0.009)	0.018	0.0 (0.0)	0.0
Gene + SNP	0.89 (0.123)	0.84	0.891 (0.026)	0.898	0.951 (0.015)	0.952	0.006 (0.007)	0.000	0.0 (0.0)	0.0

**Table A44:** *K. pneumoniae*–cefuroximesodium.

	RMSE-CV	RMSE-H	DD1-CV	DD1-H	DD2-CV	DD2-H	ME-CV	ME-H	VME-CV	VME-H
NT 8-mers	0.335 (0.069)	0.391	0.978 (0.007)	0.968	0.99 (0.006)	0.987	0.0 (0.0)	0.0	0.001 (0.002)	0.000
NT 9-mers	0.34 (0.065)	0.366	0.975 (0.009)	0.968	0.992 (0.005)	0.994	0.001 (0.002)	0.0	0.0 (0.0)	0.000
NT 10-mers	0.325 (0.079)	0.471	0.981 (0.009)	0.962	0.992 (0.007)	0.981	0.0 (0.0)	0.0	0.0 (0.0)	0.000
NT 11-mers	**0.322 (0.048)**	**0.361**	**0.982 (0.009)**	0.975	0.991 (0.006)	0.994	0.0 (0.0)	0.0	0.0 (0.0)	0.000
AA 3-mers	0.362 (0.064)	0.378	0.968 (0.011)	0.968	0.992 (0.008)	0.987	0.0 (0.0)	0.0	0.0 (0.0)	0.000
AA 4-mers	0.323 (0.048)	0.422	0.978 (0.006)	0.968	0.992 (0.005)	0.987	0.0 (0.0)	0.0	0.0 (0.0)	0.000
AA 5-mers	0.327 (0.05)	0.475	0.98 (0.008)	0.962	**0.993 (0.004)**	0.981	0.001 (0.002)	0.0	0.0 (0.0)	0.006
Gene content	0.377 (0.059)	0.424	0.968 (0.008)	0.955	0.989 (0.007)	0.987	0.0 (0.0)	0.0	0.0 (0.0)	0.000
SNP	0.499 (0.063)	0.477	0.933 (0.014)	0.911	0.982 (0.012)	0.994	0.0 (0.0)	0.0	0.0 (0.0)	0.000
Gene + SNP	0.398 (0.069)	0.371	0.968 (0.011)	0.975	0.986 (0.009)	0.987	0.0 (0.0)	0.0	0.0 (0.0)	0.000

**Table A45:** *K. pneumoniae*–cefazolin.

	RMSE-CV	RMSE-H	DD1-CV	DD1-H	DD2-CV	DD2-H	ME-CV	ME-H	VME-CV	VME-H
NT 8-mers	0.658 (0.132)	**0.249**	0.932 (0.011)	**0.988**	0.966 (0.015)	**1.0**	0.005 (0.005)	0.000	0.013 (0.008)	**0.0**
NT 9-mers	0.627 (0.125)	0.442	0.928 (0.015)	0.934	0.966 (0.014)	0.994	0.003 (0.003)	0.000	0.021 (0.013)	0.018
NT 10-mers	0.662 (0.1)	0.403	0.921 (0.014)	0.964	0.965 (0.009)	0.994	0.001 (0.003)	0.000	0.019 (0.009)	0.006
NT 11-mers	0.628 (0.083)	0.475	0.927 (0.011)	0.952	0.968 (0.01)	0.982	0.001 (0.002)	0.000	0.024 (0.01)	0.024
AA 3-mers	0.814 (0.069)	0.582	0.915 (0.01)	0.934	0.951 (0.009)	0.976	0.007 (0.006)	0.000	0.021 (0.012)	0.006
AA 4-mers	0.658 (0.102)	0.416	0.939 (0.02)	0.952	0.963 (0.013)	0.988	0.002 (0.003)	0.000	0.014 (0.011)	0.012
AA 5-mers	**0.592 (0.136)**	0.445	0.939 (0.017)	0.964	**0.971 (0.015)**	0.988	0.001 (0.003)	0.000	0.015 (0.01)	0.006
Gene content	0.627 (0.154)	0.569	0.937 (0.024)	0.952	0.969 (0.014)	0.982	0.003 (0.005)	0.000	**0.011 (0.007)**	0.012
SNP	0.847 (0.094)	0.628	0.898 (0.023)	0.934	0.947 (0.012)	0.958	0.008 (0.005)	0.006	0.037 (0.02)	0.042
Gene + SNP	0.719 (0.161)	0.406	0.928 (0.024)	0.952	0.958 (0.02)	0.994	0.009 (0.007)	0.000	0.017 (0.013)	0.018

**Table A46:** *K. pneumoniae*–cefepime.

	RMSE-CV	RMSE-H	DD1-CV	DD1-H	DD2-CV	DD2-H	ME-CV	ME-H	VME-CV	VME-H
NT 8-mers	1.961 (0.075)	1.744	0.434 (0.028)	0.452	0.679 (0.021)	0.726	0.03 (0.008)	0.013	0.018 (0.012)	0.006
NT 9-mers	1.91 (0.067)	1.73	0.458 (0.027)	0.478	0.698 (0.024)	0.701	0.024 (0.007)	0.006	0.018 (0.008)	0.006
NT 10-mers	1.871 (0.064)	1.796	0.481 (0.03)	0.503	0.721 (0.016)	0.72	0.025 (0.013)	0.006	0.018 (0.005)	0.006
NT 11-mers	1.842 (0.079)	1.677	0.493 (0.039)	0.484	0.722 (0.019)	0.732	0.028 (0.012)	0.013	0.015 (0.014)	0.000
AA 3-mers	1.982 (0.077)	1.793	0.41 (0.024)	0.420	0.665 (0.042)	0.72	0.027 (0.009)	0.006	0.014 (0.011)	0.006
AA 4-mers	1.863 (0.089)	1.617	0.472 (0.031)	0.522	0.716 (0.025)	0.777	0.027 (0.013)	0.006	0.014 (0.01)	0.000
AA 5-mers	**1.786 (0.066)**	1.583	**0.506 (0.033)**	0.510	**0.74 (0.032)**	0.783	0.025 (0.015)	0.006	0.011 (0.009)	0.000
Gene content	1.814 (0.059)	1.556	0.448 (0.031)	0.522	0.713 (0.022)	0.745	**0.023 (0.008)**	0.013	**0.005 (0.006)**	0.000
SNP	1.947 (0.109)	1.67	0.421 (0.036)	0.471	0.676 (0.039)	0.732	0.025 (0.016)	0.006	0.013 (0.013)	0.006
Gene + SNP	1.856 (0.112)	**1.554**	0.455 (0.036)	0.522	0.711 (0.045)	**0.796**	0.028 (0.012)	0.006	0.01 (0.006)	0.006

**Table A47:** *K. pneumoniae*–ciprofloxacin.

	RMSE-CV	RMSE-H	DD1-CV	DD1-H	DD2-CV	DD2-H	ME-CV	ME-H	VME-CV	VME-H
NT 8-mers	0.529 (0.059)	0.579	0.914 (0.019)	0.898	0.985 (0.01)	0.970	0.0 (0.0)	0.0	0.0 (0.0)	0.0
NT 9-mers	0.514 (0.043)	0.553	0.927 (0.014)	0.922	0.984 (0.009)	0.976	0.0 (0.0)	0.0	0.0 (0.0)	0.0
NT 10-mers	0.473 (0.051)	0.541	0.933 (0.015)	0.922	0.99 (0.009)	0.988	0.0 (0.0)	0.0	0.0 (0.0)	0.0
NT 11-mers	0.392 (0.056)	0.433	0.958 (0.014)	0.940	0.993 (0.006)	0.994	0.0 (0.0)	0.0	0.0 (0.0)	0.0
AA 3-mers	0.571 (0.058)	0.66	0.901 (0.02)	0.880	0.978 (0.01)	0.970	0.0 (0.0)	0.0	0.0 (0.0)	0.0
AA 4-mers	0.444 (0.033)	0.407	0.947 (0.017)	0.958	0.99 (0.006)	0.988	0.0 (0.0)	0.0	0.0 (0.0)	0.0
AA 5-mers	**0.349 (0.056)**	**0.405**	**0.966 (0.008)**	0.958	**0.996 (0.004)**	0.994	0.0 (0.0)	0.0	0.0 (0.0)	0.0
Gene content	0.441 (0.062)	0.548	0.944 (0.016)	0.886	0.992 (0.007)	0.982	0.0 (0.0)	0.0	0.0 (0.0)	0.0
SNP	0.526 (0.058)	0.541	0.917 (0.017)	0.904	0.985 (0.008)	0.982	0.0 (0.0)	0.0	0.0 (0.0)	0.0
Gene + SNP	0.411 (0.05)	0.479	0.959 (0.015)	0.928	0.987 (0.01)	0.982	0.0 (0.0)	0.0	0.0 (0.0)	0.0

**Table A48:** *K. pneumoniae*–piperacillin.

	RMSE-CV	RMSE-H	DD1-CV	DD1-H	DD2-CV	DD2-H	ME-CV	ME-H	VME-CV	VME-H
NT 8-mers	1.377 (0.048)	1.36	0.67 (0.027)	0.657	0.844 (0.028)	0.837	0.003 (0.003)	0.0	0.026 (0.013)	0.024
NT 9-mers	1.33 (0.067)	1.306	0.678 (0.023)	0.639	0.856 (0.016)	0.843	0.003 (0.003)	0.0	0.027 (0.016)	0.006
NT 10-mers	1.334 (0.072)	1.306	0.695 (0.028)	0.645	0.856 (0.021)	0.873	0.003 (0.004)	0.0	0.022 (0.012)	0.018
NT 11-mers	1.296 (0.078)	1.269	**0.696 (0.021)**	**0.675**	0.865 (0.021)	0.861	0.001 (0.002)	0.0	0.019 (0.012)	0.012
AA 3-mers	1.36 (0.101)	**1.198**	0.658 (0.015)	0.62	0.839 (0.023)	**0.892**	0.003 (0.004)	0.0	0.017 (0.014)	**0.0**
AA 4-mers	1.262 (0.072)	1.246	0.688 (0.019)	0.669	0.872 (0.022)	0.873	0.001 (0.003)	0.0	0.013 (0.012)	0.006
AA 5-mers	1.241 (0.075)	1.264	0.693 (0.021)	0.669	**0.882 (0.015)**	0.88	0.003 (0.003)	0.0	0.014 (0.009)	0.012
Gene content	**1.218 (0.099)**	1.296	0.689 (0.028)	0.645	0.881 (0.024)	0.88	0.001 (0.003)	0.0	**0.011 (0.01)**	0.012
SNP	1.318 (0.098)	1.385	0.678 (0.019)	0.663	0.86 (0.027)	0.843	0.003 (0.004)	0.0	0.02 (0.014)	0.024
Gene + SNP	1.243 (0.097)	1.285	0.693 (0.027)	0.663	0.878 (0.024)	0.831	0.003 (0.003)	0.0	0.013 (0.008)	0.006

**Table A49:** *K. pneumoniae*–trimethoprim.

	RMSE-CV	RMSE-H	DD1-CV	DD1-H	DD2-CV	DD2-H	ME-CV	ME-H	VME-CV	VME-H
NT 8-mers	0.719 (0.069)	0.7	0.848 (0.027)	0.88	0.973 (0.011)	0.958	0.0 (0.0)	0.0	0.0 (0.0)	0.0
NT 9-mers	0.66 (0.056)	0.621	0.887 (0.017)	0.904	0.972 (0.012)	**0.988**	0.0 (0.0)	0.0	0.0 (0.0)	0.0
NT 10-mers	0.601 (0.065)	**0.551**	0.911 (0.022)	0.922	0.975 (0.01)	0.982	0.0 (0.0)	0.0	0.0 (0.0)	0.0
NT 11-mers	0.601 (0.061)	0.632	0.92 (0.019)	0.886	0.973 (0.012)	0.97	0.0 (0.0)	0.0	0.0 (0.0)	0.0
AA 3-mers	0.763 (0.048)	0.728	0.837 (0.027)	0.849	0.968 (0.013)	0.97	0.0 (0.0)	0.0	0.0 (0.0)	0.0
AA 4-mers	0.621 (0.055)	0.611	0.908 (0.018)	0.91	0.975 (0.009)	0.982	0.0 (0.0)	0.0	0.0 (0.0)	0.0
AA 5-mers	0.588 (0.066)	0.554	0.917 (0.02)	**0.928**	0.976 (0.01)	0.982	0.0 (0.0)	0.0	0.0 (0.0)	0.0
Gene content	**0.579 (0.07)**	0.583	0.927 (0.02)	0.91	0.976 (0.013)	0.982	0.0 (0.0)	0.0	0.0 (0.0)	0.0
SNP	0.736 (0.057)	0.697	0.852 (0.027)	0.867	0.965 (0.012)	0.976	0.0 (0.0)	0.0	0.0 (0.0)	0.0
Gene + SNP	0.602 (0.081)	0.605	**0.928 (0.025)**	0.916	0.969 (0.015)	0.976	0.0 (0.0)	0.0	0.0 (0.0)	0.0

### Appendix E.4. S. enterica

**Table A50:** *S. enterica*–amoxicillinclavulanicacid.

	RMSE-CV	RMSE-H	DD1-CV	DD1-H	DD2-CV	DD2-H	ME-CV	ME-H	VME-CV	VME-H
NT 8-mers	0.961 (0.097)	0.881	0.843 (0.026)	0.869	0.943 (0.015)	0.949	0.002 (0.002)	0.000	0.013 (0.005)	0.009
NT 9-mers	0.768 (0.106)	0.694	0.911 (0.015)	0.934	0.97 (0.006)	0.975	0.002 (0.002)	0.000	0.008 (0.004)	0.006
NT 10-mers	0.626 (0.106)	0.73	0.962 (0.009)	0.954	0.984 (0.007)	0.983	**0.001 (0.002)**	0.006	0.004 (0.004)	0.006
NT 11-mers	**0.608 (0.135)**	0.625	0.964 (0.008)	0.962	**0.986 (0.007)**	0.985	0.002 (0.002)	0.000	0.003 (0.003)	0.006
AA 3-mers	1.01 (0.083)	0.902	0.813 (0.021)	0.85	0.935 (0.013)	0.958	0.002 (0.002)	0.000	0.013 (0.006)	0.011
AA 4-mers	0.65 (0.136)	0.615	0.957 (0.015)	0.958	0.984 (0.009)	0.981	0.002 (0.002)	0.000	0.003 (0.003)	0.004
AA 5-mers	0.643 (0.129)	0.563	**0.967 (0.009)**	0.962	0.984 (0.007)	0.987	0.003 (0.002)	0.000	0.004 (0.003)	0.004
Gene content	0.634 (0.132)	**0.509**	0.958 (0.01)	**0.981**	0.984 (0.007)	**0.994**	0.002 (0.002)	0.002	0.004 (0.002)	**0.002**
SNP	1.449 (0.081)	1.37	0.763 (0.022)	0.772	0.877 (0.017)	0.879	0.003 (0.002)	0.002	0.055 (0.011)	0.036
Gene + SNP	0.627 (0.115)	0.693	0.955 (0.012)	0.956	0.985 (0.005)	0.977	0.002 (0.002)	0.000	0.003 (0.003)	0.008

**Table A51:** *S. enterica*–ampicillin.

	RMSE-CV	RMSE-H	DD1-CV	DD1-H	DD2-CV	DD2-H	ME-CV	ME-H	VME-CV	VME-H
NT 8-mers	1.25 (0.094)	0.577	0.784 (0.03)	0.952	0.906 (0.022)	0.983	0.003 (0.003)	0.000	0.031 (0.01)	0.004
NT 9-mers	1.009 (0.096)	0.665	0.864 (0.021)	0.958	0.942 (0.011)	0.977	0.003 (0.003)	0.000	0.016 (0.006)	0.01
NT 10-mers	0.817 (0.111)	**0.456**	0.935 (0.012)	0.975	0.976 (0.005)	0.989	0.003 (0.003)	0.002	0.011 (0.006)	**0.002**
NT 11-mers	0.763 (0.097)	0.593	0.946 (0.011)	0.981	0.98 (0.004)	0.99	0.004 (0.002)	0.002	0.008 (0.005)	0.006
AA 3-mers	1.32 (0.122)	0.906	0.747 (0.038)	0.838	0.892 (0.022)	0.952	0.004 (0.003)	0.000	0.036 (0.013)	0.01
AA 4-mers	0.824 (0.114)	0.669	0.941 (0.012)	0.964	0.975 (0.01)	0.979	0.005 (0.002)	0.000	0.01 (0.005)	0.01
AA 5-mers	**0.758 (0.116)**	0.526	**0.959 (0.008)**	0.981	**0.983 (0.006)**	0.99	0.005 (0.003)	0.000	0.008 (0.005)	0.004
Gene content	0.787 (0.1)	0.484	0.948 (0.007)	0.981	0.979 (0.006)	**0.992**	0.004 (0.002)	0.000	0.01 (0.005)	0.006
SNP	1.532 (0.073)	1.079	0.76 (0.025)	0.854	0.875 (0.02)	0.93	0.004 (0.003)	0.000	0.06 (0.009)	0.034
Gene + SNP	0.762 (0.086)	0.583	0.953 (0.007)	0.966	0.979 (0.006)	0.989	0.003 (0.002)	0.002	0.01 (0.004)	0.006

**Table A52:** *S. enterica*–azithromycin.

	RMSE-CV	RMSE-H	DD1-CV	DD1-H	DD2-CV	DD2-H	ME-CV	ME-H	VME-CV	VME-H
NT 8-mers	0.527 (0.025)	0.454	0.94 (0.005)	0.954	0.996 (0.004)	1.000	0.0 (0.0)	0.0	0.003 (0.002)	0.000
NT 9-mers	0.532 (0.032)	0.406	0.938 (0.018)	0.975	0.996 (0.003)	1.000	0.0 (0.0)	0.0	0.003 (0.002)	0.000
NT 10-mers	0.526 (0.025)	0.404	0.942 (0.006)	0.975	0.998 (0.002)	0.996	0.0 (0.0)	0.0	0.003 (0.002)	0.000
NT 11-mers	0.509 (0.028)	0.516	0.95 (0.014)	0.950	0.997 (0.002)	1.000	0.0 (0.0)	0.0	0.003 (0.002)	0.000
AA 3-mers	0.531 (0.03)	0.447	0.933 (0.013)	0.979	0.996 (0.003)	1.000	0.0 (0.0)	0.0	0.003 (0.002)	0.004
AA 4-mers	0.514 (0.03)	0.405	0.943 (0.013)	0.983	0.998 (0.003)	1.000	0.0 (0.0)	0.0	0.003 (0.002)	0.000
AA 5-mers	**0.506 (0.035)**	0.471	0.95 (0.011)	0.950	0.998 (0.002)	1.000	0.0 (0.0)	0.0	0.003 (0.002)	0.004
Gene content	0.51 (0.032)	0.388	0.941 (0.009)	0.983	**0.999 (0.002)**	0.996	0.0 (0.0)	0.0	**0.001 (0.002)**	0.000
SNP	0.521 (0.03)	0.424	0.943 (0.016)	0.967	0.996 (0.003)	1.000	0.0 (0.0)	0.0	0.003 (0.002)	0.000
Gene + SNP	0.522 (0.027)	**0.356**	0.94 (0.015)	0.983	0.996 (0.003)	1.000	0.0 (0.0)	0.0	0.003 (0.002)	0.000

**Table A53:** *S. enterica*–cefoxitin.

	RMSE-CV	RMSE-H	DD1-CV	DD1-H	DD2-CV	DD2-H	ME-CV	ME-H	VME-CV	VME-H
NT 8-mers	0.774 (0.045)	0.831	0.868 (0.019)	0.858	0.975 (0.008)	0.97	0.001 (0.001)	0.004	0.011 (0.004)	0.009
NT 9-mers	0.684 (0.048)	0.63	0.895 (0.015)	0.909	0.985 (0.007)	0.994	0.001 (0.001)	0.004	0.005 (0.003)	0.000
NT 10-mers	0.65 (0.067)	**0.528**	0.91 (0.015)	**0.934**	0.989 (0.007)	**0.998**	0.002 (0.002)	0.000	0.004 (0.003)	0.000
NT 11-mers	0.629 (0.051)	0.597	0.917 (0.01)	0.915	0.99 (0.004)	0.994	0.001 (0.001)	0.002	0.004 (0.003)	0.000
AA 3-mers	0.765 (0.046)	0.793	0.865 (0.017)	0.879	0.976 (0.005)	0.968	0.001 (0.002)	0.002	0.007 (0.003)	0.011
AA 4-mers	**0.617 (0.052)**	0.77	0.919 (0.013)	0.92	**0.992 (0.005)**	0.979	0.001 (0.001)	0.008	**0.003 (0.003)**	0.008
AA 5-mers	0.621 (0.061)	0.622	**0.926 (0.006)**	0.919	0.99 (0.005)	0.996	0.002 (0.002)	0.004	0.004 (0.003)	0.000
Gene content	0.624 (0.037)	0.58	0.924 (0.01)	0.909	0.99 (0.005)	0.992	0.002 (0.001)	0.000	0.004 (0.003)	0.002
SNP	1.163 (0.058)	1.211	0.768 (0.013)	0.765	0.915 (0.014)	0.907	0.001 (0.001)	0.004	0.045 (0.012)	0.038
Gene + SNP	0.622 (0.046)	0.621	0.923 (0.009)	0.911	0.991 (0.005)	0.987	0.001 (0.001)	0.002	0.004 (0.003)	0.002

**Table A54:** *S. enterica*–ceftiofur.

	RMSE-CV	RMSE-H	DD1-CV	DD1-H	DD2-CV	DD2-H	ME-CV	ME-H	VME-CV	VME-H
NT 8-mers	0.724 (0.079)	0.739	0.899 (0.016)	0.905	0.974 (0.012)	0.975	0.001 (0.001)	0.004	0.01 (0.006)	0.009
NT 9-mers	0.61 (0.053)	0.574	0.94 (0.013)	0.956	0.988 (0.007)	0.992	0.002 (0.002)	0.004	0.004 (0.002)	0.004
NT 10-mers	0.573 (0.053)	**0.452**	0.958 (0.006)	0.966	0.992 (0.004)	**1.0**	0.002 (0.002)	0.000	0.005 (0.003)	**0.0**
NT 11-mers	0.548 (0.053)	0.522	0.961 (0.009)	0.964	0.993 (0.004)	0.994	0.002 (0.002)	0.004	0.004 (0.003)	0.002
AA 3-mers	0.71 (0.075)	0.7	0.897 (0.021)	0.892	0.977 (0.009)	0.983	0.002 (0.002)	0.000	0.007 (0.004)	0.008
AA 4-mers	0.56 (0.065)	0.583	0.96 (0.006)	0.954	0.992 (0.005)	0.992	0.002 (0.003)	0.002	0.005 (0.003)	0.006
AA 5-mers	**0.546 (0.057)**	0.553	**0.965 (0.008)**	**0.975**	0.993 (0.004)	0.992	0.003 (0.003)	0.000	0.004 (0.003)	0.008
Gene content	0.553 (0.051)	0.537	0.961 (0.005)	0.964	0.993 (0.005)	0.994	0.002 (0.002)	0.002	0.005 (0.003)	0.004
SNP	1.161 (0.058)	1.147	0.777 (0.014)	0.789	0.909 (0.014)	0.911	0.003 (0.003)	0.000	0.042 (0.011)	0.047
Gene + SNP	0.549 (0.051)	0.646	0.958 (0.007)	0.954	0.993 (0.003)	0.985	0.001 (0.001)	0.006	0.004 (0.003)	0.008

**Table A55:** *S. enterica*–ceftriaxone.

	RMSE-CV	RMSE-H	DD1-CV	DD1-H	DD2-CV	DD2-H	ME-CV	ME-H	VME-CV	VME-H
NT 8-mers	0.965 (0.141)	0.615	0.893 (0.014)	0.951	0.953 (0.011)	0.979	0.004 (0.003)	0.002	0.012 (0.007)	0.004
NT 9-mers	0.782 (0.107)	0.408	0.928 (0.012)	0.975	0.975 (0.007)	0.994	0.003 (0.002)	0.000	0.007 (0.003)	0.002
NT 10-mers	0.647 (0.122)	0.541	0.958 (0.011)	0.966	0.986 (0.006)	0.989	0.003 (0.002)	0.002	0.006 (0.004)	0.004
NT 11-mers	0.671 (0.132)	0.292	0.959 (0.006)	**0.992**	0.986 (0.005)	**0.998**	0.003 (0.003)	0.000	0.006 (0.003)	0.002
AA 3-mers	0.919 (0.077)	0.674	0.891 (0.016)	0.951	0.957 (0.007)	0.975	0.004 (0.002)	0.002	0.009 (0.003)	0.004
AA 4-mers	0.66 (0.12)	0.531	0.954 (0.006)	0.968	0.985 (0.005)	0.989	0.003 (0.002)	0.002	0.006 (0.004)	0.004
AA 5-mers	0.673 (0.123)	0.292	**0.963 (0.009)**	0.987	0.986 (0.005)	0.994	0.003 (0.003)	0.000	0.006 (0.003)	0.000
Gene content	**0.644 (0.1)**	0.402	0.961 (0.008)	0.979	**0.987 (0.004)**	0.994	**0.002 (0.002)**	0.002	0.006 (0.003)	0.000
SNP	1.588 (0.098)	1.580	0.78 (0.021)	0.808	0.875 (0.016)	0.89	0.006 (0.004)	0.006	0.046 (0.009)	0.051
Gene + SNP	0.648 (0.133)	0.700	0.955 (0.008)	0.96	0.985 (0.006)	0.979	0.003 (0.002)	0.000	0.006 (0.004)	0.011

**Table A56:** *S. enterica*–chloramphenicol.

	RMSE-CV	RMSE-H	DD1-CV	DD1-H	DD2-CV	DD2-H	ME-CV	ME-H	VME-CV	VME-H
NT 8-mers	0.495 (0.035)	0.437	0.962 (0.012)	0.977	0.994 (0.002)	1.000	0.001 (0.001)	0.000	0.001 (0.001)	0.000
NT 9-mers	0.473 (0.03)	0.45	0.963 (0.022)	0.977	0.995 (0.002)	0.998	0.001 (0.001)	0.002	0.001 (0.001)	0.000
NT 10-mers	0.456 (0.025)	0.436	0.973 (0.005)	0.975	0.997 (0.001)	0.998	0.0 (0.001)	0.000	0.001 (0.001)	0.000
NT 11-mers	0.45 (0.019)	**0.413**	0.972 (0.002)	0.979	0.997 (0.002)	1.000	0.0 (0.001)	0.000	0.001 (0.001)	0.000
AA 3-mers	0.507 (0.045)	0.449	0.958 (0.01)	0.979	0.993 (0.003)	1.000	0.0 (0.001)	0.000	0.002 (0.002)	0.000
AA 4-mers	0.459 (0.029)	0.445	0.967 (0.016)	**0.991**	0.997 (0.002)	0.998	0.0 (0.001)	0.002	0.001 (0.002)	0.000
AA 5-mers	**0.435 (0.023)**	0.423	**0.979 (0.005)**	0.977	0.998 (0.002)	0.998	0.0 (0.001)	0.000	0.001 (0.001)	0.002
Gene content	0.443 (0.028)	0.427	0.977 (0.005)	0.985	0.998 (0.002)	0.996	0.0 (0.0)	0.002	0.001 (0.002)	0.002
SNP	0.521 (0.048)	0.486	0.956 (0.024)	0.97	0.99 (0.003)	0.994	0.002 (0.003)	0.000	0.002 (0.002)	0.002
Gene + SNP	0.446 (0.025)	0.449	0.978 (0.005)	0.975	0.997 (0.002)	0.998	0.0 (0.001)	0.000	0.001 (0.001)	0.002

**Table A57:** *S. enterica*–ciprofloxacin.

	RMSE-CV	RMSE-H	DD1-CV	DD1-H	DD2-CV	DD2-H	ME-CV	ME-H	VME-CV	VME-H
NT 8-mers	0.64 (0.048)	0.54	0.902 (0.016)	0.932	0.987 (0.004)	0.991	0.0 (0.0)	0.0	0.0 (0.001)	0.0
NT 9-mers	0.628 (0.045)	0.58	0.906 (0.015)	0.915	0.987 (0.005)	0.991	0.0 (0.0)	0.0	0.001 (0.001)	0.0
NT 10-mers	0.604 (0.039)	0.51	0.907 (0.009)	0.92	0.989 (0.003)	0.992	0.0 (0.0)	0.0	0.001 (0.001)	0.0
NT 11-mers	0.561 (0.034)	0.458	0.913 (0.008)	0.953	0.991 (0.003)	0.994	0.0 (0.0)	0.0	0.0 (0.001)	0.0
AA 3-mers	0.65 (0.035)	0.585	0.903 (0.014)	0.901	0.987 (0.003)	0.991	0.0 (0.001)	0.0	0.0 (0.001)	0.0
AA 4-mers	0.617 (0.038)	0.489	0.906 (0.014)	0.922	0.987 (0.003)	0.996	0.0 (0.0)	0.0	0.0 (0.001)	0.0
AA 5-mers	**0.541 (0.036)**	**0.437**	**0.921 (0.012)**	0.947	**0.992 (0.003)**	**0.998**	0.0 (0.0)	0.0	0.0 (0.0)	0.0
Gene content	0.618 (0.061)	0.517	0.904 (0.01)	0.935	0.987 (0.004)	0.994	0.0 (0.0)	0.0	0.0 (0.0)	0.0
SNP	0.611 (0.036)	0.542	0.909 (0.017)	0.937	0.989 (0.004)	0.992	0.0 (0.0)	0.0	0.001 (0.001)	0.0
Gene + SNP	0.572 (0.057)	0.495	0.91 (0.015)	**0.956**	0.99 (0.004)	0.992	0.0 (0.0)	0.0	0.0 (0.0)	0.0

**Table A58:** *S. enterica*–gentamicin.

	RMSE-CV	RMSE-H	DD1-CV	DD1-H	DD2-CV	DD2-H	ME-CV	ME-H	VME-CV	VME-H
NT 8-mers	1.123 (0.097)	0.978	0.806 (0.033)	0.848	0.932 (0.016)	0.956	0.001 (0.001)	0.002	0.024 (0.008)	0.006
NT 9-mers	0.989 (0.083)	0.77	0.847 (0.026)	0.888	0.958 (0.009)	0.985	0.002 (0.002)	0.002	0.014 (0.007)	0.002
NT 10-mers	0.901 (0.077)	0.846	0.866 (0.026)	0.888	0.97 (0.01)	0.968	0.002 (0.002)	**0.0**	0.011 (0.004)	0.006
NT 11-mers	**0.787 (0.063)**	0.972	0.89 (0.014)	0.911	0.981 (0.006)	0.975	0.001 (0.001)	0.004	0.005 (0.003)	0.011
AA 3-mers	1.172 (0.108)	0.94	0.791 (0.033)	0.843	0.928 (0.018)	0.962	0.002 (0.002)	0.008	0.026 (0.009)	0.004
AA 4-mers	0.866 (0.085)	0.773	0.88 (0.017)	0.898	0.975 (0.008)	0.981	0.002 (0.001)	0.002	0.009 (0.003)	0.000
AA 5-mers	0.797 (0.051)	**0.745**	**0.895 (0.013)**	0.905	**0.983 (0.004)**	**0.991**	0.002 (0.002)	0.002	0.005 (0.003)	0.004
Gene content	0.81 (0.06)	0.766	0.892 (0.015)	**0.926**	0.981 (0.005)	0.989	0.001 (0.001)	0.004	0.006 (0.003)	0.000
SNP	1.093 (0.088)	1.057	0.843 (0.021)	0.865	0.952 (0.009)	0.964	0.005 (0.005)	0.006	0.018 (0.005)	0.013
Gene + SNP	0.795 (0.055)	0.914	0.889 (0.017)	0.89	0.982 (0.005)	0.972	0.001 (0.001)	0.004	0.005 (0.003)	0.011

**Table A59:** *S. enterica*–kanamycin.

	RMSE-CV	RMSE-H	DD1-CV	DD1-H	DD2-CV	DD2-H	ME-CV	ME-H	VME-CV	VME-H
NT 8-mers	0.788 (0.155)	0.714	0.901 (0.021)	0.912	0.957 (0.024)	0.956	0.005 (0.006)	0.000	0.023 (0.017)	0.022
NT 9-mers	0.525 (0.167)	0.484	0.951 (0.02)	0.967	0.981 (0.016)	0.978	0.0 (0.0)	0.000	0.011 (0.014)	0.011
NT 10-mers	0.423 (0.148)	0.345	0.965 (0.018)	0.967	0.989 (0.008)	1.000	0.0 (0.0)	0.000	0.006 (0.008)	0.000
NT 11-mers	0.401 (0.188)	0.155	0.981 (0.013)	1.000	0.988 (0.011)	1.000	0.001 (0.004)	0.000	0.01 (0.012)	0.000
AA 3-mers	0.641 (0.157)	0.554	0.932 (0.024)	0.945	0.97 (0.021)	0.978	0.002 (0.005)	0.000	0.014 (0.014)	0.022
AA 4-mers	0.458 (0.102)	0.393	0.972 (0.013)	0.989	0.985 (0.007)	0.989	0.001 (0.004)	0.000	0.008 (0.006)	0.011
AA 5-mers	**0.321 (0.179)**	0.15	**0.986 (0.013)**	0.989	**0.993 (0.01)**	1.000	0.0 (0.0)	0.000	0.006 (0.008)	0.000
Gene content	0.402 (0.14)	**0.126**	0.98 (0.019)	1.000	0.99 (0.009)	1.000	0.0 (0.0)	0.000	0.007 (0.008)	0.000
SNP	0.788 (0.175)	0.752	0.914 (0.022)	0.912	0.953 (0.021)	0.945	0.006 (0.008)	0.011	0.028 (0.026)	0.011
Gene + SNP	0.503 (0.086)	0.256	0.943 (0.025)	0.978	0.987 (0.01)	1.000	0.0 (0.0)	0.000	0.007 (0.01)	0.000

**Table A60:** *S. enterica*–nalidixic acid.

	RMSE-CV	RMSE-H	DD1-CV	DD1-H	DD2-CV	DD2-H	ME-CV	ME-H	VME-CV	VME-H
NT 8-mers	0.556 (0.026)	**0.394**	0.947 (0.013)	**0.979**	0.99 (0.003)	1.000	0.0 (0.0)	0.0	0.007 (0.003)	0.000
NT 9-mers	0.546 (0.023)	0.498	0.955 (0.006)	0.972	0.991 (0.002)	0.994	0.0 (0.001)	0.0	0.007 (0.002)	0.006
NT 10-mers	0.511 (0.023)	0.434	0.953 (0.009)	0.973	0.993 (0.002)	0.998	0.0 (0.0)	0.0	0.005 (0.002)	0.002
NT 11-mers	0.475 (0.036)	0.452	0.96 (0.011)	0.96	**0.996 (0.003)**	1.000	0.0 (0.001)	0.0	**0.002 (0.001)**	0.000
AA 3-mers	0.559 (0.035)	0.512	0.945 (0.019)	0.972	0.99 (0.002)	0.991	0.0 (0.0)	0.0	0.007 (0.002)	0.008
AA 4-mers	0.523 (0.037)	0.454	0.957 (0.006)	0.97	0.994 (0.004)	0.992	0.0 (0.0)	0.0	0.005 (0.003)	0.004
AA 5-mers	0.475 (0.031)	0.431	**0.961 (0.008)**	0.973	0.995 (0.003)	1.000	0.0 (0.0)	0.0	0.003 (0.002)	0.000
Gene content	0.539 (0.03)	0.429	0.953 (0.005)	0.973	0.992 (0.004)	0.994	0.0 (0.0)	0.0	0.007 (0.002)	0.004
SNP	0.535 (0.044)	0.438	0.954 (0.007)	0.973	0.99 (0.003)	0.998	0.0 (0.0)	0.0	0.006 (0.002)	0.000
Gene + SNP	0.513 (0.036)	0.501	0.958 (0.005)	0.964	0.995 (0.002)	0.994	0.0 (0.0)	0.0	0.005 (0.003)	0.002

**Table A61:** *S. enterica*–streptomycin.

	RMSE-CV	RMSE-H	DD1-CV	DD1-H	DD2-CV	DD2-H	ME-CV	ME-H	VME-CV	VME-H
NT 8-mers	0.87 (0.064)	0.835	0.833 (0.028)	0.842	0.956 (0.01)	0.971	0.02 (0.011)	0.022	0.021 (0.012)	0.025
NT 9-mers	0.77 (0.074)	0.731	0.867 (0.03)	0.867	0.972 (0.012)	0.978	0.012 (0.007)	0.018	0.021 (0.013)	0.022
NT 10-mers	0.742 (0.052)	0.723	0.884 (0.014)	0.896	0.975 (0.009)	0.968	0.01 (0.006)	0.014	0.023 (0.01)	0.025
NT 11-mers	0.731 (0.073)	**0.692**	0.89 (0.021)	0.9	0.976 (0.012)	0.975	0.008 (0.005)	0.011	0.021 (0.012)	0.039
AA 3-mers	0.891 (0.077)	0.824	0.811 (0.032)	0.832	0.953 (0.017)	0.961	0.022 (0.016)	0.018	0.023 (0.014)	**0.011**
AA 4-mers	0.741 (0.097)	0.712	0.89 (0.034)	0.889	0.974 (0.014)	0.975	0.009 (0.008)	**0.004**	0.026 (0.016)	0.029
AA 5-mers	**0.721 (0.072)**	0.724	0.891 (0.014)	0.896	0.974 (0.011)	0.975	0.008 (0.005)	0.007	0.026 (0.01)	0.025
Gene content	0.724 (0.078)	0.7	**0.894 (0.024)**	**0.918**	**0.978 (0.01)**	**0.982**	0.008 (0.005)	0.007	0.025 (0.015)	0.029
SNP	0.754 (0.06)	0.749	0.873 (0.019)	0.878	0.973 (0.01)	0.978	0.013 (0.007)	0.007	**0.014 (0.012)**	0.025
Gene + SNP	0.723 (0.047)	0.778	0.886 (0.013)	0.875	0.977 (0.008)	0.971	**0.007 (0.005)**	0.007	0.019 (0.013)	0.036

**Table A62:** *S. enterica*–sulfisoxazole.

	RMSE-CV	RMSE-H	DD1-CV	DD1-H	DD2-CV	DD2-H	ME-CV	ME-H	VME-CV	VME-H
NT 8-mers	0.896 (0.053)	0.812	0.813 (0.014)	0.85	0.954 (0.014)	0.963	0.001 (0.002)	0.000	0.051 (0.015)	0.020
NT 9-mers	0.798 (0.063)	0.713	0.85 (0.025)	0.856	0.971 (0.01)	0.982	0.001 (0.001)	0.002	0.032 (0.014)	0.014
NT 10-mers	0.741 (0.051)	**0.69**	0.879 (0.014)	0.884	0.978 (0.009)	0.982	0.0 (0.001)	0.000	0.018 (0.007)	0.012
NT 11-mers	0.721 (0.047)	0.702	0.879 (0.013)	**0.904**	0.98 (0.005)	0.978	0.001 (0.001)	0.000	0.014 (0.005)	0.012
AA 3-mers	0.951 (0.081)	0.869	0.792 (0.027)	0.791	0.945 (0.014)	0.972	0.001 (0.002)	0.002	0.063 (0.02)	0.028
AA 4-mers	0.738 (0.031)	0.826	0.87 (0.015)	0.856	0.979 (0.007)	0.97	0.0 (0.001)	0.002	0.019 (0.004)	0.010
AA 5-mers	**0.713 (0.035)**	0.736	**0.888 (0.009)**	0.88	0.982 (0.004)	0.976	0.001 (0.001)	0.000	**0.011 (0.006)**	0.014
Gene content	0.715 (0.044)	0.712	0.885 (0.009)	0.878	**0.983 (0.006)**	**0.986**	0.0 (0.0)	0.000	0.014 (0.003)	0.010
SNP	0.805 (0.055)	0.739	0.864 (0.013)	0.874	0.971 (0.007)	0.976	0.001 (0.001)	0.000	0.021 (0.008)	0.016
Gene + SNP	0.721 (0.041)	0.71	0.879 (0.013)	0.886	0.982 (0.005)	0.98	0.0 (0.001)	0.004	0.015 (0.006)	0.014

**Table A63:** *S. enterica*–tetracycline.

	RMSE-CV	RMSE-H	DD1-CV	DD1-H	DD2-CV	DD2-H	ME-CV	ME-H	VME-CV	VME-H
NT 8-mers	0.821 (0.135)	0.699	0.87 (0.051)	0.886	0.948 (0.027)	0.968	0.023 (0.015)	0.019	0.001 (0.001)	0.000
NT 9-mers	0.647 (0.1)	0.665	0.931 (0.034)	0.947	0.972 (0.012)	0.973	0.012 (0.007)	0.017	0.001 (0.002)	0.000
NT 10-mers	0.592 (0.087)	0.556	0.944 (0.038)	0.96	0.979 (0.006)	0.983	0.007 (0.004)	0.009	0.001 (0.001)	0.000
NT 11-mers	0.563 (0.061)	0.48	0.959 (0.006)	0.968	0.982 (0.006)	0.989	0.007 (0.003)	**0.002**	0.001 (0.002)	0.000
AA 3-mers	0.802 (0.111)	0.81	0.865 (0.052)	0.869	0.957 (0.016)	0.954	0.02 (0.009)	0.03	0.003 (0.003)	0.000
AA 4-mers	0.562 (0.07)	0.548	0.962 (0.014)	0.958	0.982 (0.005)	0.985	0.008 (0.002)	0.006	0.001 (0.001)	0.000
AA 5-mers	0.545 (0.076)	**0.402**	0.969 (0.008)	**0.985**	0.982 (0.006)	**0.992**	0.007 (0.004)	0.004	0.0 (0.001)	0.000
Gene content	**0.509 (0.081)**	0.604	**0.973 (0.01)**	0.968	**0.985 (0.006)**	0.979	**0.005 (0.002)**	0.015	0.0 (0.0)	0.000
SNP	0.869 (0.13)	0.768	0.882 (0.051)	0.899	0.941 (0.027)	0.956	0.028 (0.019)	0.015	0.001 (0.001)	0.002
Gene + SNP	0.539 (0.066)	0.597	0.963 (0.024)	0.97	0.984 (0.006)	0.979	0.006 (0.003)	0.009	0.001 (0.001)	0.000

**Table A64:** *S. enterica*–trimethoprimSulfamethoxazole.

	RMSE-CV	RMSE-H	DD1-CV	DD1-H	DD2-CV	DD2-H	ME-CV	ME-H	VME-CV	VME-H
NT 8-mers	0.578 (0.05)	0.284	0.957 (0.007)	0.983	0.988 (0.002)	0.996	0.0 (0.001)	0.000	0.008 (0.002)	0.002
NT 9-mers	0.557 (0.083)	0.469	0.957 (0.011)	0.973	0.989 (0.004)	0.994	0.0 (0.0)	0.000	0.008 (0.003)	0.004
NT 10-mers	0.506 (0.067)	0.323	0.957 (0.01)	0.972	0.989 (0.002)	0.998	0.0 (0.0)	0.000	0.007 (0.002)	0.000
NT 11-mers	0.394 (0.063)	0.327	0.968 (0.006)	0.981	0.994 (0.003)	0.998	0.001 (0.001)	0.002	0.003 (0.003)	0.000
AA 3-mers	0.573 (0.072)	0.232	0.96 (0.008)	0.987	0.988 (0.003)	1.000	0.0 (0.001)	0.000	0.009 (0.002)	0.000
AA 4-mers	0.443 (0.077)	0.247	0.964 (0.008)	0.975	0.991 (0.004)	0.998	0.0 (0.001)	0.000	0.005 (0.003)	0.002
AA 5-mers	0.349 (0.064)	**0.224**	**0.98 (0.005)**	**0.991**	0.996 (0.002)	1.000	0.0 (0.001)	0.000	**0.001 (0.001)**	0.000
Gene content	**0.346 (0.055)**	0.324	0.971 (0.01)	0.981	0.996 (0.002)	0.998	0.0 (0.001)	0.000	0.002 (0.002)	0.002
SNP	0.532 (0.09)	0.364	0.961 (0.009)	0.972	0.989 (0.003)	0.994	0.0 (0.0)	0.000	0.007 (0.003)	0.006
Gene + SNP	0.436 (0.091)	0.319	0.966 (0.009)	0.972	0.992 (0.004)	0.994	0.0 (0.0)	0.000	0.005 (0.003)	0.006

## Appendix F. Canonical and Non-Canonical Nucleotide k-mers

Using lexicographical order, nucleotide *k*-mers can be divided into two groups: canonical or non-canonical. A *k*-mer is called canonical if its lexicographical order is smaller than or equal to the order of its Watson–Crick reverse complement [57]. For example, in the case of a 3-mer like “TTT,” the lexicographical order of the Watson-Crick reverse complement, “AAA” is smaller, so “AAA” is canonical and “TTT” is non-canonical.

As mentioned in Section 2.2.1, for nucleotide *k*-mers two scenarios are possible: converting all of the non-canonical *k*-mers to their canonical counter parts and counting them after conversion, or counting every *k*-mer, canonical or otherwise [57]. We tested both scenarios and compared the results. for *k*-mers of length 8, 9, 19 and 11, results of running the model with both scenarios are presented in Appendix F, Figure A8, Figure A9, Figure A10 and Figure A11.

When non-canonical *k*-mers are converted to canonical form, the accuracy of MIC prediction becomes significantly better compared to a scenario in which all *k*-mers are counted. To investigate the reasons for this observation, we hypothesized that when all (canonical and non-canonical) *k*-mers are counted separately, counts of non-canonical *k*-mers are correlated with counts of canonical counterparts, e.g., frequency of an 8-mer like “TTTTTTTT” is correlated with frequency of “AAAAAAAA.” This hypothesis was based on the generalization of the second Chargaff rule, which states that “*k*-mer frequency counted on a single chromosomal strand equals the frequency of the reverse-complement *k*-mer,” which holds for many species in both prokaryotes and eukaryotes [122]. Having correlated features increases the feature to strain ratio, without actually adding information and worsens learning performance [123], particularly in cases when number of strains is low compared to number of features like the data that we have.

To test this hypothesis, we tried calculating the correlations between the frequencies of canonical and non-canonical features; both frequencies were counted separately (no conversion to canonical form was applied). For each *k*-mer length, in each genome, we calculated the Pearson correlation coefficient between 1000 randomly selected non-canonical features and their canonical counterparts. The distribution of correlation coefficient across the genomes is depicted in Figure A12. It can be seen that there is a strong correlation between the features. This correlation is the reason for poor performance of *k*-mer counting methods when both canonical and non-canonical *k*-mers are counted and no conversion is applied.

## Appendix G. Measuring Feature Stability in Different Methods

The stability of a feature selection algorithm refers to the robustness of its feature preferences, with respect to changes in the data. In an unstable algorithm, a small change in data leads to large changes in the chosen features [124]. In *N*-cross-validation technique, feature stability can be measured by comparing the feature preference in different folds: the feature selection algorithm is stable if the same features are selected as important features in different folds [125]. In order to measure feature stability of cross-validation, we implemented an algorithm proposed by Kalousis et al. in [125]. Briefly, this algorithm measures similarity of two sets of features by calculating the Spearman’s correlation coefficients [126] between the ranks of of the features. Since in *N*-fold cross-validation, there are *N* sets of feature ranks, the algorithm proposes calculating the correlation for all N(N−1)/2 pairwise combinations of folds and averaging over all N(N−1)/2 values to get the final value [125]. To get the ranks of the features we sorted them based on their absolute SHAP values (see Section 2.5.3).

For each feature extraction method and each species–antibiotic combination we measured the feature stability. Then, to compare feature stability of models trained with different feature extraction methods, we averaged the score of each feature extraction method over all species–antibiotic combinations. Average feature stability scores are presented in Table A65.

**Table A65:** Average feature stability scores of different methods methods across all species–antibiotic combinations.

Method	Score
NT 8-mers	0.98275
NT 9-mers	0.99636
NT 10-mers	0.99919
NT 11-mers	0.99978
AA 3-mers	0.90825
AA 4-mers	0.99675
AA 5-mers	0.99973
Gene content	0.99536
SNP	0.99952
Gene content + SNP	0.99973

Based on results of Table A65, when features are selected based on the absolute SHAP value, they become stable, using all features extraction methods, except for short *k*-mers, which are not able to capture information. For *k*-mer counting methods, feature stability increases with *k*. This was expected because longer *k*’s are more likely to capture the patterns of genome and lead to better accuracy. For amino acid 5-mers, nucleotide 11-mers, SNP and SNP + gene content pipelines, the average Pearson correlation coefficient between ranks of features of different folds was 0.999. The gene content method had a slightly lower feature stability score.

## Appendix H. Performances of the Selected-Feature Model Pipelines

To quantify performances of the selected-feature pipelines, we asked two question: First, in how many species–antibiotic combinations did the selected-feature pipeline perform better than the pipeline that used all features in terms of average cross-validation accuracy? Second, when it did, how many features did it need to reach an accuracy higher than the pipeline that used all features? For example, in case of *S. enterica* and ampicillin, the gene content pipeline reached a better accuracy than all features pipeline using 5 features (see figure in Figure 9e). Results of this analysis are presented in Table A66. In most cases, the selected-feature pipelines performed better than pipelines that used all features.

**Table A66:** Evaluation of the performance of the selected-feature pipeline, compared to the all-features pipeline.

Method	Average (STD Accuracy of All Feature Pipeline)	Ratio of Datasets Where Selected-Feature Pipeline Reaches a Better Accuracy	Average (STD) of Required Features to Reach a Better Accuracy
NT 8-mers	0.8316 (0.1112)	0.9811	11.3774 (9.2456)
NT 9-mers	0.8545 (0.0985)	0.9245	10.6604 (10.7842)
NT 10-mers	0.8723 (0.0993)	0.8679	14.283 (12.7817)
NT 11-mers	0.8784 (0.0943)	0.9245	13.4528 (12.509)
AA 3-mers	0.8233 (0.116)	0.9057	13.5094 (12.438)
AA 4-mers	0.8716 (0.0979)	0.9057	11.6038 (11.0288)
AA 5-mers	0.8849 (0.0963)	0.717	17.8491 (15.4607)
Gene content	0.8561 (0.1258)	0.6604	21.0566 (16.1986)
SNP	0.8239 (0.1101)	0.6792	19.7547 (16.4511)
Gene content + SNP	0.8696 (0.1005)	0.717	19.3962 (15.838)

## Appendix I. Resources Used for Example Datasets

In this section we mention the requested resources for one example antibiotic for each species, to give the reader a sense of computational complexity of the problem. The wall-time was for training and testing the model with all features, and training and testing the model with the selected features, which was done for different numbers of of features, as described in the methods section.

**Table A67:** Resources for *C. jejuni*–erythromycin.

Method	Number of Features	Number of Cores	Maximum Memory Usage	Wall-Clock
NT 8-mers	32,808	2	3 GB	00:25:19
NT 9-mers	124,591	2	20 GB	00:41:28
NT 10-mers	404,690	2	28 GB	01:25:03
NT 11-mers	1,029,088	2	41 GB	03:13:57
AA 3-mer	8035	2	2 GB	00:22:47
AA 4-mer	140,093	2	25 GB	00:53:54
AA 5-mer	935,293	2	38 GB	03:28:11
Gene content	14,981	2	2 GB	00:24:24
SNP	331,936	2	30 GB	01:08:55
Gene content + SNP	346,917	2	31 GB	00:56:31

**Table A68:** Resources for *N. gonhoriae*–penicillin.

Method	Number of Features	Number of Cores	Maximum Memory Usage	Wall-Clock
NT 8-mers	32,896	2	3 GB	00:27:54
NT 9-mers	131,012	2	23 GB	00:45:16
NT 10-mers	507,346	10	30 GB	00:58:07
NT 11-mers	1,588,941	10	90 GB	02:22:14
AA 3-mer	8818	2	2 GB	00:23:38
AA 4-mer	149,817	2	25 GB	00:37:49
AA 5-mer	1,057,709	5	54 GB	01:37:41
Gene content	36,458	2	3 GB	00:30:07
SNP	300,296	2	32 GB	01:09:56
Gene content + SNP	336,754	2	33 GB	01:18:53

**Table A69:** Resources for *K. pneumoniae*–amikacin.

Method	Number of Features	Number of Cores	Maximum Memory Usage	Wall-Clock
NT 8-mers	32,896	2	7 GB	01:02:34
NT 9-mers	131,072	2	23 GB	01:57:17
NT 10-mers	524,798	10	91 GB	03:08:46
NT 11-mers	2,095,237	22	309 GB	07:29:17
AA 3-mer	8091	2	2 GB	00:46:07
AA 4-mer	159,859	2	27 GB	01:39:31
AA 5-mer	2,463,537	10	283 GB	06:10:21
Gene content	129,175	2	13 GB	00:49:22
SNP	2,508,568	10	238 GB	03:58:14
Gene content + SNP	2,637,743	10	251 GB	04:29:25

**Table A70:** Resources for *S. enterica*–ampicillin.

Method	Number of Features	Number of Cores	Maximum Memory Usage	Wall-Clock
NT 8-mers	32,896	2	19 GB	02:39:14
NT 9-mers	131,072	6	73 GB	04:52:28
NT 10-mers	524,799	21	282 GB	06:47:57
NT 11-mers	2,092,713	62	1002 GB	22:52:44
AA 3-mer	9090	2	5 GB	01:40:46
AA 4-mer	164,507	4	86 GB	03:30:33
AA 5-mer	2,389,091	62	876 GB	20:32:39
Gene content	128,290	4	39 GB	01:56:19
SNP	2,472,276	10	739 GB	00:51:39
Gene content + SNP	2,600,566	10	815 GB	01:34:59

## Appendix J. Chemical Structure of Antibiotics

In this section, the chemical structures of the antibiotics and their classifications are provided. The chemical structure images were obtained from PATRIC database [127].

**Classification**	**Antibiotic Name**	**Structure**
Aminoglycosides	streptomycin	
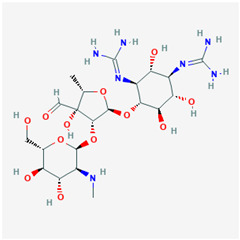
Aminoglycosides	amikacin	
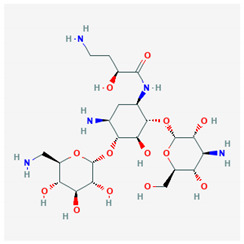
Aminoglycosides	kanamycin	
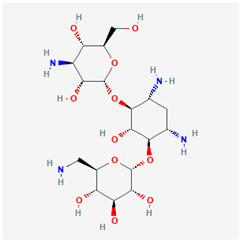
Aminoglycosides	tobramycin	
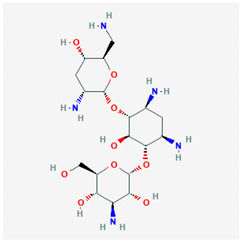
Aminoglycosides	spectinomycin	
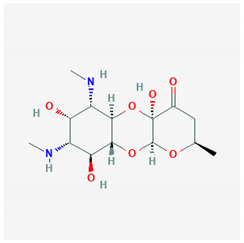
Aminoglycosides	gentamicin	
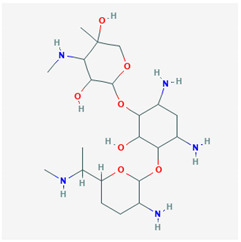
Tetracyclines	tetracycline	
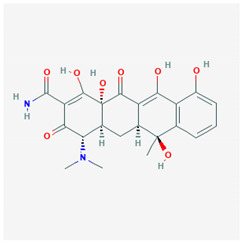
Amphenicols	chloramphenicol	
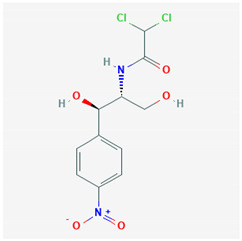
Amphenicols	florfenicol	
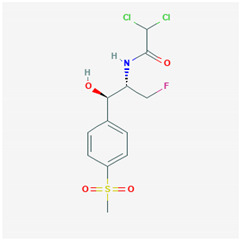
Macrolides	azithromycin	
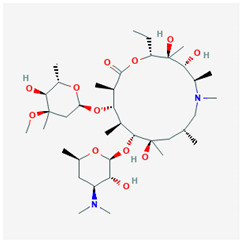
Macrolides	erythromycin	
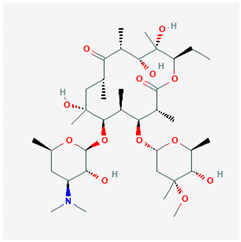
Macrolides	telithromycin	
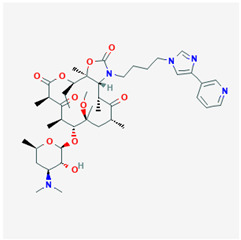
Lincosamides	clindamycin	
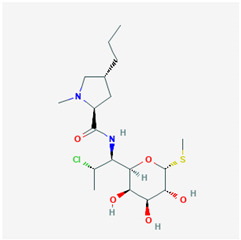
Antifolates	trimethoprim	
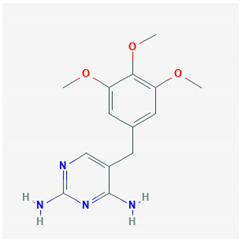
Antifolates	sulfisoxazole	
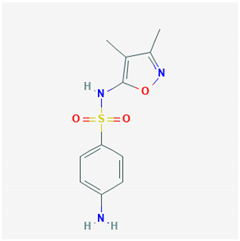
Quinolones	nalidixic acid	
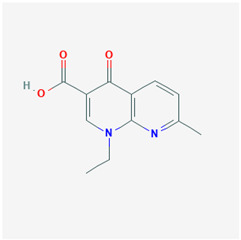
Quinolones	ciprofloxacin	
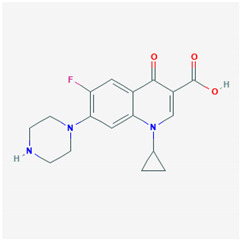
Quinolones	levofloxacin	
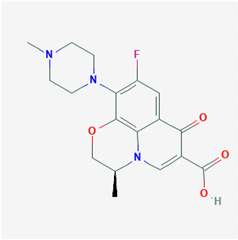
Nitrofurans	nitrofurantoin	
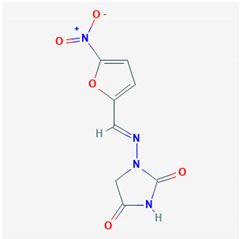
Penicillins	penicillin	
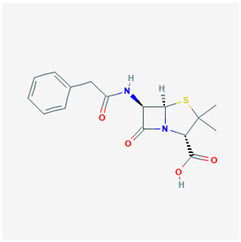
Penicillins	amoxicillin	
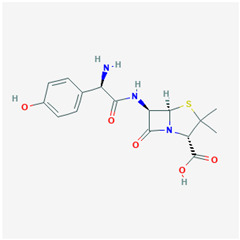
Penicillins	ampicillin	
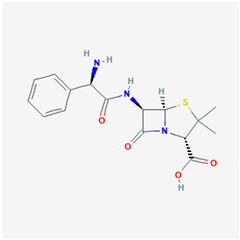
Penicillins	piperacillin	
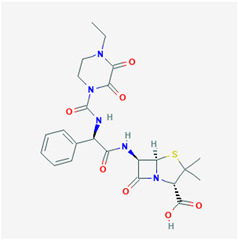
Carbapenems	imipenem	
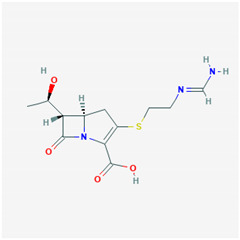
Carbapenems	meropenem	
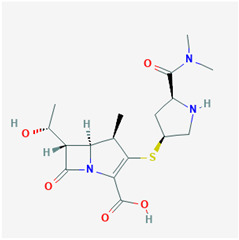
Cephalosporins/Cephamycins	cefazolin	
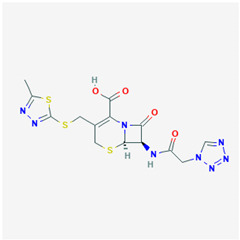
Cephalosporins/Cephamycins	ceftriaxone	
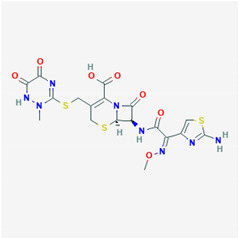
Cephalosporins/Cephamycins	cefepime	
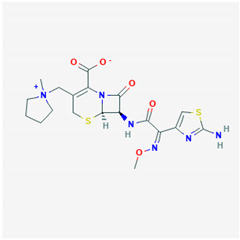
Cephalosporins/Cephamycins	cefoxitin	
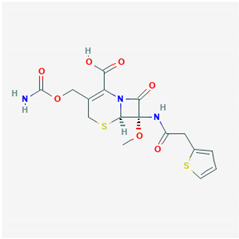
Cephalosporins/Cephamycins	cefixime	
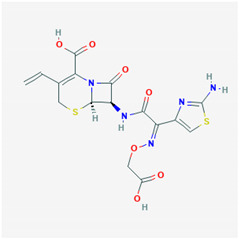
Cephalosporins/Cephamycins	ceftazidime/clavulanic acid	
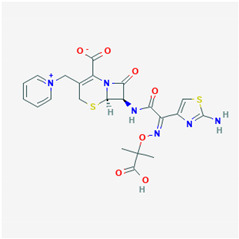
Cephalosporins/Cephamycins	cefpodoxime proxetil	
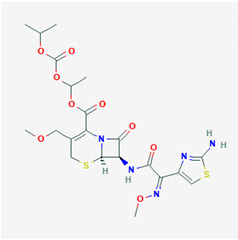
Cephalosporins/Cephamycins	ceftiofur	
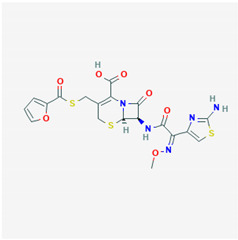
Monobactams	aztreonam	
